# Hoxb genes determine the timing of cell ingression by regulating cell surface fluctuations during zebrafish gastrulation

**DOI:** 10.1242/dev.204261

**Published:** 2025-06-27

**Authors:** Yuuta Moriyama, Toshiyuki Mitsui, Carl-Philipp Heisenberg

**Affiliations:** ^1^ Institute of Science and Technology Austria, Am Campus 1, A-3400, Klosterneuburg, Austria; ^2^ Department of Physical Sciences, College of Science and Engineering, Aoyama Gakuin University, Kanagawa 252-5258, Japan

**Keywords:** Hox, Gastrulation, Bleb, Cell surface fluctuation

## Abstract

During embryonic development, cell behaviors need to be tightly regulated in time and space. Yet how the temporal and spatial regulations of cell behaviors are interconnected during embryonic development remains elusive. To address this, we turned to zebrafish gastrulation, the process whereby dynamic cell behaviors generate the three principal germ layers of the early embryo. Here, we show that Hoxb cluster genes are expressed in a temporally collinear manner at the blastoderm margin, where mesodermal and endodermal (mesendoderm) progenitor cells are specified and ingress to form mesendoderm/hypoblast. Functional analysis shows that these Hoxb genes regulate the timing of cell ingression: under- or overexpression of Hoxb genes perturb the timing of mesendoderm cell ingression and, consequently, the positioning of these cells along the forming anterior-posterior body axis after gastrulation. Finally, we found that Hoxb genes control the timing of mesendoderm ingression by regulating cellular bleb formation and cell surface fluctuations in the ingressing cells. Collectively, our findings suggest that Hoxb genes interconnect the temporal and spatial pattern of cell behaviors during zebrafish gastrulation by controlling cell surface fluctuations.

## Teaser

**Highlighted Article:** Timely cell ingression during zebrafish gastrulation requires Hoxb gene expression at blastoderm margin promoting cell surface fluctuations and associated membrane blebs.

## INTRODUCTION

The tight regulation of cell behaviors in time and space is essential for normal embryonic development. Gastrulation is the first morphogenetic process in embryonic development when dynamic and orchestrated cell behaviors lead to the formation of the three germ layers: ectoderm, mesoderm and endoderm. In zebrafish, gastrulation movements can be subdivided into three major processes (Heisenberg and Tada, 2002; Solnica-Krezel, 2005; Solnica-Krezel and Sepich, 2012; Warga and Kimmel, 1990). First, blastoderm cells spread over the yolk cell in a movement named ‘epiboly’. Second, mesendoderm progenitor cells at blastoderm margin internalize by synchronized cell ingression to form the mesendoderm cell layer (hypoblast) below the non-internalizing ectoderm progenitors (epiblast) (Carmany-Rampey and Schier, 2001; Giger and David, 2017). Third, cells within both the hypoblast and epiblast undergo convergence and extension movements leading to the formation and anterior-posterior elongation of the embryonic body axis. Synchronized mesendoderm cell internalization in zebrafish involves both cell-autonomous and non-cell-autonomous effects (Giger and David, 2017). We recently showed that mesendoderm progenitors can be subdivided into leader and follower cells with leader cells showing higher protrusive activity and cell-autonomously ingressing at the germ ring margin, while follower cells display lower protrusive activity and need to be pulled inside during ingression by the leader cells (Pinheiro et al., 2022).

Hox genes are transcription factors that play a pivotal role in determining anterior-posterior patterning in bilaterians (Pearson et al., 2005). They are arranged in chromosomal gene clusters and their positional order from 3′ to 5′ translates into their expression domains along the anterior-posterior body axis and the onset of their initial expression within the embryo, phenomena called ‘spatial collinearity’ (Duboule and Dollé, 1989; Graham et al., 1989) and ‘temporal collinearity’ (Deschamps et al., 1999; Duboule and Morata, 1994; Gaunt and Strachan, 1996; Izpisúa–Belmonte et al., 1991), respectively. Work on Hoxb genes in gastrulating chick embryos has shown that these two collinearities are tightly linked: Hoxb genes are expressed in a temporally ordered manner (temporal collinearity) and their expression in the epiblast lateral to the primitive streak regulates the timing of mesodermal cell ingression into the primitive streak, which again translates into their localization along the forming anterior-posterior body axis after the completion of gastrulation (Iimura and Pourquié, 2006). This suggests that the spatial collinearity of Hoxb genes is directly established by the temporal collinearity of their expression. In line with this, posterior Hox genes (paralogs 9-13) in tail-bud stage chick embryos are expressed in a temporally collinear manner within the presomitic mesoderm, progressively slowing down body axis elongation by repressing Wnt signaling (Denans et al., 2015). Similarly, Hox genes in *Xenopus* embryos are expressed in a temporally collinear manner during gastrulation, starting within the mesoderm and then expanding into the presumptive ectoderm. This led to the ‘time space translator model’, whereby the temporal collinear Hox expression within the mesoderm and its interaction with the embryonic organizer (Spemann organizer) defines positional information along the anterior-posterior axis in mesoderm and overlying neuroectoderm (Durston, 2018; Durston and Zhu, 2015; Durston et al., 2010a,b; Wacker et al., 2004). While these different studies suggest that Hox genes regulate the timing of cell involution/ingression/migration during gastrulation, which again results in the positioning of cells along the anterior-posterior body axis, the mechanism(s) by which Hox genes function in these processes are still insufficiently understood.

Cell blebbing is a characteristic cellular behavior, playing important roles in cell migration during development and disease. The formation of cellular blebs is triggered by the transient detachment of plasma membrane from the underlying actin cortex, leading to the formation of spherical membrane protrusions through intracellular pressure (Charras and Paluch, 2008; Paluch and Raz, 2013). In zebrafish, primordial germ cells use cell blebbing for their directional migration in response to chemotaxis utilizing SDF-1a (Cxcl12a)/CXCR4 ligand–receptor pairs (Blaser et al., 2006). Similar cell behaviors were observed in primordial germ cells in *Drosophila* embryos (Jaglarz and Howard, 1995) and human tumor cells that utilize cell blebbing for their directional migration (Guzman et al., 2020). Recently, cell blebbing and associated cell surface fluctuations have been implicated in cell sorting in early mouse embryos (Yanagida et al., 2022). However, the role of cell blebbing and cell surface fluctuations for cell movements during gastrulation is still not fully understood.

Here, we have examined the expression and function of Hoxb genes in zebrafish gastrulation. We found that Hoxb genes are expressed in a temporally collinear manner at the blastoderm margin during gastrulation, and that this collinear expression determines the timing of mesendoderm progenitor cell ingression and thus their localization along the anterior-posterior extent of the forming body axis. Hoxb genes appear to function in mesendoderm progenitor internalization by controlling their cell surface fluctuation and blebbing activity.

## RESULTS

### Hoxb gene expression at the blastoderm margin exhibits temporal collinearity during gastrulation

**Fig. 1.**
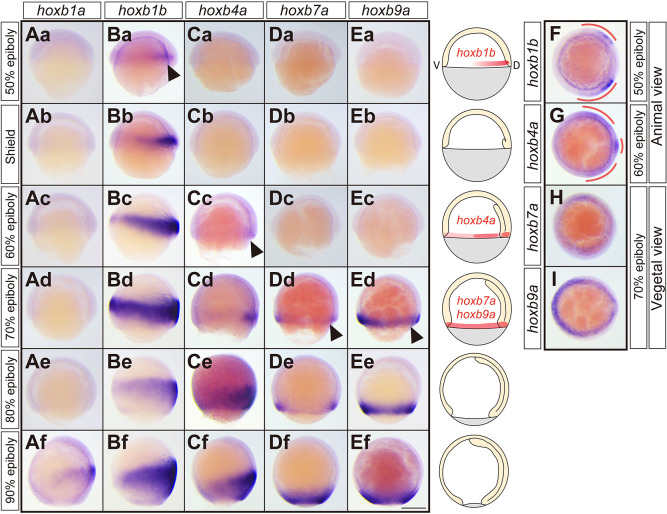
**Hoxb gene expression at the blastoderm margin exhibits temporal collinearity during gastrulation.** (Aa-Ef) Brightfield images (lateral views) of *hoxb1a* (Aa-Af), *hoxb1b* (Ba-Bf), *hoxb4a* (Ca-Cf), *hoxb7a* (Da-Df) and *hoxb9a* (Ea-Ef) expression patterns at 50% epiboly, shield, 60% epiboly, 70% epiboly, 80% epiboly and 90% epiboly stages. (F,G) Brightfield images (animal view) of *hoxb1b* (F) and *hoxb4a* (G) expression patterns at 50% and 60% epiboly, respectively. (H,I) Brightfield images (vegetal view) of *hoxb7a* (H) and *hoxb9a* (I) expression patterns at 70% epiboly. Scale bar: 200 µm. Schematics are shown for each of the developmental stages with red outlining the initial expression domains of *hoxb1b*, -*4a*, -*7a* and -*9a* at the blastoderm margin. Arrowheads indicate the initial expression of Hoxb genes at the blastoderm margin during gastrulation. Dorsal side is to the right.

To understand how Hox genes function in zebrafish gastrulation, we first examined the expression patterns of Hoxb genes during gastrulation. We focused on Hoxb genes as they had previously been shown to regulate the timing of cell ingression during chick gastrulation (Iimura and Pourquié, 2006). Amongst the Hoxb genes, we analyzed *hoxb1a*, *hoxb1b*, *hoxb4a*, *hoxb7a* and *hoxb9a* as representatives for anterior (*hoxb1a* and *hoxb1b*), middle (*hoxb4a*) and posterior (*hoxb7a* and *hoxb9a*) Hoxb genes, similar to what has been used previously (Iimura and Pourquié, 2006). While *hoxb1* has two paralogs, *hoxb1a* and *hoxb1b*, which might have been duplicated by a teleost-specific whole-genome duplication, *hoxb4*, *hoxb7* and *hoxb9* have only single paralogs. We found that although *hoxb1a* expression was largely absent during gastrulation until 90% epiboly stage (Fig. 1Aa-Af; Fig. S1E), *hoxb1b* expression was initiated at the dorsal blastoderm margin, except the dorsal most region, already at 50% epiboly stage (Fig. 1Ba,F; Fig. S1A) (Alexander et al., 1996). This expression increased and expanded along the animal-vegetal and dorsal-ventral axes in epiblast cells around the blastoderm margin at shield stage (Fig. 1Bb), eventually being expressed around the entire dorsal-ventral extent in both epiblast and hypoblast at 60-70% epiboly (Fig. 1Bc,Bd). *hoxb1b* continued to be expressed within both epiblast and hypoblast until the end of gastrulation with slightly lower expression within its ventral portion (Fig. 1Be,Bf; Fig. S1F). *hoxb4a* started to be expressed within the blastoderm margin only at 60% epiboly stage with the highest expression levels in the dorsal-most and dorsal-lateral regions of the margin (Fig. 1Ca-Cc,G; Fig. S1B). During subsequent stages of gastrulation, *hoxb4a* expression expanded along the animal-vegetal axis in both epiblast and hypoblast cells (Fig. 1Da-Dd,Ea-Ed,H,I; Fig. S1C,D), with the anterior (animal) limit of its expression domain being positioned more posterior (vegetal) than that of *hoxb1b* (Fig. 1Cf; Fig. S1G). Finally, *hoxb7a* and *hoxb9a* only started to be expressed at the blastoderm margin at 70% epiboly (Fig. 1Da-Dd,Ea-Ed; Fig. S1C,D) and their expression lasted in epiblast and hypoblast cells until the end of gastrulation (Fig. 1De,Df,Ee,Ef; Fig. S1H,I). Notably, the anterior limit of their expression domains was positioned more posteriorly than that of *hoxb4a* (Fig. 1Df,Ef). These spatiotemporal differences in the expression of Hoxb genes gave rise to their spatial collinear expression pattern detectable within the epiblast and hypoblast at 90% epiboly, and neural tube and somites at the 6-somite stage (Fig. 1Bf-Ef; Fig. S1J-N). Taken together, the expression patterns of Hoxb genes show temporal collinearity within the blastoderm margin during the course of gastrulation, leading to their spatial collinear expression within the trunk from late gastrulation onwards.

### Loss of function of ‘early’ Hoxb genes interferes with mesendoderm ingression and epiboly movements

**Fig. 2.**
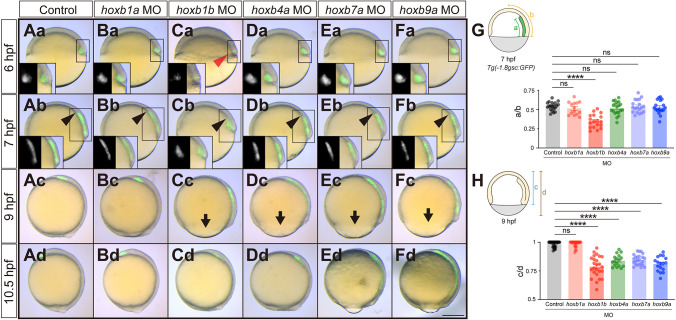
**Hoxb morphant embryos exhibit defective mesendodermal cell ingression/migration and epiboly movement delay.** (Aa-Fd) Overlay of brightfield and fluorescence images of control antisense morpholino oligonucleotide (MO) (Aa-Ad), *hoxb1a* MO (Ba-Bd), *hoxb1b* MO (Ca-Cd), *hoxb4a* MO (Da-Dd), *hoxb7a* MO (Ea-Ed) and *hoxb9a* MO (Fa-Fd) injected *Tg(-1.8gsc:GFP)* embryos at 6 hpf (shield stage in control embryos), 7 hpf (60% epiboly stage in control embryos), 9 hpf (90% epiboly stage in control embryos) and 10.5 hpf (bud stage in control embryos). Insets show separate brightfield and fluorescence images. Red arrowhead points at defective cell mesendoderm ingression at the dorsal blastoderm margin at 6 hpf. Black arrowheads point to the leading edge of mesendodermal cells migrating towards the animal pole. Arrows point to the blastoderm margin for the embryos exhibiting epiboly delay. All images are lateral view. Dorsal side is to the right. Scale bar: 200 µm. (G) Quantification of animal pole-oriented migration of mesendodermal cells [GFP-positive cells in *Tg(-1.8gsc:GFP)*] by the ratio of the length of the GFP-positive ingressed mesendoderm (a) to the distance from the blastoderm margin to the animal pole (b) in control MO (*n*=20, *N*=3), *hoxb1a* MO (*n*=14, *N*=3), *hoxb1b* MO (*n*=18, *N*=3), *hoxb4a* MO (*n*=20, *N*=3), *hoxb7a* MO (*n*=19, *N*=3) and *hoxb9a* MO (*n*=18, *N*=3) injected embryos at 7 hpf. ****P<0.0001 (one-way ANOVA). (H) Quantification of epiboly progression by the ratio of the blastoderm length (c) to the total embryo length from the animal pole to the vegetal pole (d) in control MO (*n*=26, *N*=3), *hoxb1a* MO (*n*=26, *N*=3), *hoxb1b* MO (*n*=25, *N*=3), *hoxb4a* MO (*n*=17, *N*=3), *hoxb7a* MO (*n*=24, *N*=3) and *hoxb9a* MO (*n*=17, *N*=3) injected embryos at 9 hpf. ****P<0.0001 (Kruskal–Wallis test). In G,H, data are shown as mean±s.e.m. ns, not significant (*P*>0.05).

Next, we examined the function of Hoxb genes during gastrulation. To this end, we first used morpholino oligonucleotides (MOs) to block the translation of Hoxb genes (knockdown; see Materials and Methods for target sequences). *hoxb1a* MO-injected embryos (*hoxb1a* morphants) showed no obvious defect during gastrulation compared to control MO-injected embryos, as expected from the absence of *hoxb1a* expression during gastrulation (Fig. 2Aa-Ad,Ba-Bd). *hoxb1b* morphants, in contrast, exhibited diminished mesendoderm ingression and/or animal pole-oriented migration at 6 h post-fertilization (hpf; shield stage) (Fig. 2Ca), as evidenced by migrating mesendoderm cells being positioned closer to the germ ring and further away from the animal pole in *hoxb1b* morphants compared to control MO-injected embryos at 7 hpf (60% epiboly; Fig. 2Cb). To quantify this phenotype, we utilized *Tg(-1.8gsc:GFP)* embryos to visualize ingressed mesendodermal cells. We measured the length of the GFP-positive ingressed mesendoderm and calculated the ratio relative to the distance from the blastoderm margin to the animal pole at 7 hpf. Our analysis revealed that the mesendoderm in *hoxb1b* morphants was significantly shorter than in control MO-injected embryos (Fig. 2G).

To determine whether this diminished translocation of mesendoderm cells applies to both mesoderm and endoderm cells, we analyzed endodermal cell positioning in *hoxb1b* morphants by visualizing the expression of the endodermal marker gene *sox17*. This showed that endoderm cell translocation towards the animal pole was reduced all around the germ ring in *hoxb1b* morphant embryos (Fig. S2A,B), suggesting that *hoxb1b* function is required for both mesoderm and endoderm cell ingression and/or animal pole-oriented migration. Defective mesendoderm translocation in *hoxb1b* morphants at the onset of gastrulation was followed by reduced epiboly movements of the blastoderm towards the vegetal pole at 9 hpf (90% epiboly in control embryos) and 10.5 hpf (bud stage in control embryos) (Fig. 2Cc,Cd). To quantify the epiboly phenotype, we measured the ratio of the blastoderm length to the total embryo length from the animal pole to the vegetal pole, as an indicator of epiboly progression. Our results showed that epiboly progression was significantly delayed in *hoxb1b* morphants at 9 hpf (Fig. 2Cc,H).

In contrast to *hoxb1b* morphants, *hoxb4a*, *hoxb7a* and *hoxb9a* morphants did not exhibit any obvious mesendoderm ingression or animal pole-oriented migration phenotype at 6 and 7 hpf (Fig. 2Da-Fa,Db-Fb,G), suggesting that they might be – at least partially – dispensable for these processes. Similar to *hoxb1b* morphants, however, *hoxb4a*, *hoxb7a* and *hoxb9a* morphants showed delayed blastoderm epiboly movements at the end of gastrulation (Fig. 2Dc-Fc,Dd-Fd,H), suggesting a common function of Hoxb genes in regulating blastoderm epiboly movements.

To test the specificity of the observed Hoxb morphant phenotypes, we also generated loss-of-function mutants for *hoxb1b*, *hoxb4a*, *hoxb7a* and *hoxb9a* genes by CRISPR/Cas9 (Fig. S2D; see Materials and Methods for target sequences). We then crossed mutants with *Tg(-1.8gsc:GFP)* and generated each Hoxb mutant line with *Tg(-1.8gsc:GFP)* backgrounds, and performed the same quantification on each Hoxb mutants as was done for MO-injected knockdown embryos. We found that *hoxb1b*, *hoxb4a*, *hoxb7a* and *hoxb9a* mutants embryos displayed phenotypes similar to their morphant counterparts (Fig. S2E-K), supporting the specificity of the observed morphant phenotypes. Next, we investigated whether the expression of Hoxb genes is altered when other Hoxb cluster genes are either compromised or overexpressed, given that cross-regulation among Hox genes has been reported in various contexts (Arcioni et al., 1992; Maconochie et al., 1997; Manzanares et al., 2001; Miller et al., 2001; Pöpperl et al., 1995; Studer et al., 1998). First, we examined the expression patterns of *hoxb4a*, *hoxb7a* and *hoxb9a* during gastrulation in *hoxb1b* mutant embryos. Expression was analyzed at 7, 7.6, 8.3 and 9 hpf, which correspond to approximately 50%, 60%, 80% and 90% epiboly progression, respectively. We found that *hoxb4a* initiates expression at the blastoderm margin at 7 hpf, while *hoxb7a* and *hoxb9a* begin expression at 7.6 hpf (Fig. S3A-D). These findings suggest that, under *hoxb1b* knockout conditions, *hox4a*, *hoxb7a* and *hoxb9a* expression is regulated in a time-dependent rather than an epiboly progression-dependent manner during gastrulation. Next, we examined the expression of *hoxb1b* during the initiation of gastrulation under *hoxb4a*, *hoxb7a* or *hoxb9a* knockout or overexpression conditions. We found that *hoxb1b* remained expressed at blastoderm margin, consistent with its expression in wild-type embryos. This indicates that neither loss nor gain of *hoxb4a*, *hoxb7a* or *hoxb9a* function affects *hoxb1b* transcription (Fig. S3E-J).

### Premature expression of ‘middle’ or ‘late’ Hoxb genes interferes with mesendoderm ingression and epiboly movements

**Fig. 3.**
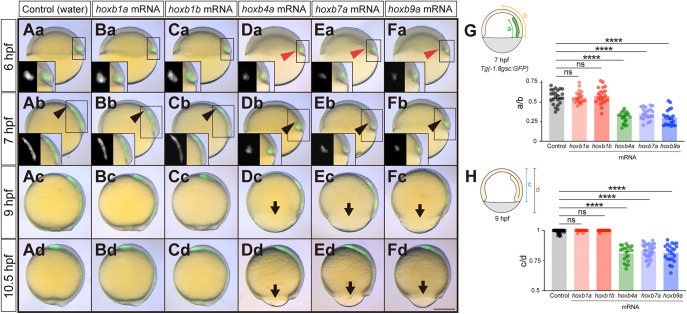
**‘Middle’ or ‘late’ Hoxb overexpressing embryos exhibit defective mesendodermal cell ingression/migration and epiboly movement delay.** (Aa-Fd) Overlay of brightfield and fluorescence images of control (water) (Aa-Ad), *hoxb1a* mRNA (Ba-Bd), *hoxb1b* mRNA (Ca-Cd), *hoxb4a* mRNA (Da-Dd), *hoxb7a* mRNA (Ea-Ed) and *hoxb9a* mRNA (Fa-Fd) injected *Tg(-1.8gsc:GFP)* embryos at 6 hpf (shield stage in control embryos), 7 hpf (60% epiboly stage in control embryos), 9 hpf (90% epiboly stage in control embryos) and 10.5 hpf (bud stage in control embryos). Insets show separate brightfield and fluorescence images. Red arrowheads point to defective mesendoderm ingression at dorsal blastoderm margin at 6 hpf. Black arrowheads point to the leading edge of mesendodermal cells migrating towards the animal pole. Arrows point to the blastoderm edge for the embryos exhibiting epiboly delay. All images are lateral view. Dorsal side is to the right. Scale bar: 200 µm. (G) Quantification of animal pole-oriented migration of mesendodermal cells [GFP-positive cells in *Tg(-1.8gsc:GFP)*] by the ratio of the length of the GFP-positive ingressed mesendoderm (a) to the distance from the blastoderm margin to the animal pole (b) in water (*n*=24, *N*=3), *hoxb1a* mRNA (*n*=22, *N*=3), *hoxb1b* mRNA (*n*=21, *N*=3), *hoxb4a* mRNA (*n*=22, *N*=3), *hoxb7a* mRNA (*n*=20, *N*=3) and *hoxb9a* mRNA (*n*=20, *N*=3) injected embryos at 7 hpf. ****P<0.0001 (one-way ANOVA). (H) Quantification of epiboly progression by the ratio of the blastoderm length (c) to the total embryo length from the animal pole to the vegetal pole (d) in water (*n*=34, *N*=3), *hoxb1a* mRNA (*n*=26, *N*=3), *hoxb1b* mRNA (*n*=31, *N*=3), *hoxb4a* mRNA (*n*=22, *N*=3), *hoxb7a* mRNA (*n*=29, *N*=3) and *hoxb9a* mRNA (*n*=22, *N*=3) injected embryos at 9 hpf. ****P<0.0001 (Kruskal–Wallis test). In G,H, data are shown as mean±s.e.m. ns, not significant (*P*>0.05).

To examine further the activity of Hoxb genes during gastrulation, we injected full-length mRNAs of each Hoxb gene and examined their overexpression phenotypes. We found that *hoxb1a* and *hoxb1b* mRNA-injected embryos exhibited no obvious phenotype during gastrulation compared to water-injected control embryos (Fig. 3Aa-Ad,Ba-Bd,Ca-Cd). *hoxb4a* mRNA-injected embryos, in contrast, showed diminished mesendoderm ingression and/or animal pole-oriented migration at 6 and 7 hpf (Fig. 3Da,Db,G), followed by delayed epiboly movements at 9 and 10.5 hpf (Fig. 3Dc,Dd,H). Similar phenotypes were also observed in embryos overexpressing *hoxb7a* or *hoxb9a* (Fig. 3Ea-Ed,Fa-Fd,G,H), suggesting that *hoxb4a*, *hoxb7a* or *hoxb9a*, upon premature and ectopic expression, exhibit comparable activity in suppressing mesendoderm ingression and/or animal pole-oriented migration during early gastrulation.

To determine whether the observed mesendoderm phenotypes in *hoxb4a-*, *hoxb7a-* or *hoxb9a*-overexpressing embryos were due to aberrant mesendoderm morphogenesis and not reduced mesendoderm specification, we analyzed the expression of the pan-mesendodermal marker gene *no tail* (*ntl*; *tbxta*) in *hoxb4a-*, *hoxb7a-* or *hoxb9a*-overexpressing embryos. We found that *ntl* expression levels at shield stage (6 hpf) did not show any major changes in *hoxb4a*, *hoxb7a* or *hoxb9a* mRNA-injected embryos compared to control embryos (Fig. S4A-E), suggesting that the observed mesendoderm defects in *hoxb4a-*, *hoxb7a-* or *hoxb9a*-overexpressing embryos are due to aberrant morphogenesis and not changes in cell fate specification. The phenotypes we observed upon Hoxb overexpression are consistent with the concept of ‘posterior prevalence’ (Duboule and Morata, 1994; Struhl, 1983), whereby premature overexpression of ‘late’ or ‘posterior’ Hoxb genes interfere with the function of ‘early’ or ‘anterior’ Hoxb genes, regulating early mesendoderm cell ingression.

We further investigated whether injection of *hoxb4a*, *hoxb7a* and *hoxb9a* mRNA could rescue the phenotypes in their respective mutant backgrounds. However, none of the mRNA injections successfully rescued the corresponding mutant phenotypes. Instead, mRNA-injected mutant embryos exhibited phenotypes similar to those observed in Hoxb overexpression wild-type embryos (Fig. S4F-N). These results suggest that the precise timing of Hoxb gene expression – rather than the expression level alone – is crucial for proper cell ingression and epiboly movements during gastrulation. Ectopic expression of mid- to late-acting Hoxb genes at the onset of gastrulation disrupts these developmental processes in *hoxb4a*, *hoxb7a* and *hoxb9a* mutant embryos.

### Hoxb genes determine the timing of mesendodermal cell ingression

**Fig. 4.**
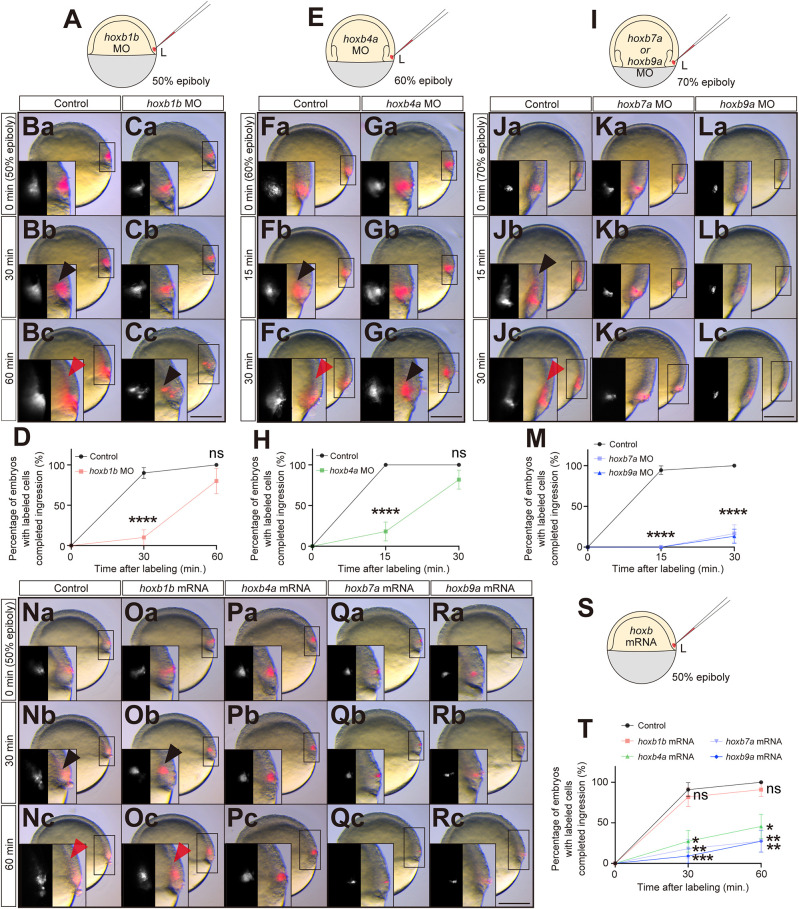
**Hoxb expression is required for proper mesendoderm morphogenesis.** (A) Schematic of DiI cell labeling at the lateral blastoderm margin at 50% epiboly for *hoxb1b* morphants. (Ba-Cc**)** Overlay of brightfield and fluorescence images of labeled cells in control (Ba-Bc) and *hoxb1b* morphants (Ca-Cc) at 0 min (50% epiboly, just after labeling), 30 min and 60 min after labeling. Scale bar: 200 µm. (D) Percentage of control (black; *n*=20, *N*=3) and *hoxb1b* morphant (red; *n*=10, *N*=3) embryos with all labeled cells having completed their ingression as a function of time after the labeling at 50% epiboly. ****P<0.0001 (Mann–Whitney test). (E) Schematic of DiI cell labeling at the lateral blastoderm margin at 60% epiboly for *hoxb4a* morphants. (Fa-Gc) Overlay of brightfield and fluorescence images of labeled cells in control (Fa-Fc) and *hoxb4a* morphants (Ga-Gc) at 0 min (60% epiboly, just after labeling), 15 min and 30 min after labeling. Scale bar: 200 µm. (H) Percentage of control (black; *n*=12, *N*=3) and *hoxb4a* morphant (green; *n*=11, *N*=3) embryos with all labeled cells having completed their ingression as a function of time after labeling at 60% epiboly. ****P<0.0001 (Mann–Whitney test). (I) Schematic of DiI cell labeling at the lateral blastoderm margin at 70% epiboly for *hoxb7a* or *hoxb9a* morphants. (Ja-Lc) Overlay of brightfield and fluorescence images of labeled cells in control (Ja-Jc), *hoxb7a* morphants (Ka-Kc) and *hoxb9a* morphants (La-Lc) at 0 min (70% epiboly, just after labeling), 15 min and 30 min after labeling. Scale bar: 200 µm. (M) Percentage of control (black; *n*=18, *N*=3) and *hoxb7a* (gray; *n*=12, *N*=3) and *hoxb9a* (blue; *n*=15, *N*=3) morphant embryos with all labeled cells having completed their ingression as a function of time after labeling at 70% epiboly. ****P<0.0001 (Kruskal–Wallis test). (Na-Rc) Overlay of brightfield and fluorescence images of labeled cells in control (Na-Nc), *hoxb1b* (Oa-Oc), *hoxb4a* (Pa-Pc), *hoxb7a* (Qa-Qc) and *hoxb9a* (Ra-Rc) mRNA-injected embryos at 0 min (50% epiboly, just after labeling), 30 min and 60 min after labeling. Scale bar: 200 µm. (S) Schematic of DiI cell labeling at the lateral blastoderm margin at 50% epiboly for Hoxb mRNA-injected embryos. (T) Percentage of control (black; *n*=11, *N*=3), *hoxb1b* (red; *n*=11, *N*=3), *hoxb4a* (green; *n*=11, *N*=4), *hoxb7a* (gray; *n*=11, *N*=4) or *hoxb9a* (blue; *n*=11, *N*=4) mRNA-injected embryos with all labeled cells having completed their ingression as a function of time after labeling at 50% epiboly. *P<0.05; **P<0.01; ***P<0.001 (Kruskal–Wallis test). Black arrowheads point to ingressed mesendodermal cells. Insets show magnified views of the boxed areas as separate brightfield and fluorescence images. Red arrowheads point to the leading edge of the ingressed mesendoderm. Ventral view with lateral (left side) to the right. *n* and *N* correspond to the number of embryos and independent experiments, respectively. In D,H,M,T, data are shown as mean±s.e.m. ns, not significant (*P*>0.05).

Hox genes have previously been shown to determine the timing of mesoderm cell ingression during chick gastrulation (Iimura and Pourquié, 2006). To investigate whether Hoxb genes might have a similar function in zebrafish gastrulation, we analyzed mesendoderm ingression in Hoxb loss or gain-of-function embryos. To this end, we labeled cells within the lateral blastoderm margin with fluorescent tracer DiI at the stages when each Hoxb gene is initially expressed and traced the labeled cell populations in control and Hoxb loss-of-function embryos (Fig. 4A,E,I). In nearly all control embryos at 50% epiboly stage, when endogenous *hoxb1b* initiates its expression (Fig. 1Ba), mesendoderm progenitors labeled at 50% epiboly had completed their migration into the hypoblast 30 min after their labeling. In contrast, in only 10% of the *hoxb1b* morphant embryos, the labeled mesendoderm cells had ingressed 30 min after their labeling, and even after 60 min only 80% of the morphant embryos showed complete ingression of labeled cells (Fig. 4A,Ba-Bc,Ca-Cc,D). This suggests that *hoxb1b* is required for timely ingression of mesendoderm cells at shield stage.

In *hoxb4a* morphants at 60% epiboly, corresponding to the stage when *hoxb4*a is initially expressed (Fig. 1Cc), ingression of cells labeled at 60% epiboly was severely reduced with only in 18% of the labeled embryos showing complete cell ingression 15 min after the labeling, a stage when labeled cell ingression was completed in all of the control embryos (Fig. 4E,Fa-Fc,Ga-Gc,H). Finally, *hoxb7a* or *hoxb9a* morphants at 70% epiboly, when these genes begin to be expressed within the blastoderm margin (Fig. 1Dd,Ed), also showed strongly reduced ingression of cells labeled at 70% epiboly, resulting in only 16% and 10% of the morphant embryos, respectively, showing complete cell ingression 30 min after labeling, when labeled cell ingression was completed in all control embryos (Fig. 4I,Ja-Jc,Ka-Kc,La-Lc,M). These results indicate that Hoxb genes are required for timely ingression of mesendoderm cells at the time point of their initial expression.

To test whether Hoxb gene expression is also sufficient to control the timing of mesendoderm cell ingression, we analyzed cell ingression at shield stage (6 hpf) in embryos overexpressing different Hoxb genes (Fig. 4S). Although overexpression of *hoxb1b* had no major effect on labeled mesendoderm cell ingression at shield stage (Fig. 4Na-Nc,Oa-Oc,T), embryos overexpressing *hoxb4a*, *hoxb7a* or *hoxb9a* genes exhibited clearly reduced cell ingression at shield stage (Fig. 4Pa-Pc,Qa-Qc,Ra-Rc,T). This suggests that ectopic and premature expression of ‘late’ Hoxb genes (*hoxb4a*, *hoxb7a* and *hoxb9a*) is sufficient to interfere with mesendoderm cell ingression at the onset of gastrulation, an effect consistent with the concept of ‘posterior prevalence’ (Duboule and Morata, 1994; Struhl, 1983).

**Fig. 5.**
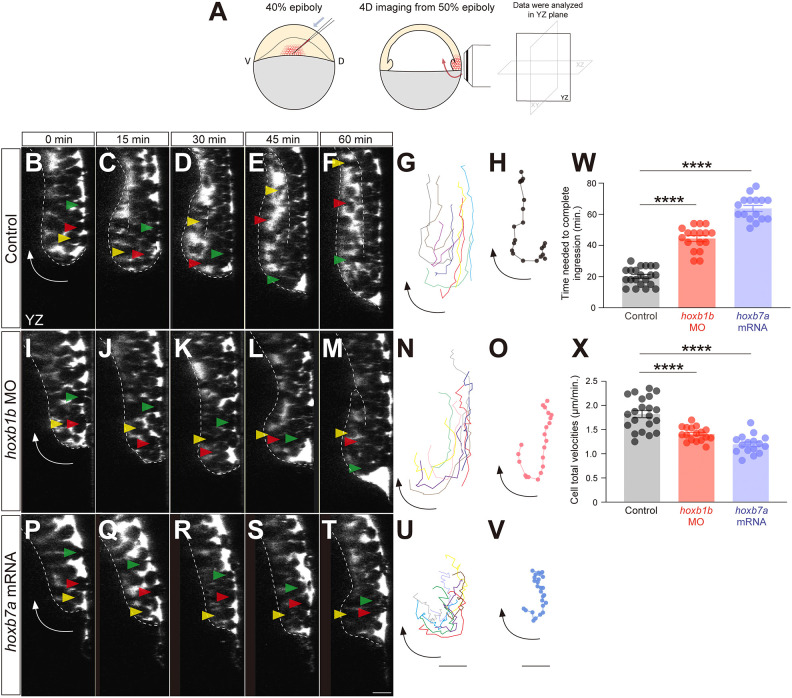
**Hoxb expression determines the timing of mesendodermal cell ingression.** (A) Schematic of mesendodermal cell movement analysis. D, dorsal; V, ventral. (B-F,I-M,P-T) Confocal fluorescence images of the lateral blastoderm margin at consecutive stages of mesendodermal cell ingression from 50% epiboly stage (0 min) onwards in control (B-F), *hoxb1b* morphant (I-M) and *hoxb7a*-overexpressing (P-T) embryos. Arrows depict the overall movement direction of cells at the blastoderm margin. Yellow, red and green arrowheads depict individual mesendodermal cells during ingression. Scale bar: 20 µm. (G,N,U) Representative tracks of mesendodermal cells undergoing ingression in control (G), *hoxb1b* morphant (N) and *hoxb7a*-overexpressing (U) embryos. Eight cells were tracked in each condition. Cells were tracked for 0-60 min (3 min/frame) in control embryos and *hoxb1b* morphants, and for 0-75 min (3 min/frame) in *hoxb7a*-overexpressing embryos. Scale bar: 20 µm. (H,O,V) Representative single-cell track of a mesendodermal cell undergoing ingression at the blastoderm margin at 50% epiboly in control (H), *hoxb1b* morphant (O) and *hoxb7a*-overexpressing (V) embryos. Dots represents each time point. Scale bar: 20 µm. (W) Average time needed for mesendodermal cells to complete ingression from 50% epiboly onwards in control (*n*=21, *N*=3), *hoxb1b* morphant (*n*=17, *N*=3) and *hoxb7a*-overexpressing (*n*=16, *N*=3) embryos. ****P<0.0001 (one-way ANOVA). (X) Average cell total velocities of mesendodermal cells undergoing ingression in control (*n*=21, *N*=3), *hoxb1b* morphant (*n*=17, *N*=3) and *hoxb7a*-overexpressing (*n*=16, *N*=3) embryos. ****P<0.0001 (one-way ANOVA). In W,X, data are shown as mean±s.e.m. *n* and *N* correspond to the number of cells and independent experiments, respectively.

To analyze which aspects of mesendoderm cell ingression are altered by Hoxb over- or underexpression, we traced cell ingression in control, *hoxb1b* morphant and *hoxb7a*-overexpressing embryos at the onset of gastrulation (6 hpf; Fig. 2Ca; Fig. 3Ea). For visualizing and tracing cells in three dimensions over time (4D), we injected Dextran Mini-Ruby into the intercellular space and recorded mesendoderm cell ingression within the lateral germ ring (Fig. 5A; described as ‘0 min’ in Fig. 5B,I,P). In control embryos, cells first moved vegetally towards the germ ring margin, followed by ingression towards the yolk cell and migration away from the germ ring margin towards the animal pole of the gastrula (Fig. 5B-F). Consistent with our previous observations (Pinheiro et al., 2022), we found that cells right at the germ ring margin were the first to undergo ingression and migration away from the margin towards the animal pole, sequentially followed by cells positioned further away from the margin (Fig. 5B-H). In *hoxb1b* morphant and *hoxb7a-*overexpressing embryos, mesendoderm cell ingression occurred in a similar pattern as in control embryos. However, the onset of cell ingression was delayed (Fig. 5I-W) and the velocity of cell movement during ingression was significantly reduced (Fig. 5X; Movie 1), suggesting that the spatiotemporally ordered expression of Hoxb genes is required for timely and efficient mesendoderm cell ingression at the onset of gastrulation.

### Hoxb genes regulate mesendodermal cell ingression timing at the blastoderm margin in a cell-autonomous manner

**Fig. 6.**
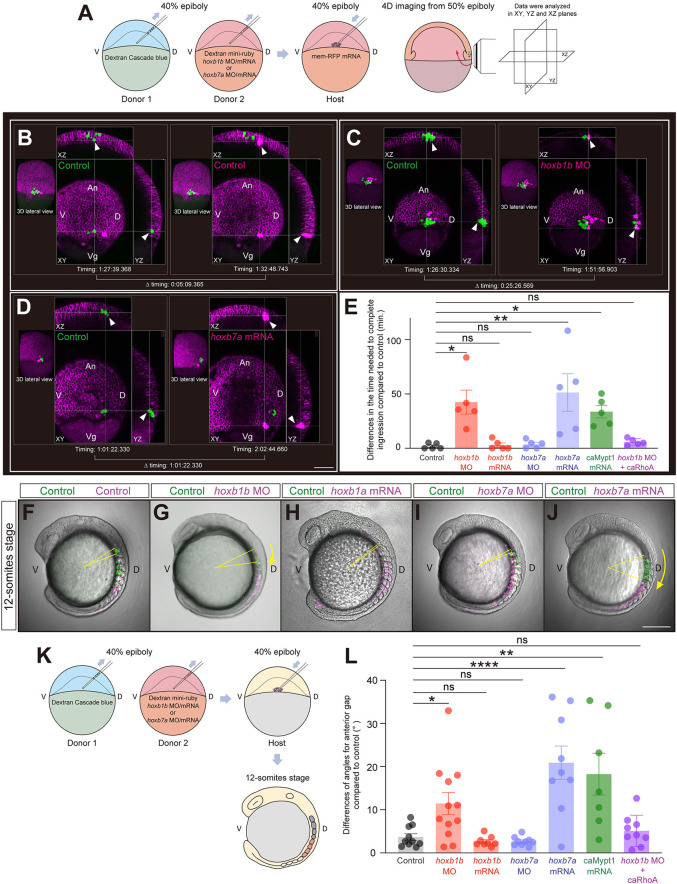
**Hoxb expression cell-autonomously determines the timing of mesendodermal cell ingression and their resulting position along the anterior-posterior body axis.** (A) Schematic of the cell transplantation assay and subsequent 4D imaging. (B-D) Representative images demonstrating the time needed for the transplanted cells to complete ingression in host embryos containing a combination of transplanted control cells (green; left) with control (B), *hoxb1b* MO-injected (C) or *hoxb7a* mRNA-injected (D) cells (magenta; right). Arrowheads point to ingressing mesendodermal cells. The timing is indicated as h:min:s. Scale bar: 100 µm. (E) Average difference in the time needed for transplanted control cells and co-transplanted control (*N*=5), *hoxb1b* MO (*N*=5), *hoxb1b* mRNA (*N*=5), *hoxb7a* MO (*N*=5), *hoxb7a* mRNA (*N*=5), caMypt1 mRNA (*N*=5) or *hoxb1b* MO plus caRhoA mRNA (*N*=5) injected cells to complete ingression. *P<0.05; **P<0.01 (one-way ANOVA). (F-J) Localization of transplanted cells along the anterior-posterior body axis within the somitic mesoderm of host embryos at the 12-somite stage for control (green) co-transplanted with control (F), *hoxb1b* MO (G), *hoxb1* mRNA (H), *hoxb7a* MO (I) or *hoxb7a* mRNA (J) injected cells (magenta) at 40% epiboly stage. Yellow arrows outline the angles between the most anteriorly located control cells (green) and co-transplanted control, *hoxb1b* MO/mRNA or *hoxb7a* MO/mRNA cells (magenta). Scale bar: 200 µm. (K) Schematic of double transplantation assay for determining distribution patterns of transplanted cells in 12-somite stage embryos. (L) Average angles between the most anteriorly located control cells and co-transplanted control (*n*=10, *N*=3), *hoxb1b* MO (*n*=12, *N*=3), *hoxb1b* mRNA (*n*=8, *N*=3), *hoxb7a* MO (*n*=9, *N*=3), *hoxb7a* mRNA (*n*=9, *N*=3), caMypt1 mRNA (*n*=7, *N*=2) or *hoxb1b* MO plus caRhoA mRNA (*n*=9, *N*=2) injected cells within the somitic mesoderm of host embryos at the 12-somite stage. *P<0.05; **P<0.01; ****P<0.0001 (one-way ANOVA). *n* and *N* correspond to the number of embryos and independent experiments, respectively. In E,L, data are shown as mean±s.e.m. ns, not significant (*P*>0.05). An, animal pole; D, dorsal; V, ventral; Vg, vegetal pole.

To examine how Hoxb genes function in mesendoderm cell ingression, we transplanted ∼10-20 control and *hoxb1b* or *hoxb7a* morphant or overexpressing cells from the lateral germ ring margin of donor embryos into the corresponding region of a control host embryo at 40% epiboly stage. We then traced the ingression behavior of the transplanted cells in 4D and determined the timing of cell ingression for each of the transplanted cells (Fig. 6A). We defined cells as ‘ingressed’ when they had entered the hypoblast tissue at the blastoderm margin. When only control cells were transplanted, the transplanted cells ingressed in close succession with an average time difference of ∼2 min (Fig. 6B,E; Movie 2). In contrast, when control cells were co-transplanted with *hoxb1b* morphant or *hoxb7a*-overexpressing cells, ingression of the morphant/overexpressing cells was delayed by ∼52 min compared to control cells (Fig. 6C-E; Movies 3 and 4). Such delay was not observed when control cells were co-transplanted with *hoxb1b*-overexpressing or *hoxb7a* morphant cells (Fig. 6E; Fig. S5A,B). This suggests that interfering with the expression of ‘early’ Hoxb genes (*hoxb1b*) or premature expression of ‘late’ Hoxb genes (*hoxb7a*) leads to a cell-autonomous delay in cell ingression at shield stage.

### Hoxb-mediated timing of mesendodermal cell ingression during gastrulation translates into their localization along anterior-posterior body axis post gastrulation

To determine how the timing of mesendoderm cell ingression translates into the positioning of the ingressed cells along the forming body axis, we performed the same transplantation assay as described above and analyzed the distribution pattern of the transplanted cells at the 12-somite stage (after completion of gastrulation) (Fig. 6K). We found that delayed ingression of *hoxb1b* morphant or *hoxb7a*-overexpressing cells led to these cells being positioned more posteriorly along the body axis than the co-transplanted control cells (Fig. 6F,G,J,L). No such more posterior positioning was found when control cells were co-transplanted with *hoxb1b*-overexpressing or *hoxb7a* morphant cells (Fig. 6H,I,L). Taken together, these results suggest that Hoxb genes determine the proper timing for mesendoderm cell ingression, and that the timing of cell ingression determines the positioning of the ingressed cells along the anterior-posterior axis of the forming body axis.

### Initiation of frequent mesendodermal cell blebbing at the blastoderm margin, regulated by Hoxb gene expression, is required for cell ingression

To obtain some initial insight into the mechanisms by which Hoxb genes determine the timing of mesendoderm cell ingression, we analyzed the protrusive behavior of ingressing cells, which we previously showed to mediate their cell ingression behavior (Pinheiro et al., 2022). We reasoned that Hoxb genes might control the timing of cell ingression by promoting the protrusive activity of mesendoderm cells. To challenge this possibility, we performed the same transplantation assay as described above and visualized the protrusive activity of the transplanted mesendoderm cells. We found that the number of F-actin-filled protrusions toward yolk syncytial layer formed by control and *hoxb1b* morphant or *hoxb7a*-overexpressing cells was not significantly different (Fig. S6A-E), suggesting that the formation of actin-rich cell protrusions as such is not regulated by Hoxb genes.

**Fig. 7.**
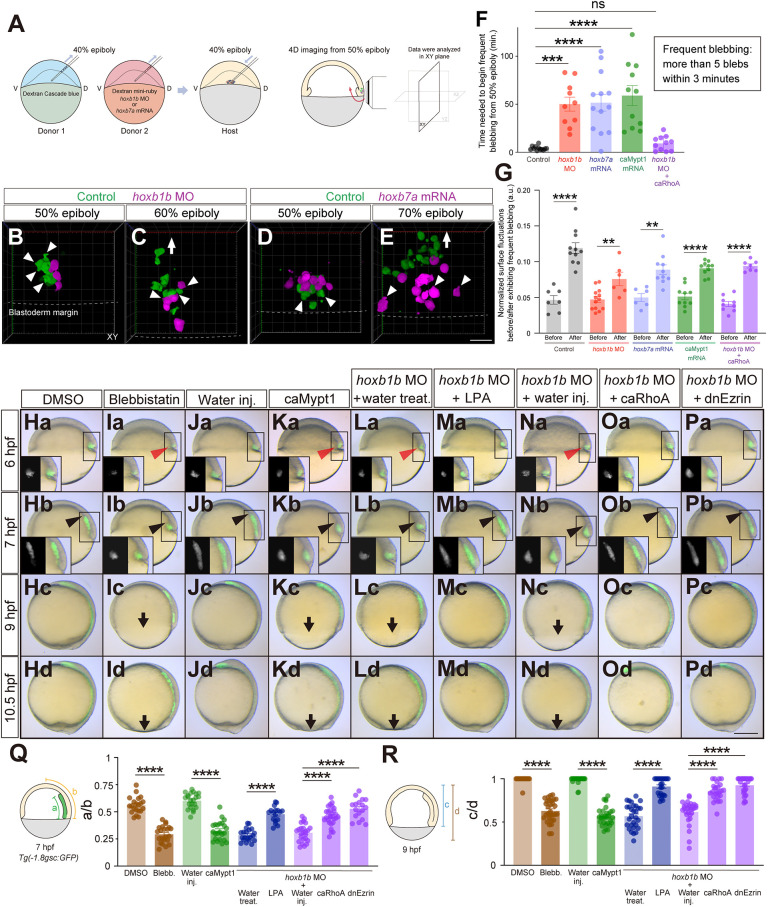
**Hoxb expression affects mesendodermal cell blebbing and associated cell surface fluctuations.** (A) Schematic of the cell transplantation assay and subsequent 4D imaging. D, dorsal; V, ventral. (B-E) Confocal fluorescence images (3D projection) of transplanted control (green) and *hoxb1b* MO (B,C) or *hoxb7a* mRNA (D,E) injected cells (magenta) at 50% epiboly (B,D) and 60% (C) or 70% (E) epiboly. Arrowheads point to cellular blebs. Arrow demarcates the migration direction of ingressed mesendodermal cells towards the animal pole. Dashed lines depict the borders between blastoderm margin and yolk. Scale bar: 60 µm. (F) Time needed for mesendodermal cells to begin frequent blebbing after the onset of imaging in control (*n*=12, *N*=4), *hoxb1b* MO (*n*=10, *N*=3), *hoxb7a* mRNA (*n*=13, *N*=4), caMypt1 mRNA (*n*=11, *N*=3) and *hoxb1b* MO plus caRhoA mRNA (*n*=11, *N*=4) injected cells. Frequent blebbing is defined as the state when a cell shows more than five blebs within 3 min. ***P<0.001, ****P<0.0001 (one-way ANOVA). (G) Normalized surface fluctuations of control (*n*=7, *N*=3 before; *n*=11, *N*=3 after), *hoxb1b* MO (*n*=13, *N*=3 before; *n*=6, *N*=3 after), *hoxb7a* mRNA (*n*=6, *N*=3 before; *n*=10, *N*=3 after), caMypt1 mRNA (*n*=10, *N*=3 before; *n*=10, *N*=3 after) and *hoxb1b* MO plus caRhoA mRNA (*n*=9, *N*=3 before; *n*=7, *N*=3 after) injected cells in the states before or after exhibiting frequent blebbing. **P<0.01; ****P<0.0001 (Mann–Whitney test). (Ha-Pd) Overlay of brightfield and fluorescence images of DMSO-treated (Ha-Hd), blebbistatin-treated (Ia-Id), water-injected (Ja-Jd), caMypt1 mRNA-injected (Ka-Kd) embryos, and water-treated (La-Ld), LPA-treated (Ma-Md), water-injected (Na-Nd) and caRhoA mRNA-injected (Oa-Od) and dnEzrin mRNA-injected (Pa-Pd) *hoxb1b* morphant embryos at 6 hfp, 7 hpf, 9 hpf and 10.5 hpf. Red arrowheads point to defective mesendodermal cell ingression at the dorsal blastoderm margin at 6 hpf. Black arrowheads point to the leading edge of mesendodermal cells migrating towards the animal pole. Arrows point to the blastoderm margin at 9 hpf. Dorsal side is to the right. Insets show separate brightfield and fluorescence images. Scale bar: 200 µm. (Q) Quantification of animal pole-oriented migration of mesendodermal cells [GFP-positive cells in *Tg(-1.8gsc:GFP)*] by the ratio of the length of the GFP-positive ingressed mesendoderm (a) to the distance from the blastoderm margin to the animal pole (b) in DMSO-treated (*n*=21, *N*=3), blebbistatin-treated (*n*=22, *N*=3), water-injected (*n*=22, *N*=3), caMypt1 mRNA-injected (*n*=23, *N*=3) embryos, and water-treated (*n*=20, *N*=3), LPA-treated (*n*=17, *N*=3), water-injected (*n*=24, *N*=3), caRhoA mRNA-injected (*n*=28, *N*=3) and dnEzrin mRNA-injected (*n*=17, *N*=3) *hoxb1b* morphant embryos at 7 hpf. ****P<0.0001 (one-way ANOVA). (R) Quantification of epiboly progression by the ratio of the blastoderm length (c) to the total embryo length from the animal pole to the vegetal pole (d) in DMSO-treated (*n*=17, *N*=3), blebbistatin-treated (*n*=30, *N*=3), water-injected (*n*=28, *N*=3), caMypt1 mRNA-injected (*n*=31, *N*=3) embryos, and water-treated (*n*=29, *N*=3), LPA-treated (*n*=24, *N*=3), water-injected (*n*=30, *N*=3), caRhoA mRNA-injected (*n*=25, *N*=3) and dnEzrin mRNA-injected (*n*=31, *N*=3) *hoxb1b* morphant embryos at 9 hpf. ****P<0.0001 (Kruskal–Wallis test). In F,G,Q,R, data are shown as mean±s.e.m. ns, not significant (*P*>0.05).

Given that cell blebbing behavior has previously been observed within the blastoderm margin during zebrafish gastrulation (Row et al., 2011) and recent studies have shown that cell blebbing and surface fluctuations play pivotal roles for segregation of epiblast and primitive endoderm in early mammalian embryos (Yanagida et al., 2022), we examined whether Hoxb genes might determine the timing of mesendoderm cell ingression by upregulating blebbing/surface fluctuations of mesendoderm cells. To address this possibility, we used the same transplantation assay as described above and examined the frequency of bleb formation in the transplanted mesendoderm cells (Fig. 7A). When control cells were co-transplanted with *hoxb1b* morphant or *hoxb7a*-overexpressing cells, control cells started to form frequent blebs shortly before initiating ingression; *hoxb1b* morphant or *hoxb7a*-overexpressing cells, in contrast, started to form blebs later than the control cells (Fig. 7B-E; Movie 5-8; ‘frequent blebbing’ was defined as the state when cells form more than five blebs within 3 min). We found that *hoxb1b* morphant and *hoxb7a*-overexpressing cells exhibited frequent blebs significantly later than the control cells (Fig. 7F). To test whether Hoxb-dependent mesendoderm cell blebbing is an expression of increased cell surface fluctuations in mesendoderm cells, we quantified the dynamics of cell surface fluctuations in cells within the germ ring margin before versus after exhibiting frequent blebs (Fig. S6F-I). We found that cells exhibiting frequent blebbing directly preceding their ingression showed significantly larger cell surface fluctuations than non-ingressing cells exhibiting less blebbing (Fig. 7G). This suggests that Hoxb genes determine the timing of mesendoderm cell ingression by upregulating surface fluctuations in these cells.

Next, to test whether increased cell blebbing and surface fluctuations constitute the mechanism by which Hoxb genes regulate the timing of mesendoderm cell ingression, we tested whether interfering with cell blebbing affects timely cell ingression. When we treated embryos with 10 μM blebbistatin, an inhibitor of non-muscle myosin II reducing bleb formation, from 30% to 40% epiboly, we found that blebbistatin-treated embryos, similar to *hoxb1b* morphant embryos, displayed delayed mesendoderm ingression and animal pole-oriented migration at shield stage, and slower epiboly movements while DMSO-treated control embryos did not (Fig. 7Ha-Hd,Ia-Id,Q,R; Fig. 2Ca-Cd). Similarly, when we injected 75 pg of constitutively active *mypt1* (*ppp1r12a*) (caMypt1) mRNA – previously shown to reduce actomyosin contractility (Schwayer et al., 2019; Smutny et al., 2017) – the resulting embryos exhibited delayed mesendoderm ingression and impaired animal pole-oriented migration. These phenotypes closely resembled those observed in *hoxb1b* morphants and blebbistatin-treated embryos, whereas water-injected control embryos did not display such defects (Fig. 7Ja-Jd,Ka-Kd,Q,R; Fig. 2Ca-Cd).

To test whether increasing cell blebbing and surface fluctuations can also rescue the mesendoderm cell ingression phenotype in *hoxb1b* morphant embryos, we increased cell blebbing in *hoxb1b* morphants by treating them with either lysophosphatidic acid (LPA), overexpressing a constitutively active version of RhoA (caRhoA), or dominant-negative version of Ezrin (dnEzrin), all of which have previously been shown to increase actomyosin contractility and cell blebbing in zebrafish embryos (Diz-Muñoz et al., 2010; Ruprecht et al., 2015; Takesono et al., 2012) *hoxb1b* morphants treated with 10 μM LPA, injected with 1 pg caRhoA mRNA or 250 pg dnEzrin mRNA exhibited normal mesendoderm cell ingression and epiboly movements while water-treated or water-injected embryos did not (Fig. 7La-Ld,Ma-Md,Na-Nd,Oa-Od,Pa-Pd; Fig. 2Aa-Ad). Notably, this rescue of cell movements in caRhoA mRNA-injected *hoxb1b* morphant embryos phenotype was accompanied by normalized mesendoderm cell blebbing behavior, surface fluctuations, ingression timing, and distribution pattern along the anteroposterior axis at somite stage (Fig. 6E,L; Fig. 7F,G,Oa-Od; Fig. S5C,D; Movie 9), further supporting the notion that Hoxb genes control the timing of mesendoderm cell ingression by regulating cell blebbing and surface fluctuations.

To investigate whether cell blebbing is a crucial mechanism for cell ingression downstream of mid- and late-acting Hoxb genes – such as *hoxb4a*, *hoxb7a* and *hoxb9a* – we treated mutant embryos with 10 μM LPA at the shield stage. We found that LPA treatment rescued both cell ingression and epiboly phenotypes in *hoxb4a*, *hoxb7a* and *hoxb9a* mutants (Fig. S7A-H). These results suggest that, similar to early Hoxb genes, mid- and late-expressed Hoxb genes also regulate cell ingression and epiboly movements via mechanisms involving cell blebbing.

Finally, we compared cell dynamics during ingression between the lateral and dorsal germ ring margins. Our current study shows that cells in the lateral blastoderm margin ingress into hypoblast primarily through blebbing, characterized by increased cell surface fluctuations triggered by Hoxb gene activity. In contrast, our previous work demonstrated that cells in the dorsal blastoderm margin undergo protrusion-driven ingression (Pinheiro et al., 2022). To compare these dynamics directly, we performed cell transplantation followed by 4D imaging of the lateral and dorsal blastoderm margins (Fig. S7I). We observed that dorsal transplanted cells exhibited significantly more protrusions and fewer blebs during ingression compared to lateral transplanted cells (Fig. S7J-L). After ingression into the hypoblast, dorsal cells continued to display a higher frequency of both protrusions and blebs than lateral cells (Fig. S7M-O). Importantly, these dynamic behaviors of dorsal transplanted cells were not altered by *hoxb1b* MO injection (Fig. S7J,K,M,N). These findings suggest that the blebbing- and surface fluctuation-driven cell ingression mechanism is specific to the lateral blastoderm margin, where *hoxb1b* is expressed, and is distinct from the protrusion-based mechanism in the dorsal margin. Furthermore, considering the axial mesendoderm ingression defects observed in *hoxb1b* knockdown or knockout embryos, it is plausible that the proper dynamics of a *hoxb1b*-expressing cell population in the lateral blastoderm margin are essential for enabling effective cell ingression and subsequent animal pole-oriented migration in the dorsal region.

## DISCUSSION

Our study demonstrates temporal collinearity of Hoxb gene expression during zebrafish gastrulation (Fig. S8). In line with this finding are previous reports on the temporal expression pattern of other Hox genes, such as *hoxc4a* (Hayward et al., 2015), *hoxb6b*, *hoxc6a* and *hoxc6b* (Hayward et al., 2015; Thisse and Thisse, 2004, 2005), *hoxc8a* (Hayward et al., 2015; Thisse and Thisse, 2004) and *hoxa9a*, *hoxb10a* and *hoxc10a* during zebrafish epiboly (Hayward et al., 2015; Thisse and Thisse, 2004), showing that ‘anterior’ Hox genes are expressed earlier than ‘posterior’ ones. Furthermore, Hox genes in other species, such as mouse (Deschamps et al., 1999; Forlani et al., 2003), chicken (Gaunt and Strachan, 1996; Gouveia et al., 2015; Iimura and Pourquié, 2006; Lemaire and Kessel, 1997; Moreau et al., 2019), *Xenopus* (Wacker et al., 2004) and cnidarians (DuBuc et al., 2018), have also been shown to exhibit temporal collinearity in their expression during gastrulation, demonstrating that temporal collinearity is a widespread feature of Hox gene expression. Notably, however, in acoels (Hejnol and Martindale, 2009) and annelids (Kulakova et al., 2007), and most recently also in *Xenopus tropicalis* (Kondo et al., 2019), Hox genes do not show temporal collinear expression, suggesting that temporal collinearity is not universally conserved in animal evolution.

Importantly, our study goes beyond these previous observations by providing insight into the cell-autonomous and non-cell-autonomous processes by which Hox genes regulate mesendoderm cell ingression. In particular, our finding that underexpression of ‘early’ Hoxb genes and overexpression of ‘late’ ones delay cell ingression in a cell-autonomous manner, suggests that Hox genes determine cell ingression in a cell-autonomous manner (Carmany-Rampey and Schier, 2001; David and Rosa, 2001). Yet mesendodermal cell ingression has also been shown to display non-cell-autonomous features with ingression-competent cells taking along incompetent cells during ingression (Carmany-Rampey and Schier, 2001) and endoderm internalization being achieved by the collective internalization of mesodermal cells (Giger and David, 2017). Furthermore, our own recent findings suggest that axial ingressing mesendoderm cells can be subdivided into ‘leader’ cells, showing high protrusive activity and being able to cell-autonomously ingress at the onset of gastrulation, and ‘follower’ cells that are less protrusive and pulled inside by leader cells during ingression (Pinheiro et al., 2022). Our present findings of early Hoxb gene expression initiating the onset of mesendoderm ingression suggest that early mesendoderm ingression is a cell-autonomous process dependent on the timely expression of ‘early’ Hox genes, such as *hoxb1b*.

Interestingly, our data also show that the premature expression of ‘late’ Hoxb genes, such as *hoxb7a*, suppresses early mesendoderm cell ingression, suggesting that ‘late’ Hoxb genes cannot trigger early mesendoderm ingression. How different Hox genes control mesendoderm cell ingression in a stage-dependent manner is not yet clear, but it is conceivable that different Hox genes elicit distinct cell behaviors required for cell ingression at different stages of gastrulation. Our finding that cells shortly before ingression display enhanced blebbing and surface fluctuations, point to the possibility that Hox genes determine the timing of mesendodermal cell ingression by promoting cell surface fluctuations. In line with this, it has been reported that Hox proteins regulate *Epha4* and Eph/ephrin signaling in chick and mouse (Prin et al., 2014), and that EphA4 in turn regulates actomyosin contractility in zebrafish (Cayuso et al., 2019). These findings suggest that Hox genes may modulate actomyosin contractility – and the associated cell blebbing and surface fluctuations – through the regulation of *Epha4* and Eph/ephrin signaling pathways.

A previous study showed that cell surface fluctuations play a pivotal role for segregation of epiblast and primitive endoderm in early mammalian embryos (Yanagida et al., 2022). Given that cell surface fluctuations have been linked to tissue fluidity (Yanagida et al., 2022), and that actively ingressing mesendoderm progenitor cells have been proposed to undergo motility-driven unjamming (Pinheiro et al., 2022), it is conceivable that Hoxb genes drive mesendoderm cell ingression by triggering their motility-driven unjamming. Yet how the induction of such general behavior is modulated so that different Hox genes can exert their function in a stage-specific manner remains to be investigated. Furthermore, motility-driven unjamming and its associated cell ingression behavior has been reported at the dorsal blastoderm margin during zebrafish gastrulation (Pinheiro et al., 2022), whereas our current study focuses on the lateral region. Indeed, we also found that cells in the dorsal or lateral blastoderm margin ingress into the hypoblast, exhibiting distinct cell dynamics (Fig. S7I-O). This difference in observation sites and cell dynamics raises the possibility of distinct mechanisms behind the orchestrated cell ingression.

Our study suggests that Hoxb genes determine mesendoderm cell ingression timing by upregulating RhoA/Rock-mediated cortical contractility and cell blebbing. This is different from chicken gastrulation where mesendoderm cell ingression is initiated by basement membrane breakdown, which again is controlled by a loss of basally localized RhoA activity (Nakaya et al., 2008). Furthermore, blocking RhoA activity can rescue the delay in mesendoderm cell ingression in chicken embryos overexpressing *Hoxa13* (Denans et al., 2015), suggesting that Hox genes determine the timing of mesendoderm cell ingression in chicken gastrulation by downregulating RhoA activity, rather than upregulating it as suggested by our present findings in zebrafish. How the molecular and cellular mechanisms by which Hox genes determine the timing of mesendoderm ingression have been adapted to the specific organismal context during evolution remains an intriguing question for future research.

## MATERIALS AND METHODS

### Zebrafish lines and husbandry

Fish maintaining, injection and staging were conducted according to established procedures (Westerfield, 2007). AB or RW strains were used as wild-type control embryos. Embryos were obtained through natural spawning, kept in E3 medium at 28.5-31°C prior to experiments, and staged based on morphological criteria (Kimmel et al., 1995) and hpf. For the double-transplantation assay, we used *Tg(dharma:GFP)* (Ryu et al., 2001) or *Tg(-1.8gsc:GFP)* (Doitsidou et al., 2002) embryos to visualize the dorsal part of the host embryos. Embryonic manipulations were conducted in Danieau's solution [58 mM NaCl, 0.7 mM KCl, 0.4 mM MgSO_4_, 0.6 mM Ca(NO_3_)_2_, 5.0 mM HEPES (pH 7.2)] or Ringer's solution (116 mM NaCl, 2.9 mM KCl, 1.8 mM CaCl_2_, 5.0 mM HEPES, pH 7.2) unless otherwise described. All zebrafish husbandry, breeding and experiments using zebrafish in this study were approved by the Ethics Committee of ISTAustria and Aoyama Gakuin University.

### Constructs

For the cloning of *hoxb*, *ntl* and *sox17* genes for *in situ* hybridization, the following primer sets were used: *hoxb1a*, 5′-CAGCACAACATCAGCACCAG-3′, 5′-GTTGAGCTCAAGTGTGGCAG-3′; *hoxb1b*, 5′-TACTGCTTAGAGGTTGTAGG-3′, 5′-GATAGTGGCTTGCAGAGACC-3′; *hoxb4a*, 5′-ACTCTCCGGACTACTACAGC-3′, 5′-CAGATAGGCATAGTGTATGG-3′; *hoxb7a*, 5′-GCACCGGTCTCTTCATCATC-3′, 5′-GTCGCCTCCAATTTGATCAG-3′; *hoxb9a*, 5′-TACCATCCCTACATACCGAC-3′, 5′-AACGCCTAGTACCAGTCTGC-3′; *ntl*, 5′-GTCTCGACCCTAATGCAATG-3′, 5′-CTCACAGTACGAACCCGAGG-3′; *sox17*, 5′-GCTCAGTCATGATGCCTGGC-3′, 5′-GTAGGCAAGCTGTAGGACTC-3′. Amplified DNA fragments were cloned into pCR-Blunt II-TOPO vector using Zero Blunt TOPO PCR Cloning kit (Thermo Fisher Scientific). For the full-length cloning of Hoxb genes for mRNA injection, the following primer sets were used: *hoxb1a*, 5′-TCTCGATTTCTCAGGTTGTC-3′, 5′-CGCCACCGTGTACACTGAAG-3′; *hoxb1b*, 5′-ATTTATAGCTCGCTAAACAAATAC-3′, 5′-TTTATCAGGTCACAGGTCCG-3′; *hoxb4a*, 5′-ATGGCCATGAGTTCCTATTTG-3′, 5′-TTGACAAAACAGAGCGACTC-3′; *hoxb7a*, 5′-ATGAGTTCATTGTATTATGCGAACGCGC-3′, 5′-GTAGTTTATACATCTATATTAAAATG-3′; *hoxb9a*, 5′-AATCACTTCGCTCGAAGCGC-3′, 5′-TTTGTGGTCTATATGTAAAC-3′. Full-length Hoxb genes were subcloned into pCS2 vector. The zebrafish caRhoA plasmid was constructed as previously described (Takesono et al., 2012).

### Whole-mount *in situ* hybridization

Embryos were dechorionated using watchmaker forceps in Danieau's or Ringer's solution and fixed at different stages by 4% paraformaldehyde (in PBS) for 2 h at room temperature (RT) and then overnight at 4°C. After fixation, embryos were washed three times in PBS for 10 min at RT, then transferred into methanol of increasing concentrations (25%, 50%, 75% methanol/PBS and 100% methanol) and stored at −20°C overnight. Probes for *in situ* hybridization were synthesized from cloned cDNA sequences (described above) using SP6 RNA polymerase (10810274001, Roche) or T7 RNA polymerase (10881767001, Roche) with DIG labeling mix (11277073910, Roche). Whole-mount *in situ* hybridization for zebrafish embryos was performed as described previously (Montero et al., 2005) except that BM Purple (11442074001, Roche) was used for coloration. After completion of *in situ* hybridization process, samples were transferred into glycerol of increasing concentrations (25%, 50%, 75% glycerol/PBS and 100% glycerol) and then imaged on a Leica MZ165FC microscope.

### Cryosectioning of whole-mount *in situ* hybridization samples

Stained embryos were transferred into PBS of increasing concentrations (75%, 50%, 25% glycerol/PBS and 100% PBS) and then incubated in sucrose of increasing concentrations [5%, 15%, 30% (w/v) sucrose/PBS solution] at 4°C overnight. Samples were then placed in a 1:1 solution of 30% sucrose and optimal cutting temperature compound (OCT; Tissue-Tek) for 2 h at 4°C, then embedded in disposal molds with OCT and frozen at −80°C. Frozen samples embedded in OCT were sectioned with 12 µm thickness on a Leica CM3050 S cryostat. Sections were mounted on glass slides and allowed to dry at RT. Dried samples were washed in PBS and mounted in 75% glycerol/PBS.

### Morpholinos and mRNA injection

Zebrafish embryos were injected using glass capillary needles (30-0020, Harvard Apparatus), which were pulled using a needle puller (P-97 IVF, Sutter Instrument) and attached to a microinjection system (PV820, World Precision Instruments). Antisense MOs were designed to block translation and purchased from Gene Tools, LLC. The sequences of MOs used in this study are as follows: *hoxb1a*, 5′-CTGTCCATACGCAATTAATGGCGGA-3′; *hoxb1b*, 5′-AATTGCTGTGTCCTGTTTTACCCAT-3′; *hoxb4a*, 5′-GTACACGGTTCAGTATCCATATTTC-3′; *hoxb7a*, 5′-CGTTCGCATAATACAATGAACTCAT-3′; *hoxb9a*, 5′-TCCAGAAATGGACATTCTCGGACAT-3′; control, 5′-CCTCTTACCTCAGTTACAATTTATA-3′ (supplied as standard control morpholino oligo by Gene Tools, LLC). Two nanograms of these MOs were injected into one-cell-stage embryos. mRNAs of Hoxb genes cloned in pCS2 vector were synthesized using the mMESSAGE mMACHINE SP6 kit (AM1340, Invitrogen) according to the manufacturer's instructions and 100 pg of mRNA was injected into one-cell-stage embryos; 100 pg memRFP, 75 pg caMypt1, 1 pg caRhoA, 250 pg dnEzrin, or 50 pg Lifeact-mCherry mRNAs were injected into one-cell-stage embryos.

### CRISPR/Cas9 mutant generation

For target site determination of CRISPR/Cas9 mutants for *hoxb1b* (ENSDARG00000054033), *hoxb4a* (ENSDARG00000013533), *hoxb7a* (ENSDARG00000056030) and *hoxb9a* (ENSDARG00000056023) genes, the CHOPCHOP website was used (http://chopchop.cbu.uib.no) (Labun et al., 2016; Montague et al., 2014). In all cases, target sites were designed in the first exon of each gene. To generate gRNA, the following oligonucleotide sets were designed: *hoxb1b*, 5′-TAGGggATTGGCTCCCATATTCACGA-3′, 5′-AAACTCGTGAATATGGGAGCCAATcc-3′; *hoxb4a*, 5′-TAGGggAGTCCGGAGAGTGACTGGGT-3′, 5′-AAACACCCAGTCACTCTCCGGACTcc-3′; *hoxb7a*, 5′-TAGGggTCATCCACGGGTAGATTCGG-3′, 5′-AAACCCGAATCTACCCGTGGATGAcc-3′; *hoxb9a*, 5′-TAGGggCGCGTCTAATGGCTTACCCG-3′, 5′-AAACCGGGTAAGCCATTAGACGCGcc-3′. In the oligonucleotide sequences, the adding sequences for the ligation into the BsaI-cut pDR274 plasmid are underlined, and the adding sequences for the high efficiency for gRNA transcription *in vitro* are shown in lower case. To anneal and generate linker DNAs using the above-mentioned oligonucleotides, 9 µl of 100 µM oligonucleotide sets each and 2 µl of 10× M buffer (Takara Bio) were mixed and incubated at 72°C for 10 min, and then the incubator was turned off and the mixture was left for 20 min to cool down. After putting on ice, 180 µl of TE buffer was added. The linker DNAs were then cloned into BsaI-cut pDR274 plasmid and the cloned plasmids were cut with HindIII, and transcribed using the MEGAshortscript T7 Transcription kit (Ambion). One to two nanoliters of a mixture of 0.2 µl of 350-400 ng/µl gRNA, 2 µl of 250-300 ng/µl Cas9 mRNA, 0.2 µl of 350-400 ng/µl tyrosinase gRNA and 0.2 µl of 1 M KCl was injected into one-cell-stage embryos. Tyrosinase gRNA was used G0 screening (Jao et al., 2013). Cas9 mRNA was synthesized using the mMESSAGE mMACHINE SP6 Transcription kit (Ambion) according to the manufacturer's instructions. For the identification of mutant fish with germline transmission, hetero-duplex mobility assay (HMA) was used (Ota et al., 2014). Primer sequences used for HMA were as follows: *hoxb1b*, 5′-CGTTCTCACTCAAGCAGATGAC-3′, 5′-ATGATTGATAGTGGCTTGCAGA-3′; *hoxb4a*, 5′-ACCCTGCGAGGAATATTCCC-3′, 5′-TGCTGGAACGAGGGGTCTTG-3′; *hoxb7a*, 5′-AGAGCAGAGGGGCTACCATC-3′, 5′-GTTTTCACAGACCTGTGCTC-3′; *hoxb9a*, 5′-TCCAATGTACAGTACTCCAGCG-3′, 5′-GGTATCGAGTATCCGTTGAAGG-3′. For the identification of the mutated sequences, the amplified PCR products were cloned into the Zero Blunt TOPO PCR vector and sequenced by the sequencing service of Eurofins Genomics. Identified mutant fish were outcrossed with wild-type fish to generate heterozygous lines. Homozygous mutants were obtained by incross of heterozygous fish. Maternal-zygotic mutants were obtained by crossing homozygous females with heterozygous or homozygous males. Mutant embryos were genotyped by PCR using the primers that were used for HMA. Hoxb mutant lines with *Tg(-1.8gsc:GFP)* background were established by crossing Hoxb mutants with the *Tg(-1.8gsc:GFP)* line.

### Cell lineage tracing with DiI

For cell tracing, DiI (D3911, Invitrogen; 2.5 mg/ml in DMSO) was injected into the lateral blastoderm margin at different stages (50%, 60% or 70% epiboly). Labeled embryos were then placed into agarose molds and imaged on a Leica MZ165FC microscope.

### Visualization of intercellular spaces

For visualizing intercellular space (interstitial fluid), embryos were injected with 2.5 pg of Dextran Mini-Ruby (D3312, Thermo Fisher Scientific) into intercellular spaces of the lateral blastoderm margin at 40% epiboly.

### Double cell transplantation

For double cell transplantations (cells from two donor embryos), one donor embryo was injected with 2.5 ng of Dextran Cascade Blue (D1976, Thermo Fisher Scientific) and the other donor embryos was injected with *hoxb1b* MO/mRNA or *hoxb7a* MO/mRNA, together with 2.5 ng of Dextran Mini-Ruby (D3312, Thermo Fisher Scientific). Host embryos were injected 100 pg membrane-bound RFP (memRFP) mRNA (Iioka et al., 2004). For the analysis of transplanted cell localization at the 12-somite stage, the two donor embryos were prepared as described above, and the host embryos remained unlabeled. For the analysis of transplanted cell protrusive activity, one donor embryo was injected with a combination of 50 pg Lifeact-mCherry and Dextran Cascade Blue, and the other donor embryo was injected with a combination of 50 pg Lifeact-mCherry and *hoxb1b* MO or *hoxb7a* mRNA. For determining the bleb frequency of transplanted cells, one donor embryo was injected with 2.5 ng of Dextran Cascade Blue and the other donor embryo was injected with *hoxb1b* MO/mRNA or *hoxb7a* MO/mRNA, together with 2.5 ng of Dextran Mini-Ruby. All of these injections were performed in one-cell-stage *Tg(dharma:GFP)* or *Tg(-1.8gsc:GFP)* embryos, which, once injected, were incubated at 31°C until they had reached 30% epiboly. Embryos were then dechorionated by forceps and transferred into 2% agarose molds within Danieau's or Ringer's solution; around 10-20 cells (for determining ingression timing and distribution pattern) or 5-10 cells (for observation of transplanted cell behavior) were aspirated from the lateral blastoderm margin of the donor embryo, using a beveled borosilicate needle with a 20 µm inner diameter (Biomedical Instruments) attached to a syringe, and then transplanted into the corresponding region of the host embryo. This procedure was repeated for the second donor embryo and the order of transplantations/donor embryos was randomized to avoid artifacts due to the order of transplantations. To determine the timing of cell ingression following transplantation, we defined the ingressed state as the point at which cells enter the hypoblast at the blastoderm margin. For the characterization of transplanted cell dynamics, we defined the frequent blebbing state as cells exhibiting more than five blebs within a 3-min interval.

### Imaging

Brightfield imaging for developing embryos and *in situ* hybridization samples was performed on a Leica microscope MZ165FC equipped with a PLANAPO 1.0× objective using LAS version 4.8 software. For imaging, developing embryos were dechorionated by forceps, and placed in 1% agarose molds within Danieau's solution; *in situ* hybridization samples were transferred into glycerol and mounted in 100% glycerol. For determining cell dynamics during mesendoderm cell ingression, embryos were mounted in glass bottom dishes (Matsunami) within 0.7% low melting point (LMP) agarose (16520050, Invitrogen) and imaged on an FV3000 inverted confocal microscope (Olympus). For determining the timing of transplanted mesendoderm cell ingression, transplanted embryos were mounted within 0.7% LMP agarose on a mold made by 2% agarose and imaged on a TriM Scope two-photon microscope (LaVision BioTec) equipped with a Chameleon Ultra II laser with Chameleon Compact OPO (Coherent), a Plan-Apochromat 20×/1.0 water-immersion objective (Zeiss) and GaAsP detectors (Hamamatsu Photonics). Mesendodermal cell ingression was monitored in a 400×200 µm area at the blastoderm margin with a 170 µm deep *z*-stack in 2 µm step sizes and time intervals were 4-6 min. Images were taken with excitation wavelength of 830 nm using a Ti-Sapphire femtosecond laser system (Coherent Chameleon Ultra) and 1100 nm optical parametric oscillator (Coherent Chameleon Compact OPO). For determining the distribution pattern of transplanted mesendodermal cells, the transplanted embryos were incubated until they had reached the 12-somite stage and then transplanted embryos were mounted within 0.7% LMP agarose on a mold made of 2% agarose and imaged. For monitoring mesendodermal cell behavior, transplanted embryos were mounted within 0.7% LMP agarose on a mold made of 2% agarose and imaged on an upright Zeiss confocal microscope LSM 900 equipped with a Plan-Apochromat 20×/1.0 water-immersion objective (Zeiss) using Zen software. The range of the imaged *z*-stack was set to capture all transplanted cells with a step size of 2-3 µm and a time interval of 30 s. Sectioned *in situ* hybridization samples were imaged on an Olympus BX53 microscope using cellSens software (Olympus).

### Analysis of cell movement

Cell movement tracks were manually obtained by a FluoView FV31S-DT (Olympus). The timing of transplanted cell ingression and exhibiting frequent blebbing were determined using Imaris (version 7.4, Bitplane). We defined the cells as ‘ingressed’ when cells had entered the hypoblast at the blastoderm margin, and defined ‘frequent blebbing’ as the state when cells were forming more than five blebs within 3 min. Protrusions (actin-rich protrusions and blebs) of transplanted cells were manually identified using Zen (Zeiss), Fiji (NIH) and Imaris (version 7.4, Bitplane).

### Quantification of cell surface fluctuations

Quantification of cell surface fluctuations was performed as previously described (Yanagida et al., 2022). Cell shapes, visualized on a Zeiss LSM 900 upright confocal microscope by Dextran Cascade Blue or Dextran Mini-Ruby staining, were determined by binarization of single *z*-plane images and the distance from the center of the cell to its margin (ρ) was measured. Using a custom-written MATLAB script, the outline of cell shapes was determined and converted from Cartesian into polar coordinates. Surface fluctuations as a mean of all V_j_ were normalized by the mean of radius of each cell.

### Statistical analysis

Statistical analyses for each experiment are described in the corresponding figure legends. All statistical analyses and graph generation were performing using GraphPad Prism 9. Analyzed samples were not subjected to any inclusion or exclusion criteria, and no statistical tests were conducted to assess the sample size. Prior to selecting the appropriate statistical tests, data were assessed for normality using D'Agostino–Pearson normality test. For comparisons between two groups, a two-sided Student's *t*-test was used for normally distributed data, while Mann–Whitney test was applied for non-normally distributed data. For comparisons among more than two groups, a one-way ANOVA was used for normally distributed data, and the Kruskal–Wallis test for non-normal distributed data. *n* indicates the number of individual cells or embryos analyzed per experiment, and *N* refers to the number of independent experimental replicates (embryos).

## Supplementary Material



10.1242/develop.204261_sup1 Supplementary information
