# Supplementary material for: Hoxb genes determine the timing of cell ingression by regulating cell surface fluctuations during zebrafish gastrulation
Source: Development. 2025 Jun 27;152(12):dev204261. doi: 10.1242/dev.204261 (PMC12273642; doi:10.1242/dev.204261)
Supplement: Supplementary information [file develop-152-204261-s1.pdf]

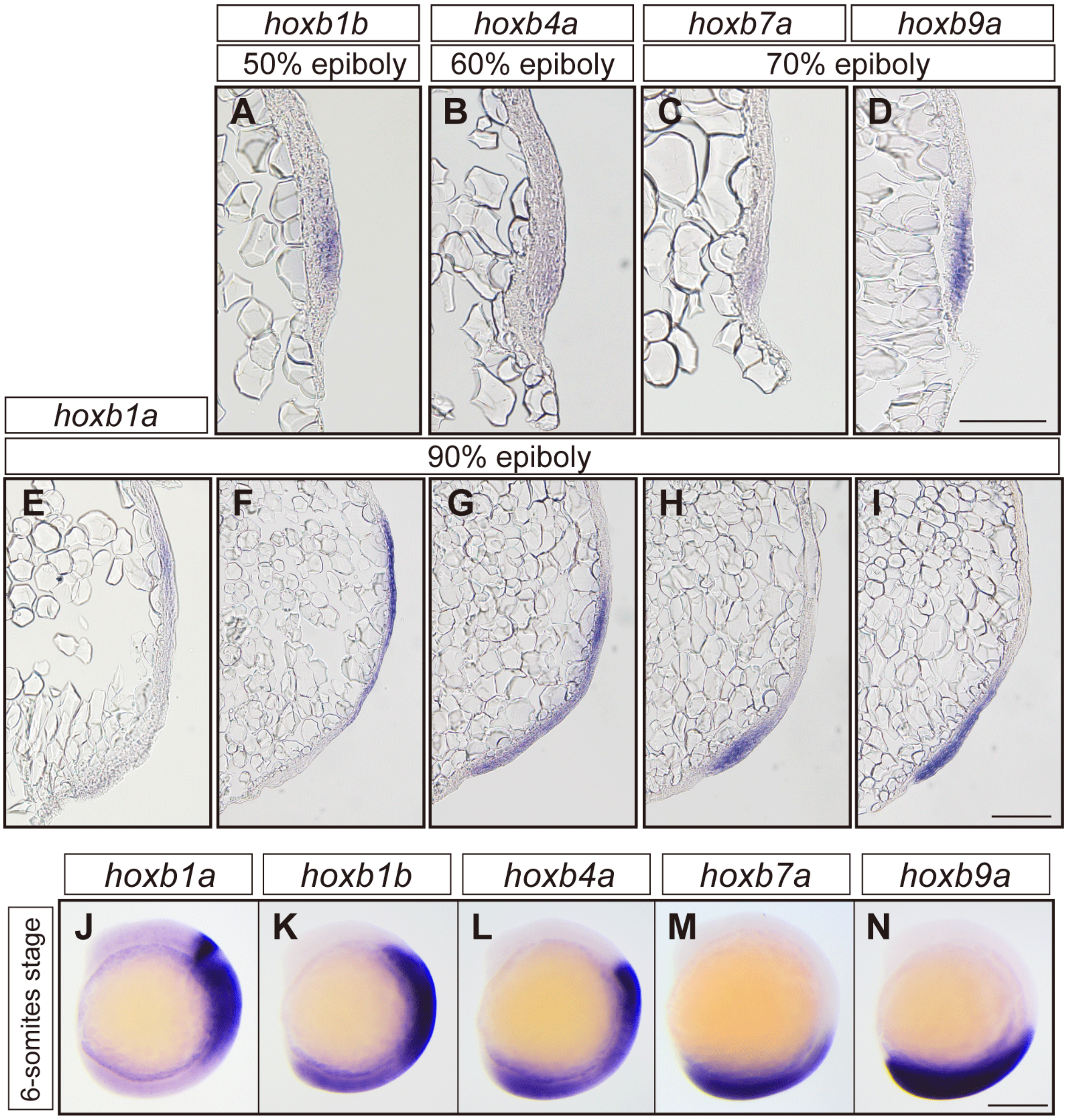

Fig. S1. Hoxb gene expression during gastrulation and somite stage. (A-D) Bright-field images of cryosections of whole mount *in situ* hybridization in embryos showing *hoxb1b* at 50% (A), *hoxb4a* at 60% epiboly (B), *hoxb7a* at 70% epiboly (C), and *hoxb9a* at 70% epiboly (D). Only A is a tilted lateral view for capturing *hoxb1b* expression, and the others are lateral views. Scale bar, 20 µm. (E-I) Bright-field images (lateral views) of cryosections of whole mount *in situ* hybridization in embryos showing *hoxb1a* (E), *hoxb1b* (F), *hoxb4a* (G), *hoxb7a* (H), and *hoxb9a* (I) at 90% epiboly. Scale bar, 20 µm. (J-N) Bright-field images (lateral views) of expression patterns of *hoxb1a* (J), *hoxb1b* (K), *hoxb4a* (L), *hoxb7a* (M), and *hoxb9a* (N) at 6 somite stage. Scale bar, 200 µm.

Development • Supplementary information

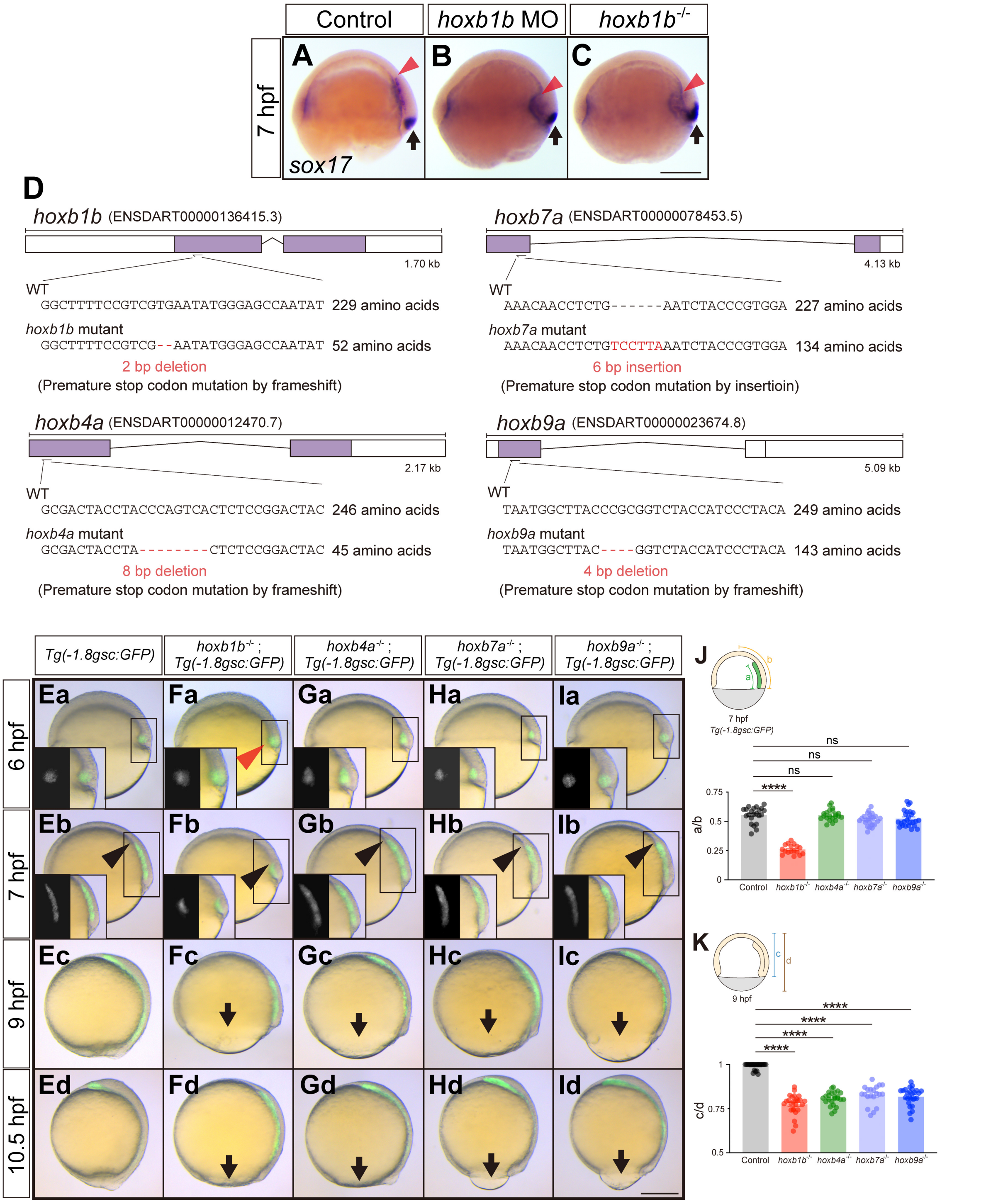

Fig. S2. Mesendoderm formation and gastrulation defects in Hoxb mutants. (A-C) *sox17* expression patterns in control, *hoxb1b* MO-injected (knockdown) and *hoxb1b*$^{-/-}$ embryos at 7 hpf. Endodermal cells migration towards the animal pole is reduced in *hoxb1b* knockdown and *hoxb1b*$^{-/-}$ embryos. Red arrowheads point at the anterior limit of the *sox17*

Development • Supplementary information

expression domain. Arrows depict dorsal forerunner cells. Scale bar, 200 μm. (D) Design of CRISPR/Cas9-mediated knockout for each hoxb gene and resultant mutated sequences. Mutants were generated by introducing a premature stop codon through frameshift (*hoxb1b, hoxb4a* and *hoxb9a*) or stop codon insertion (*hoxb7a*). (E-J) Overlay of bright-field and fluorescence images of control (*Tg(-1.8gsc:GFP)*, E), *hoxb1b*$^{-/-}$; *Tg(-1.8gsc:GFP)*(F), *hoxb4a Tg(-1.8gsc:GFP)*(G), *hoxb7a*$^{-/-}$; *Tg(-1.8gsc:GFP)*(H) and *hoxb9a*$^{-/-}$; *Tg(-1.8gsc:GFP)*(I) embryos at 6 (a), 7 (b), 9 (c), and 10.5 (d) hpf. Insets are bright-field and fluorescence images. Red arrowheads point at defective mesedndoderm ingression at the dorsal blastoderm margin at 6 hpf. Black arrowheads point at the leading edge of mesendodermal cells migrating towards the animal pole. Arrows, the blastoderm margin for the embryos exhibiting epiboly delay. Dorsal side is to the right. Scale bar, 200 μm. (J) Quantification of animal pole directed migration of mesendodermal cells [GFP positive cells in *Tg(-1.8gsc:GFP)*] by the ratio of the length of the GFP-positive ingressed mesendoderm (a) to the distance from the blastoderm margin to the animal pole (b) in *Tg(-1.8gsc:GFP)* (*n*=22, *N*=3), *hoxb1b*$^{-/-}$; *Tg(-1.8gsc:GFP)* (*n*=17, *N*=3), *hoxb4a*$^{-/-}$; *Tg(-1.8gsc:GFP)* (*n*=21, *N*=3), *hoxb7a*$^{-/-}$; *Tg(-1.8gsc:GFP)* (*n*=23, *N*=3), and *hoxb9a*$^{-/-}$; *Tg(-1.8gsc:GFP)* (*n*=25, *N*=3)-injected embryos at 7 hpf. One-way ANOVA; ns, *P*>0.05; ****P<0.0001. Data are shown as means ±s.e.m. (K) Quantification of epiboly progression by the ratio of the blastoderm length (c) to the total embryo length from the animal pole to the vegetal pole (d) in *Tg(-1.8gsc:GFP)* (*n*=23, *N*=3), *hoxb1b*$^{-/-}$; *Tg(-1.8gsc:GFP)* (*n*=27, *N*=3), *hoxb4a*$^{-/-}$; *Tg(-1.8gsc:GFP)* (*n*=24, *N*=3), *hoxb7a*$^{-/-}$; *Tg(-1.8gsc:GFP)* (*n*=20, *N*=3), and *hoxb9a*$^{-/-}$; *Tg(-1.8gsc:GFP)* (*n*=27, *N*=3)-injected embryos at 9 hpf. Kruskal-Wallis test; ns, *P*>0.05; ****P<0.0001. Data are shown as means ±s.e.m.

Development • Supplementary information

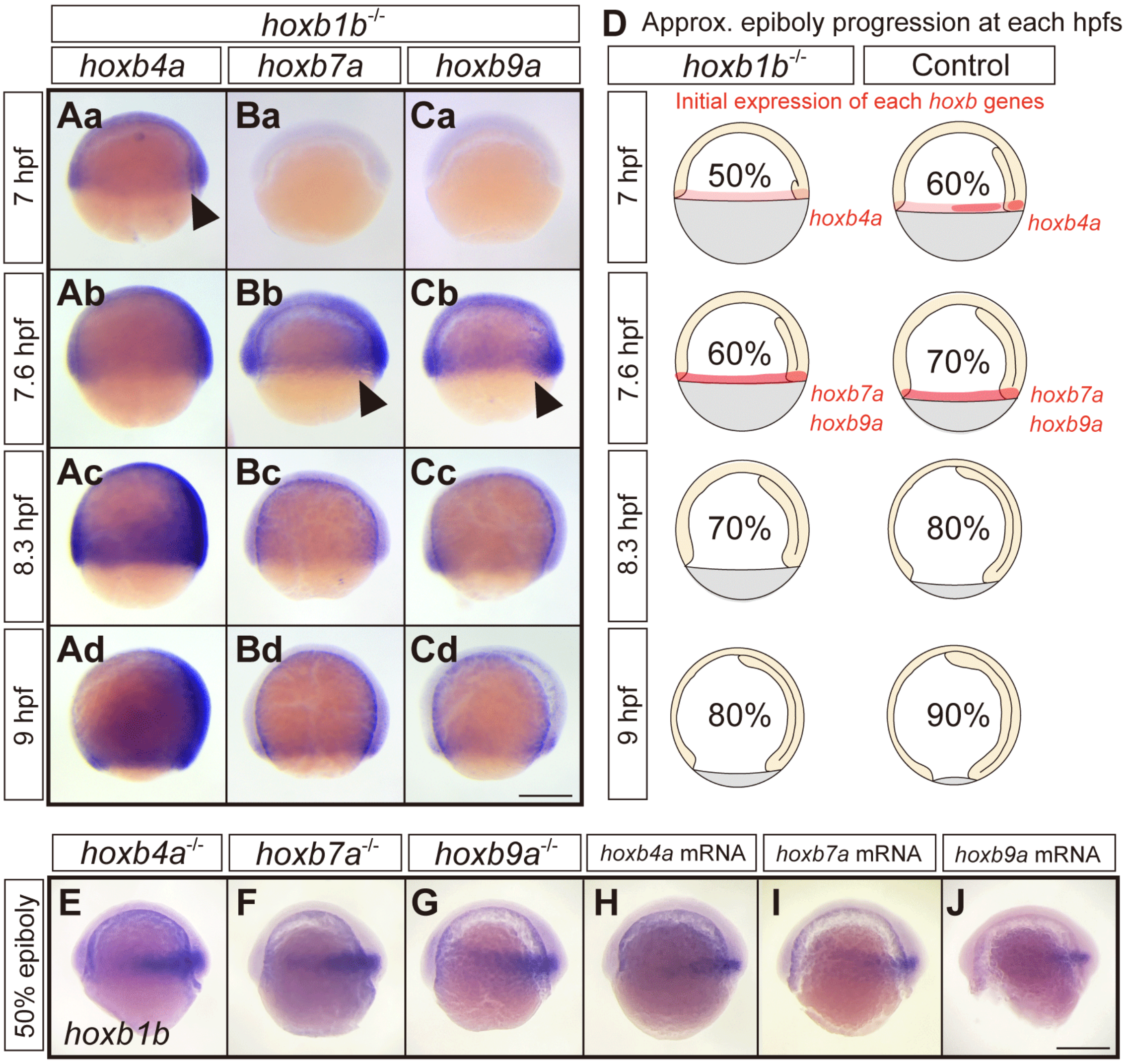

Fig. S3. Hoxb gene expression patterns in Hoxb knockout and overexpressing embryos. (A-C) *hoxb4a, hoxb7a* and *hoxb9a* expression patterns in *hoxb1b* mutant embryos at 7 (a), 7.6 (b), 8.3 (c), and 9 (d) hpf. (D) Schematic representation of approximate epiboly progression in *hoxb1b* mutant and control embryos. Red bars represent initial expression patterns of each *hoxb* genes at blastoderm margin. (E-J) *hoxb1b* expression patterns in *hoxb4a*$^{-/-}$, *hoxb7a*$^{-/-}$, and *hoxb9a*$^{-/-}$ embryos (E-G) and *hoxb4a*, *hoxb7a* and *hoxb9a* mRNA-injected embryos (H-J) at 50% epiboly. Dorsal side is to the right. Scale bar, 200 μm.

Development • Supplementary information

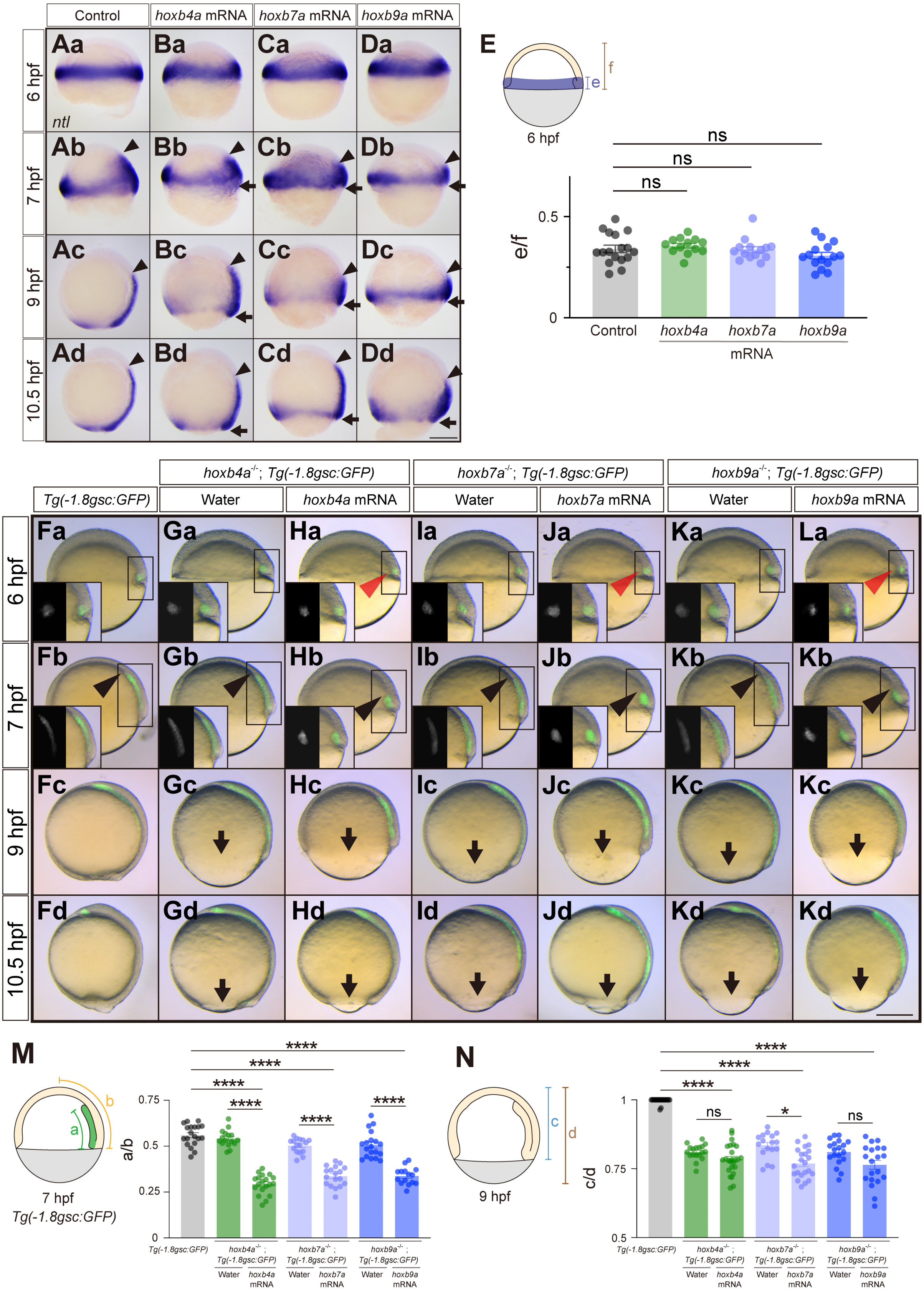

Development • Supplementary information

Fig. S4. ***ntl*** **expression in embryos overexpressing** ***hoxb4a, hoxb7a*****, or** ***hoxb9a*****, and phenotypes resulting from injection of each Hoxb mRNA into the respective mutant embryos.** (A-D) *ntl* expression in water-injected control (A), *hoxb4a* mRNA(B), *hoxb7a* mRNA(C), and *hoxb9a* mRNA(D)-injected embryos at 6 hpf (a), 7 hpf (b), 9 hpf (c), or 10.5 hpf (d). Arrowheads demarcate the anterior limit of *ntl* expression at the dorsal side. Arrows point at the blastoderm margin in embryos with delayed epiboly. Lateral views. Dorsal side is to the right. Scale bar, 200 μm. (E) Quantification of *ntl* expression domain at 6 hpf by the ratio of *ntl* expression domain (e) to the blastoderm length (f) in water-injected control (n=17), *hoxb4a* mRNA(n=13), *hoxb7a* mRNA(n=14), or *hoxb9a* mRNA(n=15)-injected embryos. One-way ANOVA; ns, P>0.05. Data are shown as means ±s.e.m. (F-N) Overlay of bright-field and fluorescence images of *Tg(-1.8gsc:GFP)* (control, F), water-injected *hoxb4a*$^{-/-}$; *Tg(-1.8gsc:GFP)* (G), *hoxb4a* mRNA-injected *hoxb4a*$^{-/-}$; *Tg(-1.8gsc:GFP)* (H), water-injected *hoxb7a*$^{-/-}$; *Tg(-1.8gsc:GFP)* (I), *hoxb7a* mRNA-injected *hoxb7a*$^{-/-}$; *Tg(-1.8gsc:GFP)* (J), water-injected *hoxb9a*$^{-/-}$; *Tg(-1.8gsc:GFP)* (K), and *hoxb9a* mRNA-injected *hoxb9a*$^{-/-}$; *Tg(-1.8gsc:GFP)* embryos (L) at 6 hpf (a), 7 hpf (b), 9 hpf (c), and 10.5 hpf (d). (G) Quantification of animal pole directed migration of mesendodermal cells [GFP positive cells in *Tg(-1.8gsc:GFP)*] by the ratio of the length of the GFP-positive ingressed mesendoderm (a) to the distance from the blastoderm margin to the animal pole (b) in *Tg(-1.8gsc:GFP)* (n=16, N=3), water-injected *hoxb4a*$^{-/-}$; *Tg(-1.8gsc:GFP)* (n=19, N=3), *hoxb4a* mRNA-injected *hoxb4a*$^{-/-}$; *Tg(-1.8gsc:GFP)* (n=16, N=3), water-injected *hoxb7a*$^{-/-}$; *Tg(-1.8gsc:GFP)* (n=18, N=3), *hoxb7a* mRNA-injected *hoxb7a*$^{-/-}$; *Tg(-1.8gsc:GFP)* (n=19, N=3), water-injected *hoxb9a*$^{-/-}$; *Tg(-1.8gsc:GFP)* (n=15, N=3), and *hoxb9a* mRNA-injected *hoxb9a*$^{-/-}$; *Tg(-1.8gsc:GFP)* (n=19, N=3) embryos at 7 hpf. One-way ANOVA; ns, P>0.05; ****P<0.0001. Data are shown as means ±s.e.m. (H) Quantification of epiboly progression by the ratio of the blastoderm length (c) to the total embryo length from the animal pole to the vegetal pole (d) in *Tg(-1.8gsc:GFP)* (n=18, N=3), water-injected *hoxb4a*$^{-/-}$; *Tg(-1.8gsc:GFP)* (n=25, N=3), *hoxb4a* mRNA-injected *hoxb4a*$^{-/-}$; *Tg(-1.8gsc:GFP)* (n=17, N=3), water-injected *hoxb7a*$^{-/-}$; *Tg(-1.8gsc:GFP)* (n=24, N=3), *hoxb7a* mRNA-injected *hoxb7a*$^{-/-}$; *Tg(-1.8gsc:GFP)* (n=19, N=3), water-injected *hoxb9a*$^{-/-}$; *Tg(-1.8gsc:GFP)* (n=20, N=3), and *hoxb9a* mRNA-injected *hoxb9a*$^{-/-}$; *Tg(-1.8gsc:GFP)* (n=20, N=3) embryos at 9 hpf. Kruskal-Wallis test; ns, P>0.05; *P<0.05; ****P<0.0001. Data are shown as means ±s.e.m.

Development • Supplementary information

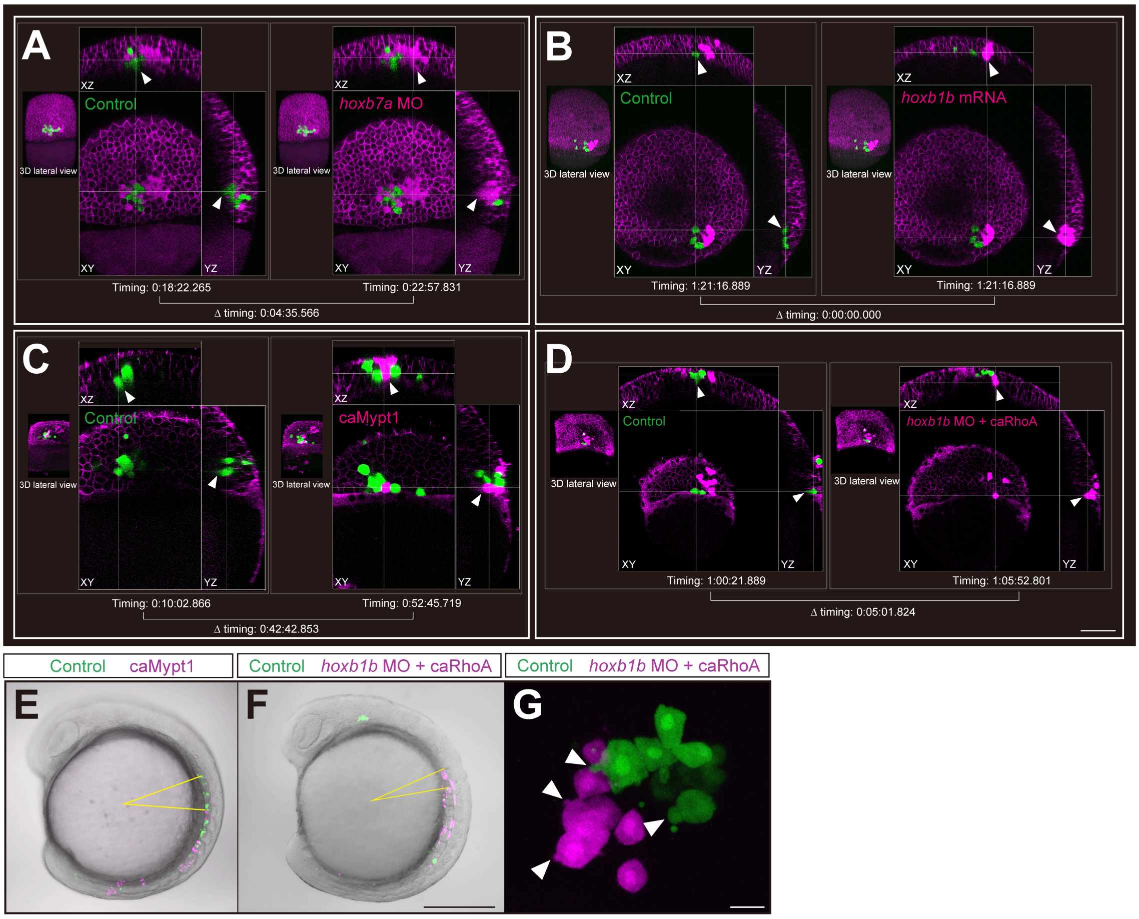

Fig. S5. Ingression timings of *hoxb7a* morphant, *hoxb1b* overexpressing, caMypt1 mRNA-injected, or *hoxb1b* MO plus caRhoA mRNA-injected transplanted cells, distribution pattern of caMypt1 or *hoxb1b* MO plus caRhoA mRNA-injected transplanted cells in somites, and cell blebbing behavior of *hoxb1b* MO plus caRhoA mRNA-injected transplanted cells. (A-D) Time needed for mesendodermal cells to complete their ingression for control cells (green, left) co-transplanted with *hoxb7a* MO (A), *hoxb1b* mRNA (B), caMypt1 mRNA (C), or *hoxb1b* MO plus caRhoA (D)-injected cells (magenta, right). Scale bar, 100 μm. (E,F) Representative image of distribution pattern of caMypt1 mRNA (E) and *hoxb1b* MO plus caRhoA (F)-injected transplanted cells in 12-somite stage. Yellow lines outline the angles between the most anteriorly located control cells (green) and co-transplanted caMypt1 (E) or *hoxb1b* MO plus caRhoA (F)-mRNA injected cells (magenta). Scale bar, 200 μm. (G) Blebbing behavior of transplanted control (green) and *hoxb1b* MO plus caRhoA mRNA-injected (magenta) cells at 50% epiboly stage. For quantification and statistical analysis, see Fig. 6E,L, Fig. 7F,G. Arrowheads depict cellular blebs. Scale bar, 20 μm.

Development • Supplementary information

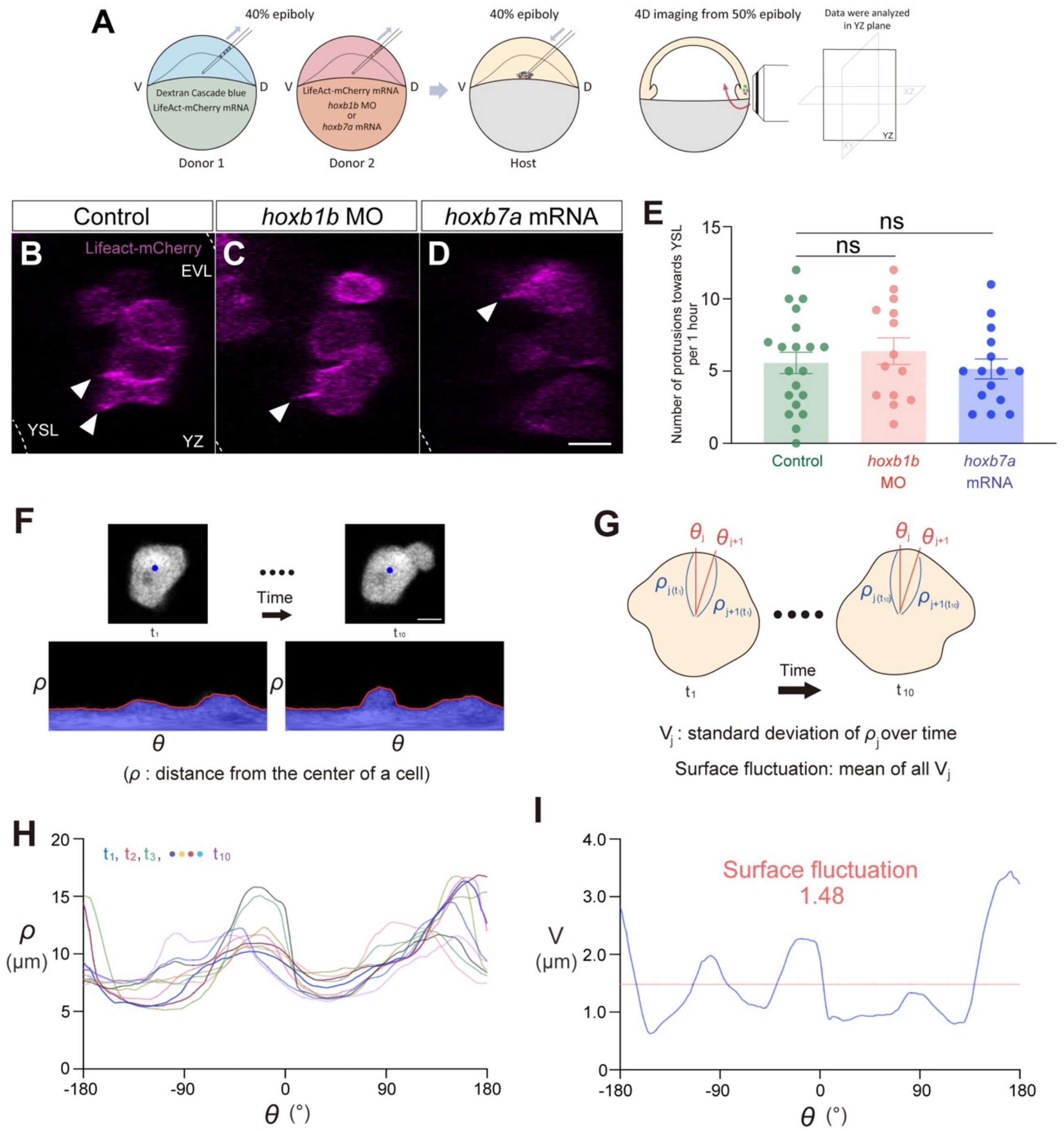

Fig. S6. Analysis of protrusive activity and cell surface fluctuations during ingression. (A) Schematic representation of the double transplantation assay to determine mesendodermal cell protrusive activity during ingression. Embryos injected with dextran cascade blue and Lifeact-mCherry mRNA (donor 1) and embryos injected with a combination of Lifeact-mCherry mRNA and either *hoxb1b* MO or *hoxb7a* mRNA (donor 2) were transplanted into the corresponding region of host embryos. (B-D) High-resolution confocal fluorescence images of transplanted control (B), *hoxb1b* MO (C), and *hoxb7a* mRNA (D)-injected cells labelled with Lifeact-mCherry mRNA. Arrowheads point to cell protrusions directed towards the YSL (left side). Scale bar, 10 µm. YSL, yolk syncytial layer; EVL, enveloping layer. (E) Average number of protrusions directed towards the YSL formed

Development • Supplementary information

per hour in control (*n*=20, *N*=6), *hoxb1b* MO (*n*=12, *N*=4), and *hoxb7a* mRNA (*n*=15, *N*=4)-injected cells. One-way ANOVA; ns, *P*>0.05. Data are shown as means ±s.e.m. (F-I) Quantification of cell surface fluctuations in mesendodermal cells. Fluorescence images of single cells were binarized, and the distance from the cell margin to the center (ρ) was measured around the cell circumference (F). Surface fluctuations were calculated as the mean of the standard deviation of ρ_j over time (V_j) (G). Representatives of calculated ρ (H) at each time point (t_1 to t_{10}) and V (I) as the standard deviation of ρ around the cell circumference and surface fluctuation as the mean of all V. Data are shown as means ±s.e.m. Scale bar, 6 μm.

Development • Supplementary information

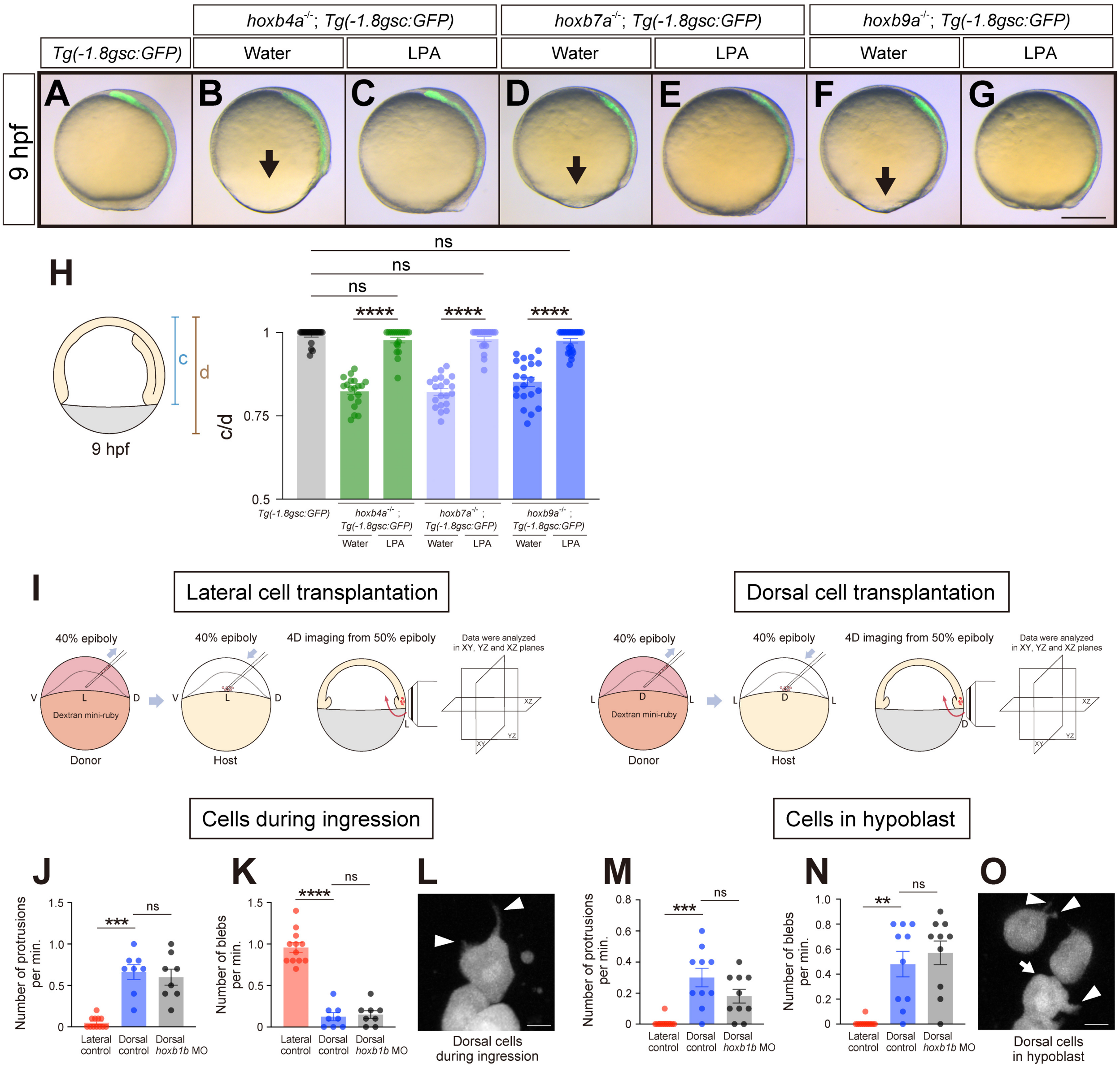

Fig. S7. Effect of LPA treatment on Hoxb mutant phenotypes and cell dynamics at the lateral and dorsal blastoderm margin. (A-G) Epiboly phenotypes of embryos with water-treated control *Tg(-1.8gsc:GFP)* embryos (A), water or LPA-treated *hoxb4a*$^{-/-}$; *Tg(-1.8gsc:GFP)* embryos (B,C), water or LPA-treated *hoxb7a*$^{-/-}$; *Tg(-1.8gsc:GFP)* embryos (D,E), and water or LPA-treated *hoxb9a*$^{-/-}$; *Tg(-1.8gsc:GFP)* embryos (F,G) at 9 hpf. Embryos were treated with water or LPA from 6 hpf. Arrows, the blastoderm margin for the embryos exhibiting epiboly delay. Scale bar, 200 µm. (H) Quantification of epiboly progression by the ratio of the length of the GFP-positive ingressed mesendoderm (a) to the distance from the blastoderm margin to the animal pole (b) in control *Tg(-1.8gsc:GFP)* embryos (*n*=22, *N*=2), water-treated *hoxb4a*$^{-/-}$; *Tg(-1.8gsc:GFP)* (*n*=19, *N*=2), LPA-treated *hoxb4a*$^{-/-}$; *Tg(-1.8gsc:GFP)* (*n*=19, *N*=2), water-treated *hoxb7a*$^{-/-}$; *Tg(-1.8gsc:GFP)* (*n*=20, *N*=2), LPA-treated *hoxb7a*$^{-/-}$; *Tg(-1.8gsc:GFP)* (*n*=19, *N*=2), water-treated *hoxb9a*$^{-/-}$;

Tg(-1.8gsc:GFP) (*n*=21, *N*=2), and LPA-treated *hoxb9a*$^{-/-}$; *Tg(-1.8gsc:GFP)* (*n*=23, *N*=2) embryos at 9 hpf. Kruskal-Wallis test; ns, *P*>0.05; ****P<0.0001. Data are shown as means ±s.e.m. (I) Schematics of lateral (left) and dorsal (right) cell transplantation experiments. (J-L) Quantification of the number of protrusions (J) and blebs (K) per minute in transplanted lateral cells (*n*=12, *N*=3), dorsal cells (*n*=8, *N*=3), and *hoxb1b* morphant dorsal cells (*n*=8, *N*=3), and representative images showing dynamics of transplanted dorsal cell exhibiting protrusions during cell ingression (L). (M-O) Quantification of the number of protrusions (M) and blebs (N) per minute in transplanted lateral cells (*n*=12, *N*=3), dorsal cells (*n*=8, *N*=3), and *hoxb1b* morphant dorsal cells (*n*=8, *N*=3), and representative images showing dynamics of transplanted dorsal cells exhibiting protrusions in the hypoblast (O). One-way ANOVA; ns, *P*>0.05; ***P*<0.01; ****P*<0.001; *****P*<0.0001. Data are shown as means ±s.e.m. Arrowheads depict protrusions, arrows depict blebs. Scale bar, 10 µm.

Development • Supplementary information

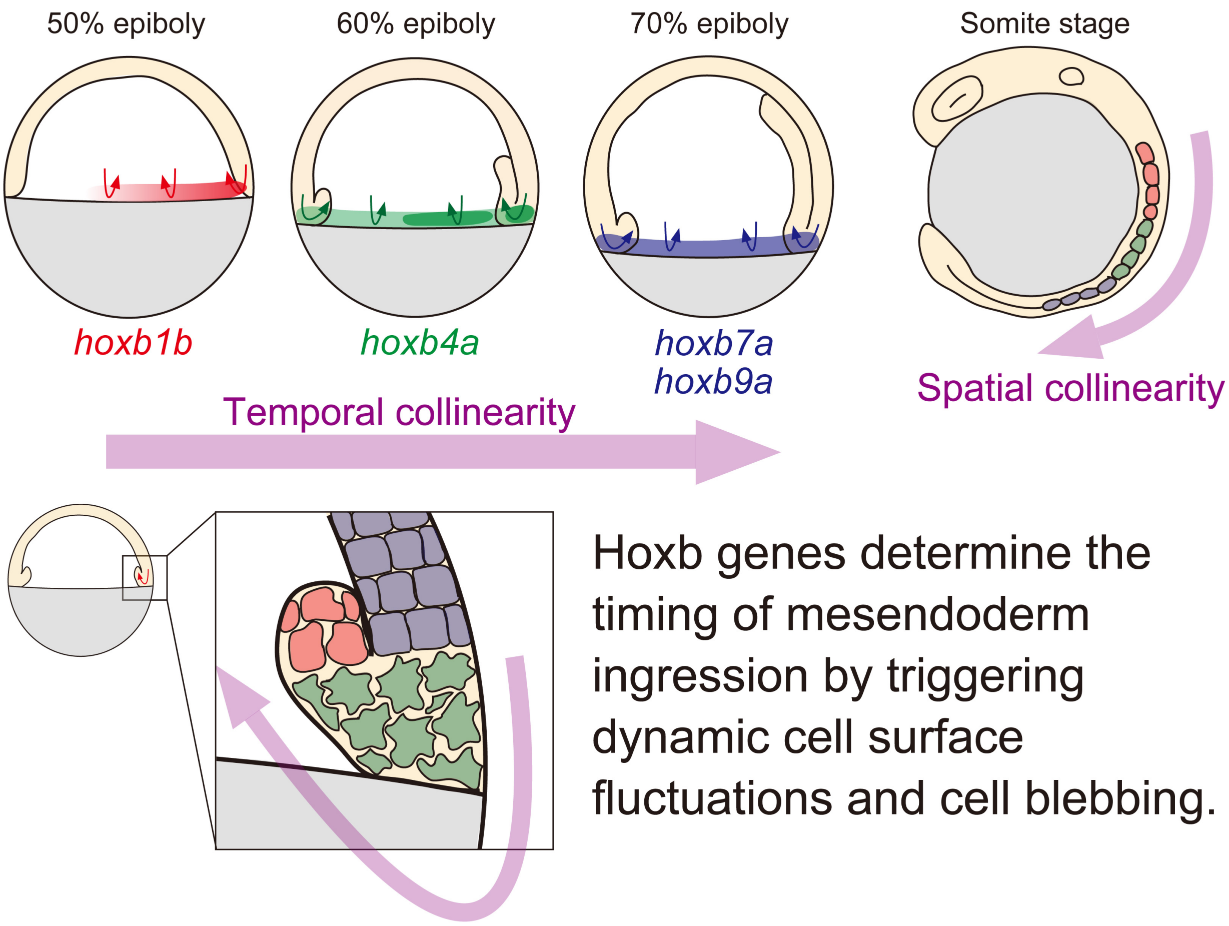

Fig. S8. Hoxb gene expression determines the timing of cell ingression at the blastoderm margin by regulating cell surface fluctuations and associated cell blebbing. Hoxb genes are expressed at the blastoderm margin in a temporally collinear manner and determine the timing of mesendoderm ingression by triggering dynamic cell surface fluctuations and associated cell blebbing. This, in turn, leads to a spatial collinearity of mesendoderm cell positioning along the anterior-posterior axis of the forming body, with early ingressing cells positioned more anteriorly than later ingressing cells.

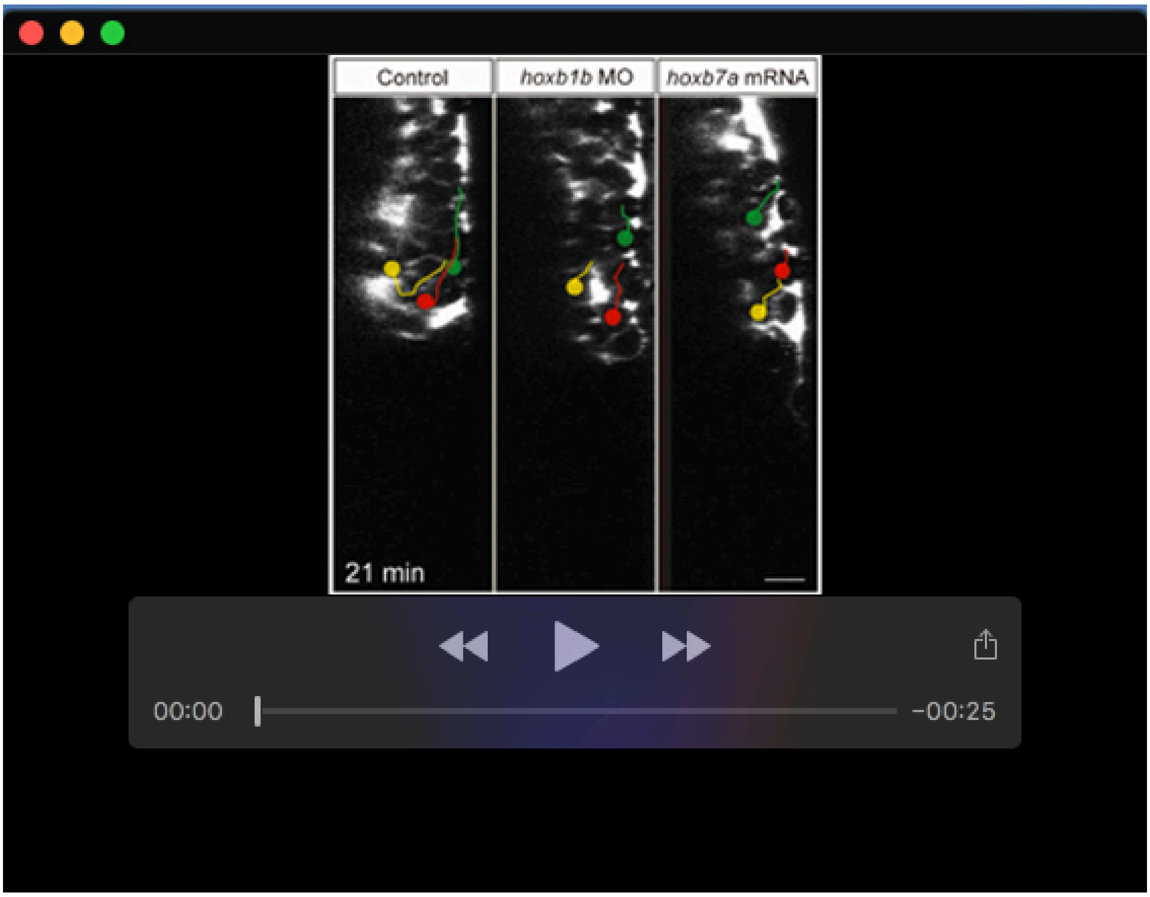

Movie 1. Cell dynamics in control, *hoxb1b* morphants, and *hoxb7a*-overexpressing embryos at the lateral blastoderm margin. Time-lapse movie visualizing cell contours in control (left), *hoxb1b* morphant (middle), and *hoxb7a*-overexpressing (right) embryos. Imaging was started at the 50% epiboly stage (cross-sectional view of lateral side). Intercellular spaces were labelled by injection of dextran mini-ruby at the 40% epiboly stage. Time interval, 3 min. Duration, 1 hour. Scale bar, 20 μm.

Development • Supplementary information

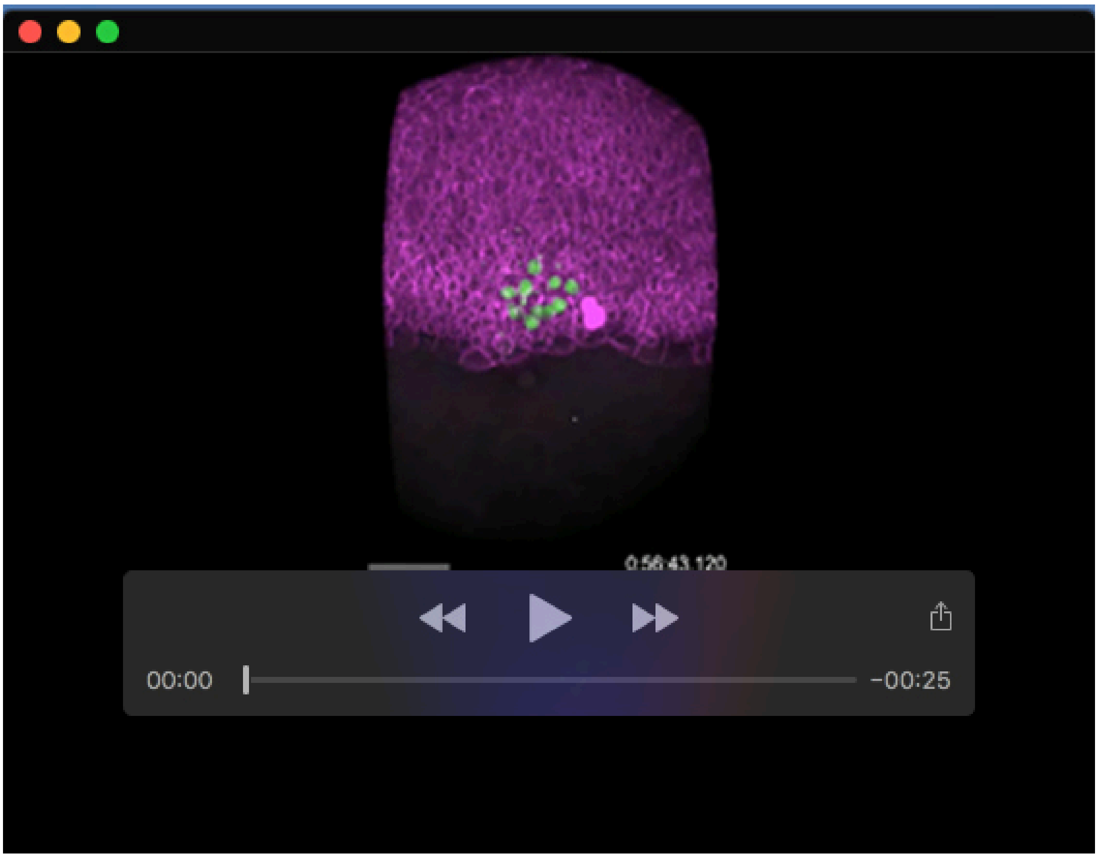

Movie 2. Control cells transplanted to the lateral germ ring margin ingressed in close succession. Time-lapse fluorescence movie visualizing cell ingression of control (green) and control (magenta) cells transplanted to the lateral germ ring margin at the 40% epiboly stage. Imaging was started at the 50% epiboly stage (lateral view). Cell membranes of host embryo were labelled by injection of memRFP mRNA. Time interval, 5 min 9 sec. Duration, 3 hours 21 min. Scale bar, 100 μm.

Development • Supplementary information

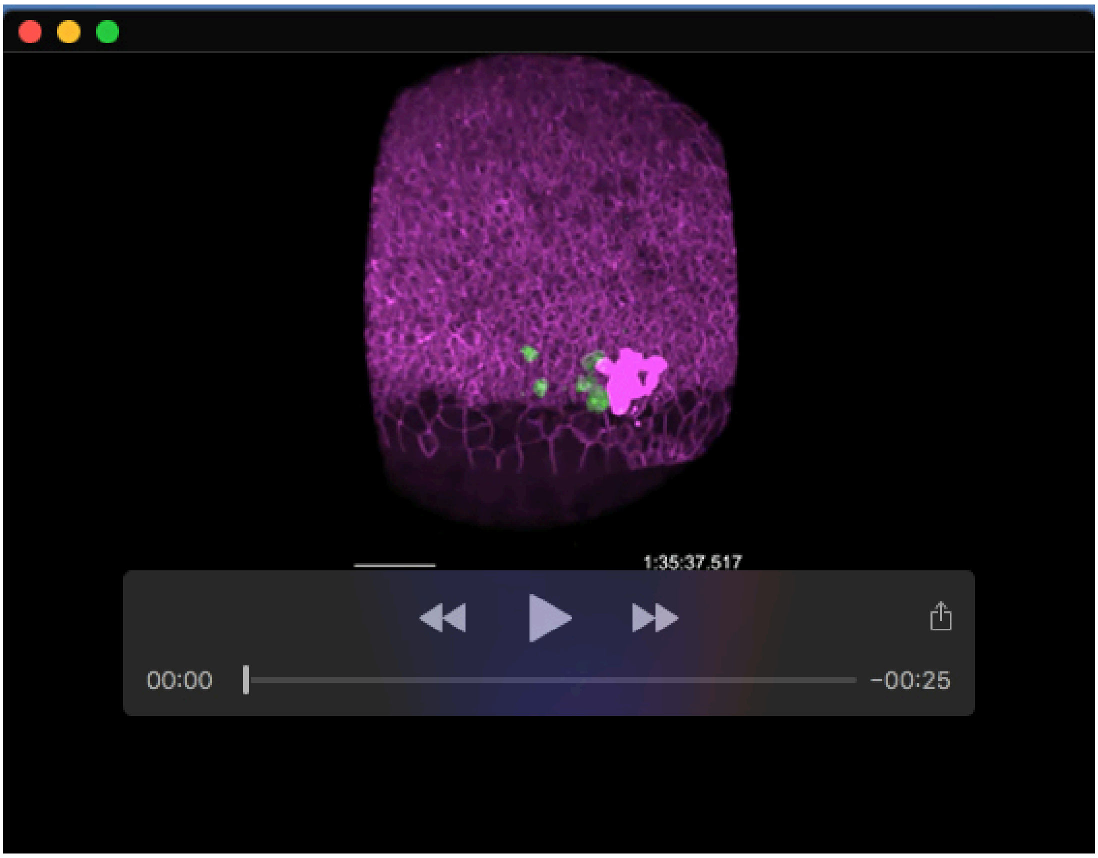

Movie 3. *hoxb1b* morphant cells transplanted to the lateral germ ring margin exhibited delayed ingression compared with those of the control. Time-lapse fluorescence movie visualizing cell ingression of control (green) and *hoxb1b* morphant (magenta) cells transplanted to the lateral germ ring margin at the 40% epiboly stage. Imaging was started at the 50% epiboly stage (lateral view). Cell membranes of host embryo were labelled by injection of memRFP mRNA. Time interval, 4 min 47 sec. Duration, 3 hours 6 min. Scale bar, 100 μm.

Development • Supplementary information

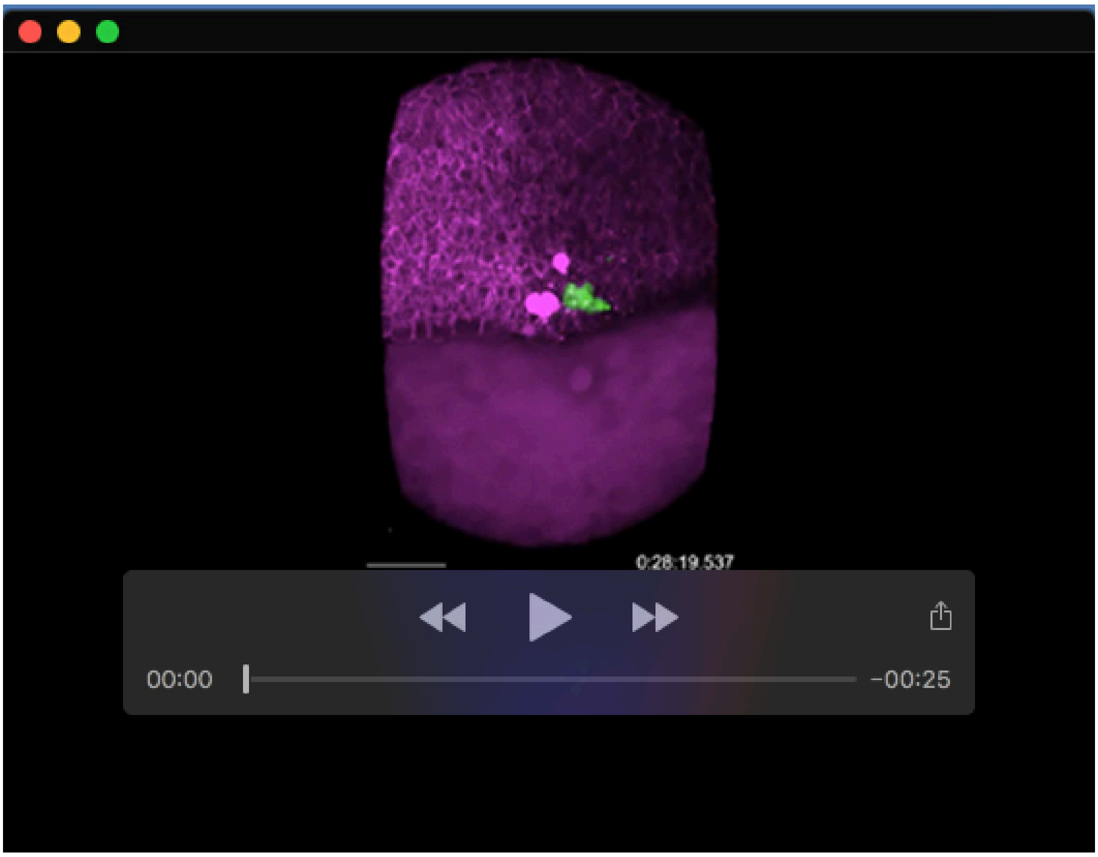

Movie 4. *hoxb7a*-overexpressing cells transplanted to the lateral germ ring margin exhibited delayed ingression compared with those of control. Time-lapse fluorescence movie visualizing cell ingression of control (green) and *hoxb7a*-overexpressing (magenta) cells transplanted to the lateral germ ring margin at the 40% epiboly stage. Imaging was started at the 50% epiboly stage (lateral view). Cell membranes of host embryo were labelled by injection of memRFP mRNA. Time interval, 4 min 43 sec. Duration, 3 hours 18 min. Scale bar, 100 µm.

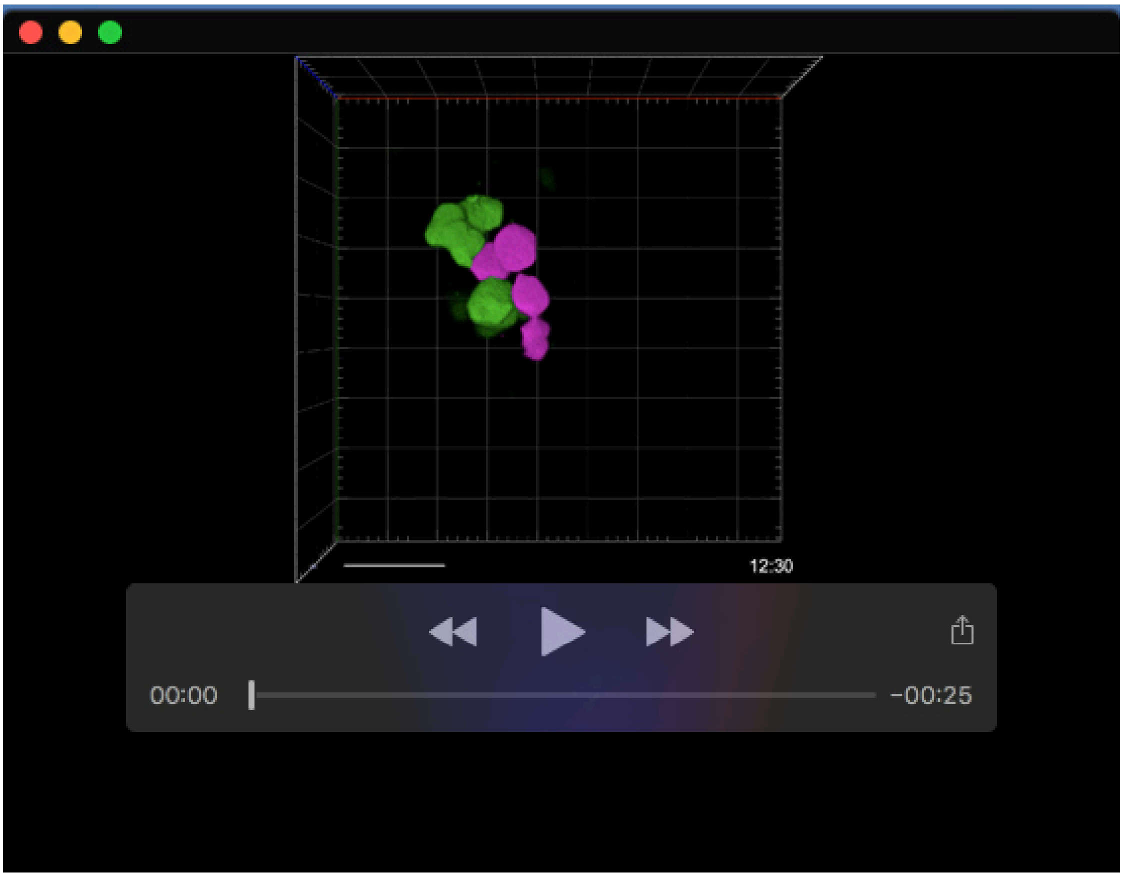

Movie 5. Transplanted control cells entered a frequent blebbing state, whereas co-transplanted *hoxb1b* morphant cells did not and instead exhibited a static state. Time-lapse fluorescence movie visualizing cell blebbing behavior of control (green) and *hoxb1b* morphant (magenta) cells transplanted to the lateral germ ring margin at the 40% epiboly stage. Imaging was started at the 50% epiboly stage (lateral view). Time interval, 30 sec. Duration, 20 min. Scale bar, 60 µm.

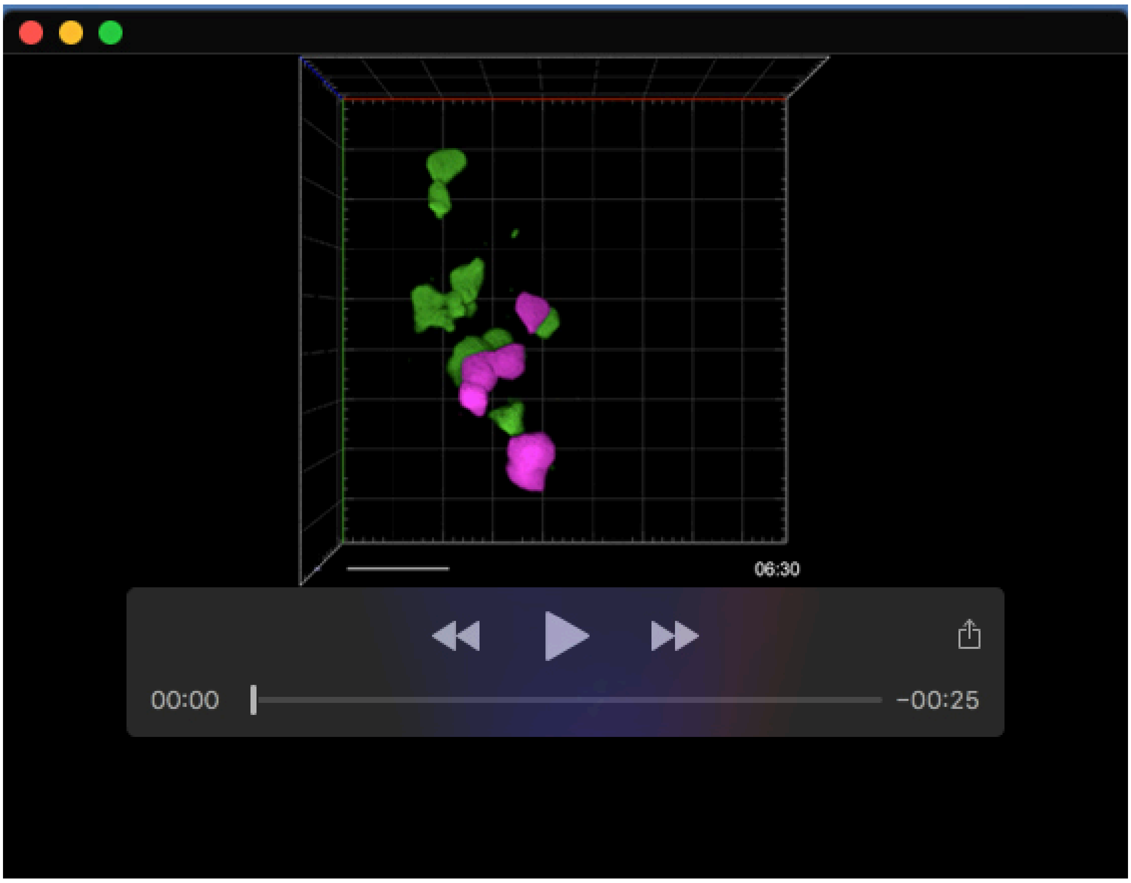

Movie 6. Transplanted *hoxb1b* morphant cells showed delayed entry into a frequent blebbing state compared with those of the control. Time-lapse fluorescence movie visualizing cell blebbing behavior for control (green) and *hoxb1b* morphant (magenta) cells transplanted to the lateral germ ring margin at the 40% epiboly stage. This movie captures the same sample as shown in Movie 5 and presents a consecutive series of images (lateral view). Time interval, 30 sec. Duration, 20 min. Scale bar, 60 μm.

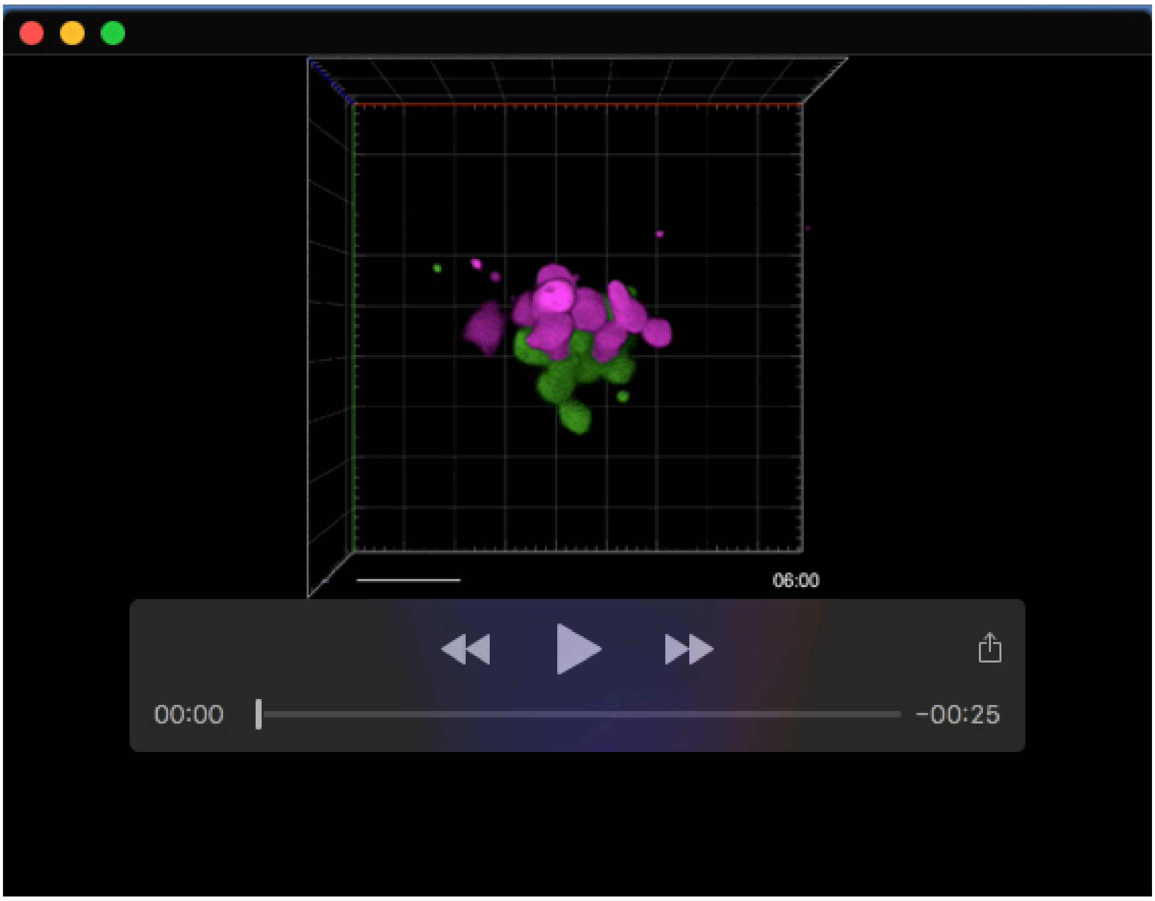

Movie 7. Control cells entered a frequent blebbing state, whereas *hoxb7a*-overexpressing cells did not and instead exhibited a static state. Time-lapse fluorescence movie visualizing cell blebbing behavior of control (green) and *hoxb1b* morphant (magenta) cells transplanted to the lateral germ ring margin at the 40% epiboly stage. Imaging was started at the 50% epiboly stage (lateral view). Time interval, 30 sec. Duration, 20 min. Scale bar, 60 μm.

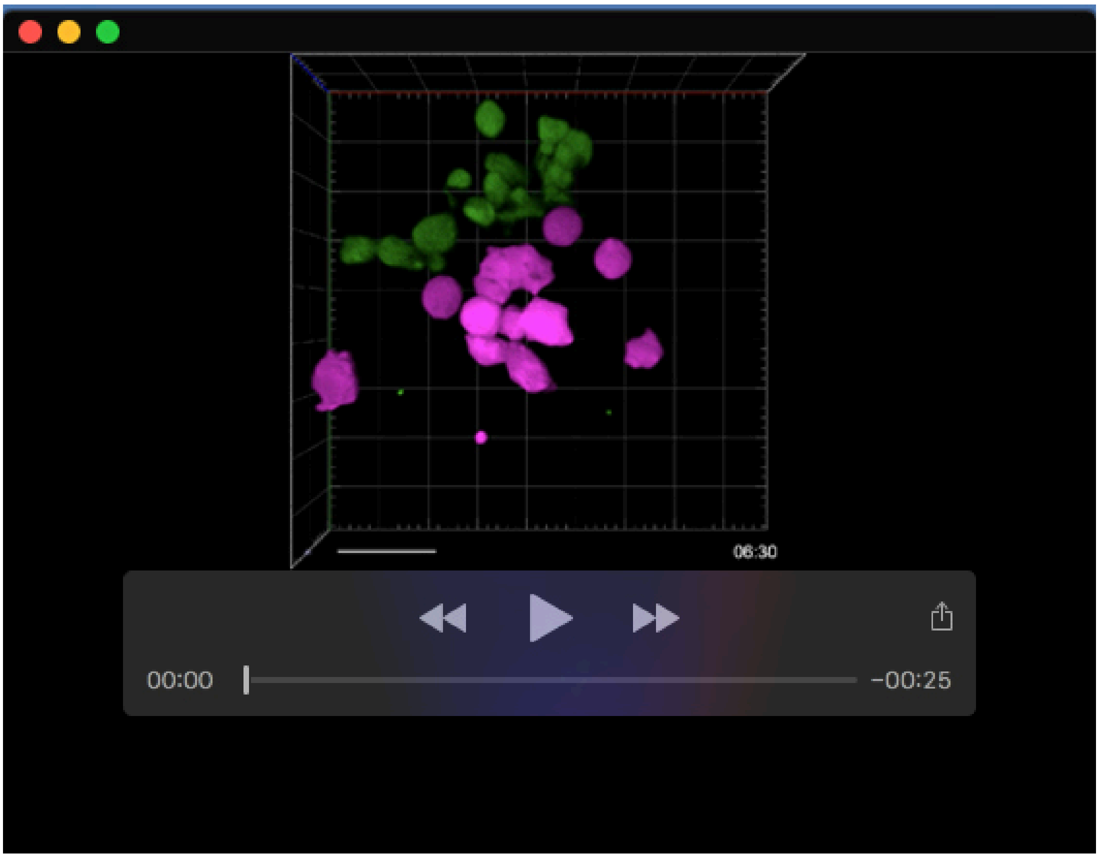

Movie 8. *hoxb7a*-overexpressing cells showed delayed entry into a frequent blebbing state compared with those of the control. Time-lapse fluorescence movie visualizing cell blebbing behavior of control (green) and *hoxb1b* morphant (magenta) cells transplanted to the lateral germ ring margin at the 40% epiboly stage. This movie captures the same sample as shown in Movie 7 and presents a consecutive series of images (lateral view). Time interval, 30 sec. Duration, 20 min. Scale bar, 60 µm.

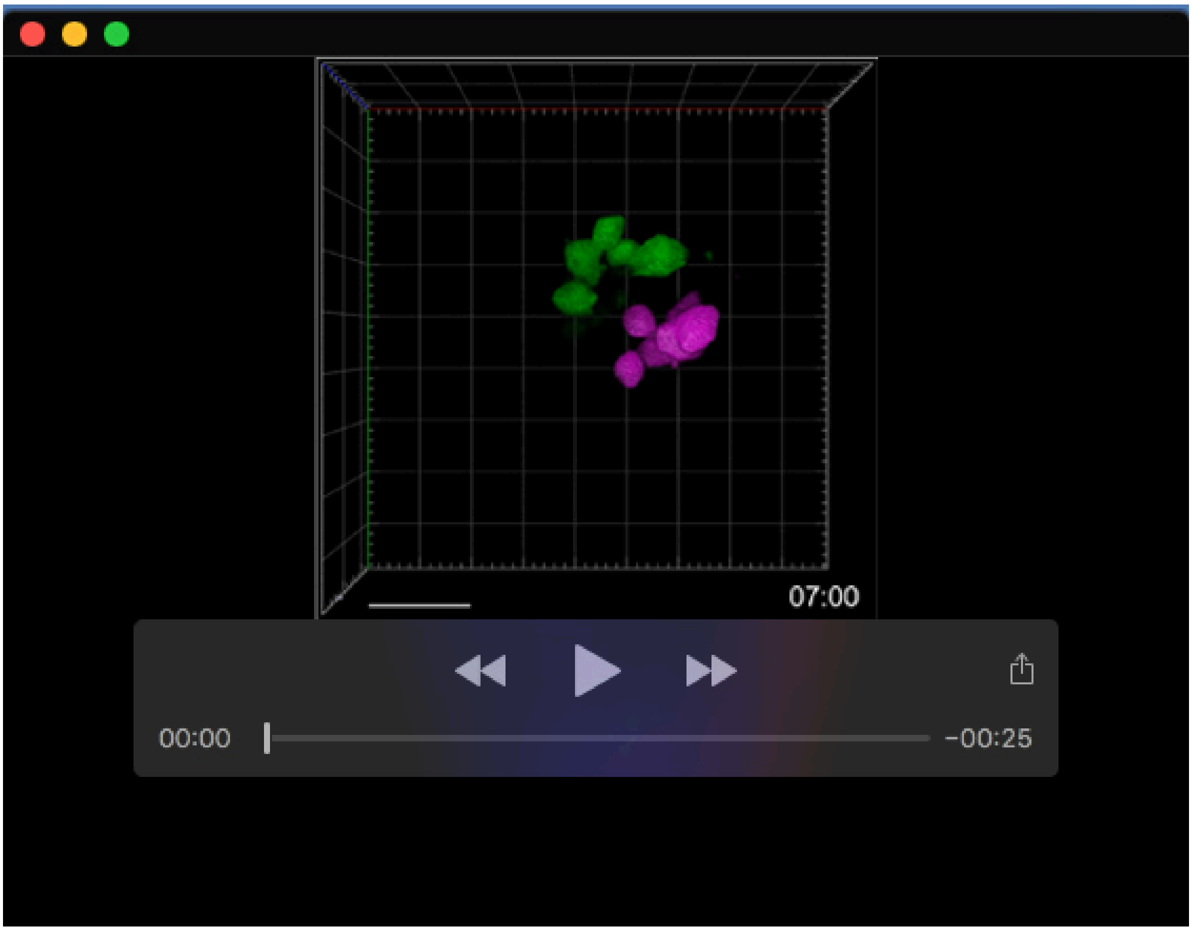

Movie 9. caRhoA-overexpressing *hoxb1b* morphant cells exhibited a rescued phenotype characterized by the timing of entry into a frequent blebbing state. Time-lapse fluorescence movie visualizing cell blebbing behavior of control (green) and caRhoA-injected *hoxb1b* morphant (magenta) cells transplanted to the lateral germ ring margin at the 40% epiboly stage. Imaging was started at the 50% epiboly stage (lateral view). Time interval, 30 sec. Duration, 20 min. Scale bar, 60 μm.
