# Supplementary material for: Hoxb genes determine the timing of cell ingression by regulating cell surface fluctuations during zebrafish gastrulation
Source: Development. 2025 Jun 27;152(12):dev204261. doi: 10.1242/dev.204261 (PMC12273642; doi:10.1242/dev.204261)
Supplement: Supplementary file 1 [file develop-152-204261_review_history.pdf]

hoxb genes determine the timing of cell ingression by regulating cell surface fluctuations during zebrafish gastrulation

Toshiyuki Mitsui, Carl-Philipp Heisenberg and Yuuta Moriyama
DOI: 10.1242/dev.204261

Editor: Thomas Lecuit

Review timeline
Original submission: 18 July 2024
Editorial decision: 8 October 2024
First revision received: 30 April 2025
Accepted: 22 May 2025

Original submission

First decision letter

MS ID#: dev.204261

MS Title: hoxb genes determine the timing of cell ingression by regulating cell surface fluctuations during zebrafish gastrulation

Authors: Yuuta Moriyama; Toshiyuki Mitsui; Carl-Philipp Heisenberg
Article Type: Research Article

Dear Dr Moriyama,

I sin merely apologise for the very unusual delay before getting back to you due to major difficulties finding available reviewers during the whole summer time. I have now received all the referees' reports on the above manuscript, and have reached a decision. The referees' comments are appended below, or you can access them online: please go to:

As you will see, the referees express considerable interest in your work, but have some important criticisms and recommend a revision of your manuscript before we can consider publication. If you are able to revise the manuscript along the lines suggested, which may involve further experiments, I will be happy receive a revised version of the manuscript. Your revised paper will be re-reviewed by one or more of the original referees, and acceptance of your manuscript will depend on your addressing satisfactorily the reviewers' major concerns. Please also note that Development will normally permit only one round of major revision. If it would be helpful, you are welcome to contact us to discuss your revision in greater detail. Please send us a point-by-point response indicating your plans for addressing the referees' comments, and we will look over this and provide further guidance.

Please attend to all of the reviewers' comments and ensure that you clearly highlight all changes made in the revised manuscript. Please avoid using 'Tracked changes' in Word files as these are lost in PDF conversion. I should be grateful if you would also provide a point-by-point response detailing how you have dealt with the points raised by the reviewers in the 'Response to Reviewers' box. If you do not agree with any of their criticisms or suggestions please explain clearly why this is so.

Reviewer 1

© 2025. Published by The Company of Biologists under the terms of the Creative Commons Attribution License (https://creativecommons.org/licenses/by/4.0/).

In their present study, Moriyama et al investigate the role of hoxb genes in regulation of cell ingression during zebrafish gastrulation. They find that hoxb genes are expressed with temporal collinearity at the embryo margin, resulting in spatial collinearity along the body axis. Using morpholino oligonucleotides and mRNA injection, they go on to show that timely expression of hoxb genes is required for timely cell ingression and normal gastrulation movements, and functions in a cell-autonomous manner. Based on imaging of transplanted cells, and global disruptions of cell contractility, the authors conclude that hoxb genes control the timing of cell ingression by regulating the frequency of blebbing and surface fluctuations in mesendodermal cells.

At large, this is an elegant study, and brings conceptual novelty in seeking to link the well-established concept of hox gene collinearity with single-cell behaviours and morphogenesis. The conclusion that collinear expression of hoxb genes is required for timely cell ingression is well supported by experimental data. However, I have some concerns, especially regarding the evidence that hoxb genes control cell surface fluctuations as stated in the manuscript title (main point 3).

Main points

1. The authors use colorimetric in situ hybridisation to profile expression patterns of hoxb genes, and conclude that expression is spatially and temporally collinear. As this is one of the main findings of the manuscript, I'm surprised they haven't used fluorescent multiplex in situ hybridisation to unambiguously compare expression patterns in single embryos, ie. FISH, RNA scope and HCR. These approaches are widely used in zebrafish embryos, and would make the conclusion of collinearity much more compelling. The authors should confirm their key observations using any of these fluorescent multiplexes in situ hybridisation techniques.

2. It is not clear how the authors conclude greater or lesser amount of mesendoderm ingression and anterior-directed migration in the hoxb MO or mRNA injection experiments. This seems to be from the distance of migrating mesendoderm from the germ ring, but how do they unequivocally define the leading edge of the mesendoderm? The authors should use a fluorescent reporter to mark the migrating cells and clearly show this. The authors should also include some simple measurements of these distances to show how representative the defects are across the dataset, ie. using the spline tool in ImageJ. A schematic in the figure could also help the reader.

3. The authors use Blebbistatin and RhoA overexpression to test whether blebbing is required for cell ingression at the margin. These treatments globally disrupt actomyosin contractility, so the apparent effect on ingression could equally be explained by disrupted mechanics elsewhere in the embryo. Can the authors specifically inhibit blebbing at the margin using refined and controlled manipulations?
Transplantation of cells expressing ca/dnRhoA into wild type hosts would better test their hypothesis using tools already used in the manuscript. Further, they should use genetic approaches to specifically disrupt mechanics in cells expressing specific hoxb genes to make the conclusion much more compelling.

4. The authors describe importance of collinear hoxb gene expression for timely cell ingression, but don't sufficiently reconcile the different effects of anterior and posterior hoxb genes. Posterior hoxb overexpression delays ingression - does this work by inhibiting blebbing? Or is blebbing in posterior cells unfavourable for internalisation? These possibilities could be resolved using techniques already applied in the manuscript and authors should address these questions.

Minor points

1. Figure panel labelling (Aa, Ab, Ac…) is unnecessarily dense, please consolidate.

2. The authors use expression of ntl to determine if hoxb misexpression affects mesendoderm specification, and conclude that there are no major changes. This isn't unambiguous based on the differences in embryo shape and size, and ntl intensity, visible in Fig. 3. Could the authors do some further analysis to support their argument? ie. quantification of cell number in the ntl domain, or some measurements of ntl domain size using their existing data?

© 2025. Published by The Company of Biologists under the terms of the Creative Commons Attribution License (https://creativecommons.org/licenses/by/4.0/).

3. Fig. 4 shows a very skilled fate mapping of cells ingressing at the dorsal margin. Are fluorescent images of the DiI signal available for the 0 and 30 minute conditions to better highlight the labelled cells?

4. Fig. 5G, N, U show tracks from 4D movies, but it's not clear what objects are being tracked according to the labels in B, I, P, and it's not convincing whether the same object is being marked in each frame. Have the authors tried automated tracking on these data? And can the movies be included in the manuscript with the tracks overlaid?

5. Lines 232-236. This sentence is very long and confusing. Please revise.

6. In the analysis of blebbing timing (page 14), what defines 'frequent blebbing'. The authors should make all such definitions explicit in the methods, especially if they are being used as landmarks for quantification - a table could work well for this.

7. In Fig. 6, the authors measure when cells ingress at the margin, but it's not convincing whether or not the labelled cells have ingressed or are just sat at the margin. It's not helped that the labelled cells are overexposed, which blurs their location. As for point 5, what is the definition for a cell having ingressed? please make it clear in the methods.

8. In general, Figure 7 is confusing because of the orthogonal views, please simplify. And what is the time relative to? What is 0? Would it be simpler to call the first panel 0, and then show the time elapsed to ingression in the second panel?

<u>Reviewer 2</u>

This manuscript investigates the role of hoxb cluster genes in regulating mesendoderm cell ingression during zebrafish gastrulation. They find that hoxb genes are expressed in a temporally collinear manner at the blastoderm margin, thereby guiding the internalization of mesendoderm cells and their anterior-posterior positioning later. Through both loss- and gain-of-function experiments, and validation with mutants, the authors demonstrate that perturbing hoxb gene expression disrupts the timing of cell ingression. They also establish a mechanistic link between hoxb genes and dynamic cell surface behaviors, particularly blebbing and surface fluctuations at the blastoderm margin. They find that hoxb genes regulate cell ingression through cell-autonomous mechanisms by modulating cell surface dynamics for successful and timely ingression. Overall, the manuscript is clear and well-written, with compelling evidence for the proposed model. A key area for improvement is a more in-depth comparison between the hoxb gene's temporal collinearity/blebbing-based ingression model and the Nodal signaling gradient/protrusive activity-based leader-follower model (Pinheiro et al.). A clearer explanation of when blebbing-based versus protrusive-based motility is deployed during ingression would be helpful for future studies. Are these mechanisms synergistic or competitive? A better discussion in this regard would be helpful. A specific concern is regarding the Blebbistatin and CA-Rho treatments used to modulate ingression. Do these treatments exclusively affect blebbing, or do they also influence protrusive leader-follower behaviors?

Below are my major comments, including suggestions for statistical reporting and additional analyses. These are not requests for new experiments but aimed at strengthening the current dataset:

1. General Comment: Please report the number of independent experiments and the number of embryos for all figures. While some figures indicate the number of embryos, the number of independent replicates is not marked.
2. Regarding Figure 2: Add statistical analyses for the observed phenotypes in all hoxb morphants.
3. Regarding Figure S2: Is there evidence or literature on cross-regulation among different hoxb genes? For instance, what are the expression profiles of hoxb4a, hoxb7a, and hoxb9a in the hox1b mutant, and vice versa?
4. Regarding Figure 3: Please provide statistics for the overexpression experiments. Additionally, I'm curious about the effects of hoxb4a, hoxb7a, and hoxb9a mRNA injections in the

© 2025. Published by The Company of Biologists under the terms of the Creative Commons Attribution License (https://creativecommons.org/licenses/by/4.0/).

respective mutant backgrounds. If hoxb expression timing is the sole critical factor, mRNA injection should not rescue the mutant phenotype. However, if expression levels matter (rather than timing), then mRNA at optimal concentrations may rescue these phenotypes.
5. Regarding Figure 5: Beyond timing, do hoxb genes influence the velocity of cell ingression? Velocities were measured for controls at 50% epiboly. Are the velocities slower for control cells at later stages when "late" hoxb genes are active? Relatedly, in Figure 5O-V, the average cell track in the epiblast appears longer compared to cells post-ingression. Why is this the case? Speculation would be useful here.

Minor Comments:
1. Line 110: Typo—correct "Results."
2. Figure 7G: The distinction between static and dynamic surface fluctuations is unclear. A clearer description in the main text would be helpful.
3. A supplemental movie visualizing CA-RhoA in hox1b morphants to specifically highlight the blebbing phenotype would add value to the findings.

Reviewer 3

SUMMARY OF THE ADVANCE MADE IN THIS PAPER AND ITS POTENTIAL SIGNIFICANCE TO THE FIELD

The manuscript "hoxb genes determine the timing of cell ingression by regulating cell surface fluctuations during zebrafish gastrulation" by Moriyama et al., studies the Hox spatial-temporal collinearity phenomenon during zebrafish gastrulation. By interfering with hoxb gene expression (knockdown, CRISPR-KO, and over-expression), this study shows that the orderly expression of hoxb gene controls mesendodermal cell ingression, with knockdown of the early isoform (hoxb1b) or premature expression of late hoxb genes (hoxb7a) controlling a cell-autonomous ingression at the onset of gastrulation. The late hoxb genes (hoxb4a, 7a, and 9a) have further roles in epiboly movements. Further, the authors show that the timing of mesendodermal cell ingression also directly leads to the positioning of these cells along the anterior-posterior body axis. The proposed mechanism by which hoxb genes control the timing of ingression is the fluctuations in the cell surface of mesendodermal cells (blebbing behaviour) which increases prior to ingression.

Overall, the functional studies of hoxb genes in zebrafish gastrulation is comprehensive and advances the understanding of vertebrate Hox spatial-temporal collinearity by providing a novel mechanism related with cell surface fluctuation. The transplantation experiments are particularly very effective to tease apart the cell autonomous effects of hoxb gene perturbations. Figures are well made and self-explanatory. Writing style is clear and easy to read and understand. The comments below will increase the clarity of some experimental results and improve this work on the mechanistic understanding of the process.

SUGGESTIONS TO AUTHORS

Major comments:
* The suppressive effects on mesendoderm ingression by the premature expression of the late hoxb isoform creates the question if these effects are via transcriptional competition with the early hoxb isoforms. Can the authors check how the gene expression of late and early hoxb genes are affected in the conditions where hoxb temporal collinearity is perturbed? For instance, to check expression patterns of hoxb4a/7a/9a in the absence of hoxb1b (do they come up earlier?), and the pattern of hoxb1b in the absence and o/e of hoxb4a/7a/9a (is it reduced?).
* If hoxb1 regulates cell blebbing in a cell autonomous manner, then cell blebbing during internalization should only be observed in the regions where hoxb1 is expressed. Can the authors compare cell blebbing behaviour in dorsal vs ventral side of the embryo?
* Although the rescue experiments of cell blebbing using LPA/caRhoA are convincing, the rescue of the overall embryo phenotype including ingression and epiboly are intriguing. How can the authors explain that these global manipulations do not affect epiboly movements, previously shown to depend on contractility (EVL surface tension reduction during spreading, anisotropic tissue tension during doming, YSL contractile ring promoting epiboly)? Along the same line, are the MO and mutant conditions for hoxb7a and 9a hypercontractile? The shape of the yolk at 90% epiboly and bud stage looks like there might be premature closing of the EVL.

© 2025. Published by The Company of Biologists under the terms of the Creative Commons Attribution License (https://creativecommons.org/licenses/by/4.0/).

* This study generates quite a few hoxb CRISPR KO lines. It would be quite powerful if the authors can demonstrate that in the KO lines, the timing of LPA/caRhoA treatment can similarly control the mesendoderm ingression. In general, are the mutant lines rescuable? And what is the percentage of embryos that manage to gastrulate?

Minor points:
* All the statistical significances were determined by two-tailed Student's t-test, which is not appropriate for multiple comparison (e.g. when the total group number is 3 or more and share the same control group data). This needs to be revisited and corrected.
* Fig 4 D, H, M and T - can the authors add to the graph that it is percentage of embryos with labelled cells completed ingression, so it is clearer what is meant by 'percentage of embryos'. Confocal images inset for MO conditions would be very helpful here.
* Fig 5 B-T: hard to see the small green arrowhead that is pointing at single cells.
* Fig 6B-D bit hard to orient oneself - maybe indicate the DV, AP axes?
* Can the authors discuss which downstream target genes hoxb genes might influence to affect the cell surface fluctuations? Are there some known targets that might be involved in these cellular processes?
* Check references (Line 100- The reference is missing, Line 6363-638; 650-653 - Duplicated reference)

First revision

Author response to reviewers' comments

Responses to the reviewers' comments - 1st revision (Initial Decision: 9 October 2024 / Resubmission: 28 April 2025)

We would like to thank all reviewers for their positive and constructive feedback. We have incorporated all their comments and suggestions, conducted additional experiments, and made extensive revisions to the manuscript and figures. One exception is a particular experiment requested in the first comment of Reviewer #1, for which - despite repeated attempts using various approaches - we were unable to obtain reliable results. As for the other points, all the data for figure 2 and 3, as well as much of the data for figure 7, have been re-acquired, and two more additional supplementary figures have been added through further experiments and analyses, as suggested by reviewers. Please find below our point-by-point responses. We have colored each reviewer's comments as shown below, matching the colors used in the revised manuscript. We also have indicated the page and line numbers for key changes as shown in the compiled PDF of the main text and figures.

Reviewer #1

In their present study, Moriyama et al investigate the role of hoxb genes in regulation of cell ingression during zebrafish gastrulation. They find that hoxb genes are expressed with temporal collinearity at the embryo margin, resulting in spatial collinearity along the body axis. Using morpholino oligonucleotides and mRNA injection, they go on to show that timely expression of hoxb genes is required for timely cell ingression and normal gastrulation movements, and functions in a cell-autonomous manner. Based on imaging of transplanted cells, and global disruptions of cell contractility, the authors conclude that hoxb genes control the timing of cell ingression by regulating the frequency of blebbing and surface fluctuations in mesendodermal cells.

At large, this is an elegant study, and brings conceptual novelty in seeking to link the well-established concept of hox gene collinearity with single-cell behaviours and morphogenesis. The conclusion that collinear expression of hoxb genes is required for timely cell ingression is well supported by experimental data. However, I have some concerns, especially regarding the evidence that hoxb genes control cell surface fluctuations as stated in the manuscript title (main point 3).

© 2025. Published by The Company of Biologists under the terms of the Creative Commons Attribution License (https://creativecommons.org/licenses/by/4.0/).

Main points

1. The authors use colorimetric in situ hybridisation to profile expression patterns of hoxb genes, and conclude that expression is spatially and temporally collinear. As this is one of the main findings of the manuscript, I'm surprised they haven't used fluorescent multiplex in situ hybridisation to unambiguously compare expression patterns in single embryos, ie. FISH, RNA scope and HCR. These approaches are widely used in zebrafish embryos, and would make the conclusion of collinearity much more compelling. The authors should confirm their key observations using any of these fluorescent multiplexes in situ hybridisation techniques.

We thank the reviewer for raising this point. We fully recognize that fluorescent multiplex *in situ* hybridization is a powerful technique for comparing the expression patterns of multiple genes within individual embryos. In line with this, we attempted to assess the combined expression patterns of different *hoxb* genes at the somite stage using both FISH and RNAscope approaches. However, these efforts did not yield satisfactory results that would allow clear visualization of the simultaneous expression of two *hoxb* genes. Despite this limitation, we believe that the spatial collinearity of *hoxb* gene expression during the somite stage can still be appreciated through the single-gene *in situ* hybridization data presented in Fig. S1J-N. Furthermore, our study primarily focuses on the temporal collinearity of *hoxb* gene expression, which we have effectively examined using conventional colorimetric *in situ* hybridization (Fig. 1). At the somite stage, our emphasis shifts toward analyzing the distribution patterns of ingressed cells (Fig. 6F–J), rather than the *hoxb* gene expression profiles themselves.

2. It is not clear how the authors conclude greater or lesser amount of mesendoderm ingression and anterior-directed migration in the hoxb MO or mRNA injection experiments. This seems to be from the distance of migrating mesendoderm from the germ ring, but how do they unequivocally define the leading edge of the mesendoderm? The authors should use a fluorescent reporter to mark the migrating cells and clearly show this. The authors should also include some simple measurements of these distances to show how representative the defects are across the dataset, ie. using the spline tool in ImageJ. A schematic in the figure could also help the reader.

We thank the reviewer for highlighting this important point. To quantitatively assess the phenotypes in *hoxb* morpholino (MO)-injected knockdown (KD) and mRNA-injected overexpression (OE) embryos, we employed the Tg(-1.8gsc:GFP) transgenic line, which allows visualization of ingressed mesendodermal cells. For the analysis of defects in cell ingression and anterior-directed migration in *hoxb* KD and OE embryos, we measured the distance of GFP- positive ingressed cell populations from the germ ring. This distance was then normalized by calculating the ratio relative to the total length from the blastoderm margin to the animal pole at 7 hours post-fertilization (hpf). To quantify epiboly delay phenotypes in *hoxb* KD and OE embryos, we measured the length from the blastoderm margin to the animal pole, and normalized it by the total length from the vegetal pole to the animal pole at 9 hpf. As suggested, we have included schematic representations of these measurements in the corresponding figures. Furthermore, we extended this analysis to *hoxb* mutants (knockout, KO). To this end, we crossed *hoxb* mutants with the Tg(-1.8gsc:GFP) line to generate *hoxb*$^{-/-}$; Tg(-1.8gsc:GFP) embryos. We then performed the same quantitative analyses on KO embryos as conducted for KD and OE conditions. These analyses allowed us to comprehensively quantify phenotypes related to cell ingression and epiboly progression across *hoxb* KD, KO, and OE embryos (see Fig. 2, Fig. 3; page 7, lines 156– 160; page 8, lines 169–172; Fig. S2E-K). Additionally, we performed analogous quantifications for related experiments involving phenocopy and rescue conditions using Blebbistatin, LPA, caMypt1, dnEzrin, and individual *hoxb* mRNAs (see Fig. 7H–R, Fig. S4F–N).

Please note that a similar concern was also raised by Reviewer #2, point 2.

3. The authors use Blebbistatin and RhoA overexpression to test whether blebbing is required for cell ingression at the margin. These treatments globally disrupt actomyosin contractility, so the apparent effect on ingression could equally be explained by disrupted mechanics elsewhere in the embryo. Can the authors specifically inhibit blebbing at the margin using refined and controlled manipulations?

Transplantation of cells expressing ca/dnRhoA into wild type hosts would better test their hypothesis using tools already used in the manuscript. Further, they should use genetic

© 2025. Published by The Company of Biologists under the terms of the Creative Commons Attribution License (https://creativecommons.org/licenses/by/4.0/).

approaches to specifically disrupt mechanics in cells expressing specific hoxb genes to make the conclusion much more compelling.

To further investigate this point, we performed transplantation experiments using cells expressing constitutively active RhoA (caRhoA) to assess its cell-autonomous role in mesendoderm ingression (Fig. 6, 7). As suggested, we additionally employed mRNA encoding either constitutively active Mypt1 (caMypt1) or dominant-negative Ezrin (dnEzrin) - which are known to suppress and promote cell blebbing, respectively - to test our hypothesis that cell blebbing regulates the timing of cell ingression. Wild-type embryos injected with caMypt1 mRNA displayed phenotypes similar to those observed in *hoxb1b* knockdown or knockout (KD/KO) embryos (Fig. 7K). To explore this further, we transplanted caMypt1 mRNA-injected wild-type cells into wild-type host embryos and analyzed their behavior, focusing on the timing of ingression, the onset of frequent blebbing, and their spatial distribution within somites following gastrulation. These transplanted cells exhibited delayed ingression, a longer latency before the initiation of frequent blebbing, and a more posterior localization compared to control morpholino (MO)-injected cells. Conversely, injection of dnEzrin mRNA into *hoxb1b* KD embryos rescued the defective phenotypes (Fig. 7P). Together with our previous results - including Blebbistatin treatment in wild- type embryos, and either LPA treatment or caRhoA mRNA injection in *hoxb1b* KD embryos – these findings collectively support our hypothesis that cell blebbing plays a crucial role in regulating the timing of cell ingression.

4. The authors describe importance of collinear hoxb gene expression for timely cell ingression, but don't sufficiently reconcile the different effects of anterior and posterior hoxb genes. Posterior hoxb overexpression delays ingression - does this work by inhibiting blebbing? Or is blebbing in posterior cells unfavourable for internalisation? These possibilities could be resolved using techniques already applied in the manuscript and authors should address these questions.

We thank the reviewer for raising this point. In our initial analysis, we examined the cell blebbing behavior in *hoxb7a* overexpressing (OE) cells and observed that these cells exhibited delayed blebbing, similar to the phenotype seen in *hoxb1b* knockdown (KD) cells (Fig. 7D–F). As suggested, to further investigate this aspect, we conducted additional experiments aimed at rescuing the phenotypes of *hoxb4a*, *hoxb7a*, and *hoxb9a* mutant embryos by treating them with lysophosphatidic acid (LPA), a compound known to enhance cortical contractility and promote cell blebbing. These experiments demonstrated that LPA treatment successfully rescued the phenotypes associated with the *hoxb* mutants (page 17, lines 380–386; Fig. S7A–H). These findings suggest that cell blebbing acts as a common downstream effector mechanism through which distinct *hoxb* genes regulate mesendoderm cell ingression.

Please note that a similar concern was also raised by Reviewer #3, point 4.

5. Figure panel labelling (Aa, Ab, Ac…) is unnecessarily dense, please consolidate.

We have standardized the size and positioning of the panel labels across the figures. However, with regard to the detailed labeling scheme using combinations of uppercase and lowercase letters (e.g., Aa, Ab, Ac), we have retained this format as each label corresponds to specific panels that are referenced individually in the manuscript text (e.g., page 6, lines 123–124). Therefore, altering the labeling would compromise clarity and consistency in referencing.

6. The authors use expression of ntl to determine if hoxb misexpression affects mesendoderm specification, and conclude that there are no major changes. This isn't unambiguous based on the differences in embryo shape and size, and ntl intensity, visible in Fig. 3. Could the authors do some further analysis to support their argument? ie. quantification of cell number in the ntl domain, or some measurements of ntl domain size using their existing data?

We have now included a quantitative analysis of the *ntl* domain size ratio and confirmed that there are no significant differences between *hoxb* overexpressing (OE) embryos (middle/posterior) and control embryos at 6 hpf (Fig. S4E; page 10, line 218-222).

7. Fig. 4 shows a very skilled fate mapping of cells ingressing at the dorsal margin. Are fluorescent images of the DiI signal available for the 0 and 30 minute conditions to better highlight the labelled cells?

As requested, we have added fluorescent DiI images for the 0- and 30-minute conditions, along with fluorescent image insets for all panels in Figure 4.

Please note that a similar concern was also raised by Reviewer #3, point 6.

© 2025. Published by The Company of Biologists under the terms of the Creative Commons Attribution License (https://creativecommons.org/licenses/by/4.0/).

8. Fig. 5G, N, U show tracks from 4D movies, but it's not clear what objects are being tracked according to the labels in B, I, P, and it's not convincing whether the same object is being marked in each frame. Have the authors tried automated tracking on these data? And can the movies be included in the manuscript with the tracks overlaid?
In the present manuscript, we determined the center of gravity of cells based on their contours and manually tracked their migration. To establish the appropriate time interval (Δt) for tracking cells between successive time points, we first assessed the experimental conditions and identified 3 minutes as the optimal interval. As cell contours were visualized by labeling the intercellular spaces, automated tracking proved to be very challenging using this dataset. We have now included the corresponding movie in the revised manuscript to support this analysis (Movie 1; page 13, line 285).

9. Lines 232-236. This sentence is very long and confusing. Please revise.
We have now revised the text to make it easier to understand (page 12/line 264-267).

10. In the analysis of blebbing timing (page 14), what defines 'frequent blebbing'. The authors should make all such definitions explicit in the methods, especially if they are being used as landmarks for quantification - a table could work well for this.
We have now revised the text explaining what we mean with 'frequent blebbing' and added an explanation in the figure (page 15/line 342-345, Fig. 7F).

11. In Fig. 6, the authors measure when cells ingress at the margin, but it's not convincing whether or not the labelled cells have ingressed or are just sat at the margin. It's not helped that the labelled cells are overexposed, which blurs their location. As for point 5, what is the definition for a cell having ingressed? please make it clear in the methods.

In the present manuscript, we defined an ingressed cell as one positioned within the hypoblast at the blastoderm margin (indicated by red cells in the figure on the right). Conversely, cells located within the epiblast at the margin are classified as *non-ingressed* (green cells in the same figure). We have revised the text in the manuscript to clarify this definition (page 13, lines 295–296). Regarding the occasional overexposure of magenta cells, this results from the need to enhance the visibility of cell membrane signals. Importantly, this overexposure did not affect our ability to accurately determine cell positions.

NOTE: We have removed unpublished data that had been provided for the referees in confidence.

12. In general, Figure 7 is confusing because of the orthogonal views, please simplify. And what is the time relative to? What is 0? Would it be simpler to call the first panel 0, and then show the time elapsed to ingression in the second panel?
We have revised Figure 7 to enhance clarity and simplify its presentation. In Figure 7F, the time point labeled "0" corresponds to the start of imaging. Additionally, we have updated the labeling of the Y-axis and revised the corresponding text in the manuscript for consistency and clarity (Fig. 7F; legends of Movie 5 and 7).

Reviewer #2

This manuscript investigates the role of hoxb cluster genes in regulating mesendoderm cell ingression during zebrafish gastrulation. They find that hoxb genes are expressed in a temporally collinear manner at the blastoderm margin, thereby guiding the internalization of mesendoderm cells and their anterior-posterior positioning later. Through both loss- and gain-of-function experiments, and validation with mutants, the authors demonstrate that perturbing hoxb gene expression disrupts the timing of cell ingression. They also establish a mechanistic link between hoxb genes and dynamic cell surface behaviors, particularly blebbing and surface fluctuations at the blastoderm margin. They find that hoxb genes regulate cell ingression through cell-autonomous mechanisms by modulating cell surface dynamics for successful and timely ingression. Overall, the manuscript is clear and well-written, with compelling evidence for the proposed model. A key area for improvement is a more in-depth comparison between the hoxb gene's

© 2025. Published by The Company of Biologists under the terms of the Creative Commons Attribution License (https://creativecommons.org/licenses/by/4.0/).

temporal collinearity/blebbing-based ingression model and the Nodal signaling gradient/protrusive activity-based leader-follower model (Pinheiro et al.). A clearer explanation of when blebbing-based versus protrusive-based motility is deployed during ingression would be helpful for future studies. Are these mechanisms synergistic or competitive? A better discussion in this regard would be helpful. A specific concern is regarding the Blebbistatin and CA-Rho treatments used to modulate ingression. Do these treatments exclusively affect blebbing, or do they also influence protrusive leader-follower behaviors?

We thank the reviewer for the constructive comments on our manuscript. Regarding the relationship between the Nodal signaling gradient/protrusive activity-based leader-follower model proposed by Pinheiro et al. and our present study, the main distinction lies in the region of the embryo being investigated. While Pinheiro et al. focused on the dorsal side, our study centers on the lateral blastoderm margin. To further address this point, we conducted a comparative analysis of cell dynamics during ingression between dorsal and lateral regions. Our findings indicate that these two cell populations ingress through distinct mechanisms (see Fig. S7I–O; page 17, lines 387–404; page 21, lines 467–469). Regarding the effects of Blebbistatin and constitutively active RhoA (caRhoA) on protrusion formation, it has been previously reported that both Blebbistatin and RhoA regulate cellular protrusiveness (as reviewed by Vicente-Manzanares *et al.*, *Nature Reviews Molecular Cell Biology*, 2009). We acknowledge the relevance of this point and plan to investigate it further in future studies.

Below are my major comments, including suggestions for statistical reporting and additional analyses. These are not requests for new experiments but aimed at strengthening the current dataset:

1. General Comment: Please report the number of independent experiments and the number of embryos for all figures. While some figures indicate the number of embryos, the number of independent replicates is not marked.

We have now added the number of analyzed samples (*n* – individual cells or embryos analyzed) and biological replicates (*N* – independent experiments analyzed) for all figures. We have also included a more explicit definition of what we describe as n and N in the material and methods section (page 31/line 686–687).

2. Regarding Figure 2: Add statistical analyses for the observed phenotypes in all hoxb morphants.

We thank the reviewer for highlighting this important point. To quantify the phenotypes observed in *hoxb* knockdown (KD) embryos, we utilized the Tg(-1.8gsc:GFP) transgenic line, which allows visualization of ingressed mesendodermal cells. For the analysis of cell ingression defects in *hoxb* KD embryos, we measured the distance of GFP-positive ingressed cell populations from the germ ring margin and calculated the ratio relative to the distance from the blastoderm margin to the animal pole at 7 hours post-fertilization (hpf). To quantify the epiboly delay phenotype, we measured the distance from the blastoderm margin to the animal pole and normalized it against the total distance from the vegetal to the animal pole at 9 hpf. We have added schematic illustrations to the figure to clarify these analyses (page 7, lines 156–160; page 8, lines 169–172; Fig. 2G, H).

Please note that a similar concern was also raised by Reviewer #1, point 2.

3. Regarding Figure S2: Is there evidence or literature on cross-regulation among different hoxb genes? For instance, what are the expression profiles of hoxb4a, hoxb7a, and hoxb9a in the hox1b mutant, and vice versa?

Yes, there are some reports of cross-regulation of *hoxb* genes during embryonic development. We have now added the description for this point and added the references (page 9/line 187–201, references: Arcioni *et al.*, 1992; Maconochie *et al.*, 1997; Manzanares *et al.*, 2001; Miller *et al.*, 2001; Popperl *et al.*, 1995; Studer *et al.*, 1998). To further investigate potential regulatory interactions, we examined the expression patterns of *hoxb4a*, *hoxb7a*, and *hoxb9a* in *hoxb1b* mutant embryos during gastrulation. Our results show that *hoxb4a* initiates expression at the blastoderm margin at 7 hpf (approximately 50% epiboly in *hoxb1b* mutants), while *hoxb7a* and *hoxb9a* begin expression at 7.6 hpf (approximately 60% epiboly). These findings suggest that, under *hoxb1b* knockout conditions, *hoxb4a*, *hoxb7a*, and *hoxb9a* are expressed in a time-

© 2025. Published by The Company of Biologists under the terms of the Creative Commons Attribution License (https://creativecommons.org/licenses/by/4.0/).

dependent rather than an epiboly progression-dependent manner during gastrulation. Additionally, we assessed the expression of *hoxb1b* in *hoxb4a*, *hoxb7a*, or *hoxb9a* mutant or overexpressing (OE) embryos at the 50% epiboly stage. We found that *hoxb1b* continues to be expressed at the blastoderm margin, similar to wild-type embryos, indicating that the transcription of *hoxb1b* is not affected by the altered expression of these other *hoxb* genes (page 9, lines 187–201; Fig. S3A– J).

Please note that a similar concern was also raised by Reviewer #3, point 1.

4. Regarding Figure 3: Please provide statistics for the overexpression experiments. Additionally, I'm curious about the effects of hoxb4a, hoxb7a, and hoxb9a mRNA injections in the respective mutant backgrounds. If hoxb expression timing is the sole critical factor, mRNA injection should not rescue the mutant phenotype. However, if expression levels matter (rather than timing), then mRNA at optimal concentrations may rescue these phenotypes.

For the overexpression experiments, we have now included quantitative analysis of the phenotypes using the Tg(-1.8gsc:GFP) line, in the same manner as for the knockdown (KD) experiments (Fig. 3). Additionally, we performed the same quantification on *hoxb* knockout (KO) mutants, consistent with our analyses for *hoxb* KD and overexpression (OE) embryos (Fig. S2E– K). Regarding the second point, we thank the reviewer for highlighting this intriguing aspect of the rescue experiments. We conducted rescue experiments by injecting *hoxb4a*, *hoxb7a*, or *hoxb9a* mRNA into their respective mutant backgrounds. However, we found that none of these mRNAs rescued the mutant phenotypes. Instead, they induced defects similar to those observed when the same *hoxb* mRNAs were injected into wild-type embryos (page 10, lines 227–235; Fig. S4F– N). These results suggest that the timing of *hoxb* gene expression - rather than merely the expression level - is critical for proper cell ingression and epiboly movements during gastrulation. Ectopic expression of mid- or late-acting *hoxb* genes at the onset of gastrulation appears to disrupt these developmental processes, even in *hoxb4a*, *hoxb7a*, and *hoxb9a* mutant embryos.

5. Regarding Figure 5: Beyond timing, do hoxb genes influence the velocity of cell ingression? Velocities were measured for controls at 50% epiboly. Are the velocities slower for control cells at later stages when "late" hoxb genes are active? Relatedly, in Figure 5O-V, the average cell track in the epiblast appears longer compared to cells post-ingression. Why is this the case? Speculation would be useful here.
To address this point, we divided our live imaging data into an early phase (0 – 27 min) and a later phase (30 – 60 min), and quantified and compared cell migration speeds in each period. Based on the imaging data, the early phase (0 – 27 min) corresponds to the stages from 50% epiboly to the shield stage, while the later phase (30 – 60 min) spans from the shield stage to post-60% epiboly (Fig. 5B-F). Therefore, the analysis compares the velocities of cell populations at the respective time points when *hoxb1b* and *hoxb4a*/*hoxb7a*/*hoxb9a* – representing early and mid/late-expressed *hoxb* genes – are expressed. As a result, no significant difference was observed between the two phases (right figure). For the average cell track in the epiblast appears longer compared to cells post-ingression in control, *hoxb1b* MO and *hoxb7a* mRNA injected embryos, it is considered to reflect the differences in cell dynamics between the epiblast and hypoblast. Indeed, in the revised manuscript, we now have examined the cell dynamics in epiblast and hypoblast, and found that cell dynamics differ between epiblast (pre-ingression at blastoderm margin) and hypoblast (during and post-ingression) (page 17/line 387–404, Fig. 7A-F, Fig. S7I– O).

NOTE: We have removed unpublished data that had been provided for the referees in confidence.

Minor Comments:

6. Line 110: Typo—correct "Results."
Corrected.

7. Figure 7G: The distinction between static and dynamic surface fluctuations is unclear. A clearer description in the main text would be helpful.
Thank you for your comment. We agree that the term static and dynamic may have been unclear. To improve clarity, we have now revised the description to refer to the states as “before” and “after” entering the frequent blebbing phase. Additionally, we have now included a boxed text in

© 2025. Published by The Company of Biologists under the terms of the Creative Commons Attribution License (https://creativecommons.org/licenses/by/4.0/).

Fig. 7F to clarify the definition of frequent blebbing (page 15, lines 342–345; Fig. 7F).

Please note that a similar concern was also raised by Reviewer #1, point 10.

8. A supplemental movie visualizing CA-RhoA in hox1b morphants to specifically highlight the blebbing phenotype would add value to the findings.
We have added a supplementary movie illustrating the blebbing phenotype in *caRhoA*-injected *hoxb1b* morphant cells (Movie 9; page 17, line 377).

Reviewer 3: SUMMARY OF THE ADVANCE MADE IN THIS PAPER AND ITS POTENTIAL SIGNIFICANCE TO THE FIELD

The manuscript "hoxb genes determine the timing of cell ingression by regulating cell surface fluctuations during zebrafish gastrulation" by Moriyama et al., studies the Hox spatial-temporal collinearity phenomenon during zebrafish gastrulation. By interfering with hoxb gene expression (knockdown, CRISPR-KO, and over-expression), this study shows that the orderly expression of hoxb gene controls mesendodermal cell ingression, with knockdown of the early isoform (hoxb1b) or premature expression of late hoxb genes (hoxb7a) controlling a cell-autonomous ingression at the onset of gastrulation. The late hoxb genes (hoxb4a, 7a, and 9a) have further roles in epiboly movements. Further, the authors show that the timing of mesendodermal cell ingression also directly leads to the positioning of these cells along the anterior-posterior body axis. The proposed mechanism by which hoxb genes control the timing of ingression is the fluctuations in the cell surface of mesendodermal cells (blebbing behaviour) which increases prior to ingression.

Overall, the functional studies of hoxb genes in zebrafish gastrulation is comprehensive and advances the understanding of vertebrate Hox spatial-temporal collinearity by providing a novel mechanism related with cell surface fluctuation. The transplantation experiments are particularly very effective to tease apart the cell autonomous effects of hoxb gene perturbations. Figures are well made and self-explanatory. Writing style is clear and easy to read and understand. The comments below will increase the clarity of some experimental results and improve this work on the mechanistic understanding of the process.

SUGGESTIONS TO AUTHORS

Major comments:

1. The suppressive effects on mesendoderm ingression by the premature expression of the late hoxb isoform creates the question if these effects are via transcriptional competition with the early hoxb isoforms. Can the authors check how the gene expression of late and early hoxb genes are affected in the conditions where hoxb temporal collinearity is perturbed? For instance, to check expression patterns of hoxb4a/7a/9a in the absence of hoxb1b (do they come up earlier?), and the pattern of hoxb1b in the absence and o/e of hoxb4a/7a/9a (is it reduced?).

We have now examined the expression patterns of *hoxb4a*, *hoxb7a*, and *hoxb9a* in *hoxb1b* mutant embryos during gastrulation. Our analysis revealed that *hoxb4a* begins expression at the blastoderm margin at 7 hpf (corresponding to 50% epiboly in *hoxb1b* mutants), while *hoxb7a* and *hoxb9a* initiate expression at 7.6 hpf (approximately 60% epiboly). These results suggest that, under *hoxb1b* knockout conditions, the expression of *hoxb4a*, *hoxb7a*, and *hoxb9a* is regulated in a time-dependent rather than an epiboly progression-dependent manner during gastrulation. In addition, we examined the expression of *hoxb1b* in *hoxb4a*, *hoxb7a*, and *hoxb9a* mutant or overexpression embryos at the 50% epiboly stage. We found that *hoxb1b* remains expressed at the blastoderm margin similarly to wild-type embryos, suggesting that the altered expression of *hoxb4a*, *hoxb7a*, and *hoxb9a* does not affect *hoxb1b* transcription (page 9, lines 187–201; Fig. S3A-J). We have also added references to previous studies reporting cross-regulatory interactions among Hox genes during development (Arcioni et al., 1992; Maconochie et al., 1997; Manzanares et al., 2001; Miller et al., 2001; Popperl et al., 1995; Studer et al., 1998).

Please note that a similar concern was also raised by Reviewer #2, point 3.

© 2025. Published by The Company of Biologists under the terms of the Creative Commons Attribution License (https://creativecommons.org/licenses/by/4.0/).

2. If hoxb1 regulates cell blebbing in a cell autonomous manner, then cell blebbing during internalization should only be observed in the regions where hoxb1 is expressed. Can the authors compare cell blebbing behaviour in dorsal vs ventral side of the embryo?

We thank the reviewer for highlighting this intriguing aspect. To address this point, we conducted transplantation experiments and compared cell dynamics between the dorsal and lateral regions of the embryo. Our study specifically focuses on the lateral blastoderm margin, where future somite cells reside. Our analysis revealed that cell behaviors - such as the frequency of bleb formation and the presence of filopodia or lamellipodia - differ between the dorsal and lateral regions. Notably, *hoxb1b* knockdown (KD) cells transplanted into the dorsal blastoderm margin did not show significant differences in behavior compared to control transplanted cells. This suggests that the blebbing-based ingression mechanism observed in *hoxb1b* KD cells is specific to the lateral blastoderm margin, where *hoxb1b* is normally expressed. In contrast, the dorsal region appears to utilize a different, protrusion-based ingression mechanism, consistent with previous findings (page 17, lines 387–404; Fig. S7I–O; Pinheiro et al., *Nature Physics*, 2022).

3. Although the rescue experiments of cell blebbing using LPA/caRhoA are convincing, the rescue of the overall embryo phenotype including ingression and epiboly are intriguing. How can the authors explain that these global manipulations do not affect epiboly movements, previously shown to depend on contractility (EVL surface tension reduction during spreading, anisotropic tissue tension during doming, YSL contractile ring promoting epiboly)? Along the same line, are the MO and mutant conditions for hoxb7a and 9a hypercontractile? The shape of the yolk at 90% epiboly and bud stage looks like there might be premature closing of the EVL.

We observed that global manipulation through LPA treatment or caRhoA overexpression successfully rescued the *hoxb1b* morphant epiboly phenotype (Figure 7M, O). As suggested, these phenotypic improvements may be associated with alterations in the mechanical properties of the embryo, such as changes in EVL (enveloping layer) surface tension, anisotropic tissue tension during the doming phase, or enhanced contraction of the yolk syncytial layer (YSL) actomyosin ring - factors known to promote epiboly. We plan to investigate these mechanistic aspects in future studies. Regarding the second point, as suggested, we also consider that the actomyosin rings in *hoxb7a* and *hoxb9a* KD/KO embryos may be hypercontractile, contributing to the characteristic phenotype involving extrusion of yolk from the blastopore. Given that YSL actomyosin ring contraction normally increases during epiboly progression (Behrndt et al., *Science*, 2012), we speculate that this phenotype arises from a disrupted balance between excessive YSL contractility and delayed epiboly in the *hoxb7a* and *hoxb9a* KD/KO embryos.

4. This study generates quite a few hoxb CRISPR KO lines. It would be quite powerful if the authors can demonstrate that in the KO lines, the timing of LPA/caRhoA treatment can similarly control the mesendoderm ingression. In general, are the mutant lines rescuable? And what is the percentage of embryos that manage to gastrulate?

We thank the reviewer for raising this very intriguing point. We have now tested whether LPA treatment, initiated at the 50% epiboly stage (just prior to the onset of gastrulation), can rescue the phenotypes observed in *hoxb4a*, *hoxb7a*, and *hoxb9a* mutant embryos. Our results show that LPA treatment successfully rescues the phenotypes across all three mutant backgrounds (page 17, lines 380–386; Fig. S7A–H). This finding suggests that not only the anterior *hoxb* gene (*hoxb1b*), but also the middle and posterior *hoxb* genes (*hoxb4a*, *hoxb7a*, and *hoxb9a*) promote cortical contractility or cell blebbing as a mechanism for cell ingression. Regarding the second point, we performed rescue experiments by injecting *hoxb4a*, *hoxb7a*, or *hoxb9a* mRNA into their respective mutant backgrounds. However, none of these injections were able to rescue the mutant phenotypes. This result indicates that proper temporal regulation of *hoxb* gene expression - rather than the amount of mRNA - is critical for morphogenesis during gastrulation (page 10, lines 227–235). In response to the third point, we assessed the proportion of embryos in each mutant background that successfully completed gastrulation. The results are as follows: *hoxb1b* mutants: 80.7% (n = 155/192, N = 3); *hoxb4a* mutants: 95.9% (n = 188/196, N = 3); *hoxb7a* mutants: 94.1% (n = 174/185, N = 3); *hoxb9a* mutants: 92.1% (n = 221/240, N = 3); Control wild-type (AB): 99.3% (n = 429/432, N = 2).

Please note that a similar concern was also raised by Reviewer #1, point 4 and Reviewer #2, point 4.

© 2025. Published by The Company of Biologists under the terms of the Creative Commons Attribution License (https://creativecommons.org/licenses/by/4.0/).

Minor points:

5. All the statistical significances were determined by two-tailed Student's t-test, which is not appropriate for multiple comparison (e.g. when the total group number is 3 or more and share the same control group data). This needs to be revisited and corrected.
We thank the reviewer for highlighting this important point. All statistical analyses have been carefully revised and re-conducted using appropriate and validated methods. A detailed description of the statistical procedures has been added to the Methods section (page 30, lines 678–687).

6. Fig 4 D, H, M and T - can the authors add to the graph that it is percentage of embryos with labelled cells completed ingression, so it is clearer what is meant by 'percentage of embryos'. Confocal images inset for MO conditions would be very helpful here.
We have revised the Y-axis labels in Figure 4D, H, M, and T to read: *"Percentage of embryos with labelled cells that completed ingression (%)."* In response to the second comment, we have added confocal image insets to all panels in Figure 4 to improve visualization of the labelled cells.

Please note that a similar concern was also raised by Reviewer #1, point 7.

7. Fig 5 B-T: hard to see the small green arrowhead that is pointing at single cells.
We have increased the arrowheads size in Fig. 5B–T.

8. Fig 6B-D bit hard to orient oneself - maybe indicate the DV, AP axes?
We have added the DV and AP axes in Fig. 6B–D.

9. Can the authors discuss which downstream target genes hoxb genes might influence to affect the cell surface fluctuations? Are there some known targets that might be involved in these cellular processes?
It has been reported that Hox proteins regulate *Epha4* and Eph/ephrin signaling (Prin et al., *Development*, 2014), and that EphA4 plays a role in controlling actomyosin contraction (Cayuso et al., *eLife*, 2019). Based on these findings, it is plausible that Hox genes influence cell surface fluctuations or blebbing behavior through the modulation of *Epha4* and Eph/ephrin signaling pathways. We have added a discussion of this point in the revised manuscript (page 20, lines 453–457).

10. Check references (Line 100- The reference is missing, Line 6363-638; 650-653 - Duplicated reference)
Corrected.

Second decision letter

MS ID#: dev.204261R1

MS Title: hoxb genes determine the timing of cell ingression by regulating cell surface fluctuations during zebrafish gastrulation

Authors: Yuuta Moriyama; Toshiyuki Mitsui; Carl-Philipp Heisenberg
Article Type: Research Article

Dear Yuuta,

I am happy to tell you that your manuscript has been accepted for publication in Development, pending our standard publication integrity checks. It was accepted on 22 May 2025. Where referee reports on this version are available, they are appended below.

Comments from the Reviewers:

© 2025. Published by The Company of Biologists under the terms of the Creative Commons Attribution License (https://creativecommons.org/licenses/by/4.0/).

Reviewer 1: I'm surprised that the authors were unable to get HCR or RNAscope to work. That said, the manuscript has improved overall, and the authors have made a sincere effort to address the comments.

Reviewer 2: SUMMARY OF THE ADVANCE MADE IN THIS PAPER AND ITS POTENTIAL SIGNIFICANCE TO THE FIELD

The manuscript has significantly improved and the authors have addressed major suggestions.

SUGGESTIONS TO AUTHORS

The manuscript is suitable for publication.

© 2025. Published by The Company of Biologists under the terms of the Creative Commons Attribution License (https://creativecommons.org/licenses/by/4.0/).
